# Generating experimentally unrelated target molecule-binding highly functionalized nucleic-acid polymers using machine learning

**DOI:** 10.1038/s41467-022-31955-4

**Published:** 2022-08-04

**Authors:** Jonathan C. Chen, Jonathan P. Chen, Max W. Shen, Michael Wornow, Minwoo Bae, Wei-Hsi Yeh, Alvin Hsu, David R. Liu

**Affiliations:** 1 Merkin Institute of Transformative Technologies in Healthcare, Broad Institute of Harvard and MIT, Cambridge, MA USA; 2 Department of Chemistry and Chemical Biology, Harvard University, Cambridge, MA USA; 3 Howard Hughes Medical Institute, Harvard University, Cambridge, MA USA; 4 Work conducted at Uber AI Labs, Uber Technologies, Inc., San Francisco, CA USA; 5 Meta Platforms, Menlo Park, CA USA; 6 Computational and Systems Biology Program, Massachusetts Institute of Technology, Cambridge, MA USA; 7 Program in Speech and Hearing Bioscience and Technology, Harvard Medical School, Boston, MA USA

**Keywords:** Molecular engineering, Chemical biology, Chemical libraries

## Abstract

In vitro selection queries large combinatorial libraries for sequence-defined polymers with target binding and reaction catalysis activity. While the total sequence space of these libraries can extend beyond 10^22^ sequences, practical considerations limit starting sequences to ≤~10^15^ distinct molecules. Selection-induced sequence convergence and limited sequencing depth further constrain experimentally observable sequence space. To address these limitations, we integrate experimental and machine learning approaches to explore regions of sequence space unrelated to experimentally derived variants. We perform in vitro selections to discover highly side-chain-functionalized nucleic acid polymers (HFNAPs) with potent affinities for a target small molecule (daunomycin *K*_D_ = 5–65 nM). We then use the selection data to train a conditional variational autoencoder (CVAE) machine learning model to generate diverse and unique HFNAP sequences with high daunomycin affinities (*K*_D_ = 9–26 nM), even though they are unrelated in sequence to experimental polymers. Coupling in vitro selection with a machine learning model thus enables direct generation of active variants, demonstrating a new approach to the discovery of functional biopolymers.

## Abstract (web summary)

In vitro library screening is a powerful approach to identify functional biopolymers, but only covers a fraction of possible sequences. Here, the authors use experimental in vitro selection results to train a conditional variational autoencoder machine learning model that generates biopolymers with no apparent sequence similarity to experimentally derived examples, but that nevertheless bind the target molecule with similar potent binding affinity.

## Introduction

In vitro selection—iterative cycles of selection, amplification, and occasional mutagenesis on large combinatorial libraries—is a well-established technique^1,2^ that enables the isolation of sequence-defined polymers with high levels of binding^3,4^ or catalytic activity^5–9^. When in vitro selection was first developed, limited sequencing capabilities restricted the identification of selection outcomes to only a modest number of active sequences. Subsequent developments in high-throughput sequencing (HTS) and lowered sequencing costs have made it possible to reconstruct broader fitness landscapes, revealing broader relationships between sequence and activity. Even state-of-the-art sequencing technologies, however, are still limited to ~10^10^ reads, which precludes full sequencing of the typical 10-1000 pmol (6 × 10^12^ to 6 × 10^14^ molecules) starting libraries used for in vitro selections. As a result, attempts to map sequences onto the global fitness landscape are typically limited to regions of local space from late-stage selection rounds, where the high convergence and low sequence diversity makes comprehensive identification of active sequences possible^10–12^.

Because most biopolymer fitness landscapes are rugged^12,13^, with multiple local optima, characterization of the global fitness landscape requires identification of multiple, distinct, active variants^14^. To address this challenge, improvements to in vitro selections have increased the probability of finding active sequences in a random, combinatorial library^15–18^ and made enrichment more efficient^19,20^. However, these approaches are still subject to limitations imposed by in vitro selection itself, sequence convergence, and limited sequencing depth. In the current study, we present an integrated approach for discovering diverse, highly active sequences unrelated to any previously known variants: direct generation using a machine learning model trained on in vitro selection data. High-activity sequences generated by this machine learning model have no apparent sequence similarity to experimental sequences used to train the model, suggesting that the model has learned the fitness landscape sufficiently well to generate novel yet highly active variants.

**Fig. 1:**
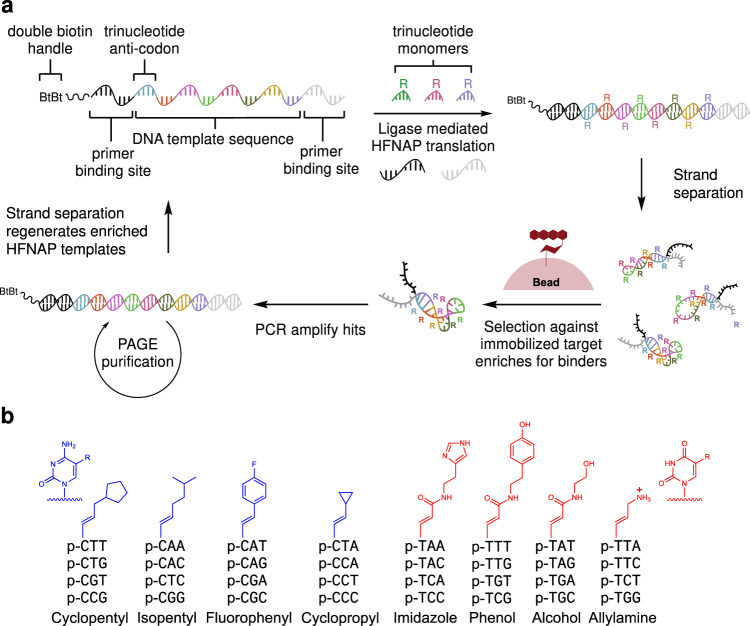
HFNAP building blocks, translation, and selection. **a** Overview of the HFNAP translation, selection against daunomycin, and reverse translation to regenerate DNA templates. **b** Structures of the 5'-phosphorylated trinucleotide monomer building blocks used in this selection, along with their associated, four-fold redundant DNA codons.

To obtain data sufficient for training, we conducted an optimized in vitro selection campaign on a chemically diverse starting library of sequence-defined side-chain containing synthetic polymers known as highly functionalized nucleic acid polymers (HFNAPs)^16,21^. HFNAPs consist of oligonucleotides with side-chains on every third nucleotide that are translated from DNA templates using a ligation-based translation system (Fig. 1a). Translation is mediated by T3 ligase and uses a set of customizable trinucleotide building blocks containing a 5' modified nucleobase with side-chains chosen by the researcher (Fig. 1b)^16,21^. In contrast with other non-natural polymer systems compatible with Darwinian selection that replace individual nucleotide triphosphates with functionalized variants^15^, or that use non-natural base pairs^17,22^, the HFNAP system uses 32 codons, which collectively can encode up to 32 distinct side-chains. The current iteration of the HFNAP translation system consists of 15 consecutive trinucleotide building blocks, each of which contains one of eight different side-chains encoded by 32 codons. While the codon system introduces modest constraints to the sequence space that can be explored, the resulting library’s side-chain diversity (8^15^ = 3.5 × 10^13^) is orders of magnitude larger than the side-chain diversity achievable with polymerase-synthesized functionalized oligonucleotides of the same sequence length. The enhanced chemical diversity of HFNAPs compared to conventional oligonucleotides improves the likelihood of isolating variants with desirable properties such as target protein binding^16,23^.

The use of machine learning to enable functional synthetic polymer discovery presents new challenges. Many machine learning-aided protein evolution efforts have relied extensively on evolved, active protein sequences that are often incorporated directly into the resulting sequence predictions^24,25^. When starting from protein variants with low but non-zero levels of activity, machine learning can incorporate previously solved protein structures^26^, or related protein sequences optimized over millions of years of natural evolution^27^. In contrast, in vitro selections start from naïve libraries^18,28^, resulting in sparse and noisy datasets in which active examples are vastly outnumbered by inactive sequences. Sequence convergence during selection also limits the sequence diversity of active variants. Moreover, low-throughput hit characterization often limits the number of sequences for which activity can be measured. In the absence of comprehensive ground-truth activity measurements from labor-intensive biochemical assays, enrichment values are used as a proxy, introducing substantial measurement noise as the correlation between enrichment values and desired activity can be modest, influenced by the stochasticity of selections and activity-independent biases that occur during translation, selection, or PCR amplification^29,30^. Finally, limited sequencing depth restricts the ability to sample and observe the evolutionary trajectories of large numbers of input sequences, resulting in a noisy view of already noisy data.

Despite the lack of optimal training data, we envisioned a model that could learn aspects of a fitness landscape sufficient to generate active sequences unrelated to the limited diversity of sequences that can be identified from an in vitro selection. Generating active sequences in this manner is difficult because the space of possible outputs is very large, while the space of correct outputs is comparatively small. In this study, we address these challenges experimentally using judiciously chosen selection conditions and a chemically diverse starting library of HFNAPs to acquire data of sufficient quality to train a machine learning model. These selection improvements increase enrichment efficiency, enabling the discovery of highly active sequences that bind daunomycin, a small-molecule chemotherapeutic^31–33^.

We then apply a generative machine learning approach to learn the joint probability distribution between HFNAP sequence space and daunomycin-binding affinity. Our trained model successfully uses data from a single in vitro selection to generate multiple diverse, highly active polymer sequences with no apparent sequence similarity to one another or to the sequences from the in vitro selection, in contrast with the tendency of individual selection campaigns to produce highly sequence-related hits. Analysis of predicted secondary structures for CVAE-generated sequences and in vitro selection sequences reveal secondary structure conservation that is not present among random sequences of the same composition, consistent with the machine learning model having achieved a broad understanding of the daunomycin-binding fitness landscape. These findings establish an integrated experimental and machine-learning approach to generate many sequence-defined polymer sequences with strong target binding activity, including those unrelated in primary sequence to any previously identified experimental variants, with broad implications for the discovery and application of non-natural biopolymers.

## Results

### HFNAP library and selection design

The HFNAP translation system (Fig. 1a) uses a DNA template with a 45-bp coding region containing 15 consecutive codons to recruit building blocks drawn from a set of 32 side-chain-functionalized trinucleotides (Fig. 1b). As the building blocks hybridize to their complementary codons^16,21^, T3 DNA ligase ligates their phosphodiester backbones into one continuous strand^34^. HFNAPs are then selectively eluted through biotin capture of the heteroduplex HFNAP–DNA double-stranded product followed by alkaline denaturation to remove the non-biotinylated HFNAP strand (Fig. 1a). The total sequence space in this library is 32^15^ = 3.8 × 10^22^ sequences.

Following DNA-templated translation of a 125 pmol starting template library (7.5 × 10^13^ molecules), we performed iterated rounds of in vitro selection for binding to immobilized daunomycin. Biotinylated daunomycin was synthesized by coupling a biotin linker to daunomycin’s primary amine and immobilized by incubating the product with streptavidin-linked magnetic beads (see Methods). To prevent selection of HFNAPs that bind the biotin linker or the streptavidin-linked beads, a negative selection step was introduced after round 4. Translated HFNAPs were incubated with a 1:1 mixture of blank streptavidin-linked beads and streptavidin-linked beads with immobilized negative selection linker (see Methods). The flow-through from this negative selection step was then subjected to positive selection for binding to immobilized daunomycin.

**Fig. 2:**
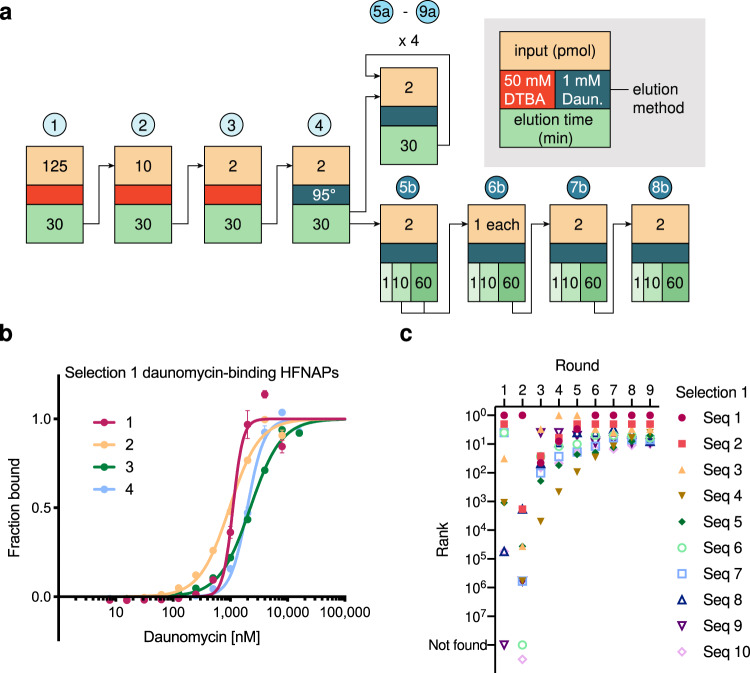
Selections against daunomycin yield HFNAPs with binding affinity. **a** Selection scheme and parameters are shown. Early rounds utilize reducing conditions to elute binders, to broadly capture HFNAPs. Subsequent rounds rely on target elution with 1 mM daunomycin. Selection 1 (rounds 1–4, 5a-9a) is denoted by the upper path, selection 2 (rounds 4, 5b-8b) by the lower path. **b** MST characterization of the binding affinity for the top 4 most enriched sequences from selection 1. Sequences were found to bind with *K*_d_ = 1–2 µM. Values and error bars reflect the mean and SEM of *n* = 3 independent replicates. Error bars for some values are too small to extend beyond the data point. **c** The evolutionary trajectory of the top ten most enriched sequences in selection 1.

After removal of the positive selection flow-through, beads were washed three times with selection buffer (see Methods). Bound HFNAPs were eluted by reduction of the disulfide bond connecting daunomycin and the biotin linker (rounds 1–3) or by target elution with 1 mM daunomycin for 30 min, followed by two washes with selection buffer (rounds 4 and 5a-9a) (Fig. 2a). The eluted HFNAPs were reverse translated in a PCR reaction using Q5 DNA polymerase, which we previously found to efficiently copy HFNAPs back into complementary DNA^21^. Full-length amplicons were purified by native polyacrylamide gel electrophoresis (PAGE). Template DNA strands were isolated by biotin capture and alkaline denaturation for the next round of HFNAP library translation and selection.

We performed nine iterated rounds of in vitro selection for daunomycin binding using the conditions summarized in Fig. 2a (selection 1, rounds 1–4 and 5a-9a). As the selection progressed, the ratio of eluted HFNAPs to HFNAPs in the flow-through increased, consistent with enrichment of daunomycin-binding HFNAPs (Supplementary Fig. 1). The sequence composition of the eluted HFNAPs at the end of each round of selection was analyzed by high-throughput DNA sequencing (HTS). We ranked sequences by overall frequency in round 9a, and the top four HFNAPs were synthesized by ligase-mediated DNA-templated translation of individual templates (see Methods). We characterized the binding affinity of these individual polymers for free daunomycin by microscale thermophoresis (MST) and found that these HFNAPs bound with modest affinity (*K*_d_ = ~1–2 µM) (Fig. 2b and Supplementary Table 1). These results revealed that modest affinity was sufficient to pass the initial selection campaign and suggested that selection stringency could be increased to yield more active polymers.

### Modifying the selection to increase stringency

The optimal selection stringency for each round of selection is a delicate balance between preserving sequence diversity and enabling efficient enrichment of active variants. High-stringency conditions applied too early can result in selection failure (no legitimate survivors) or excessive loss of sequence diversity before rare, high-activity sequences can be accessed^35^. Carefully increasing selection stringency minimizes the probability that the stochastic, error-prone, and biased nature of selection steps results in irreversible loss of sequences that may give rise to the top-performing variants at later rounds of selection.

**Fig. 3:**
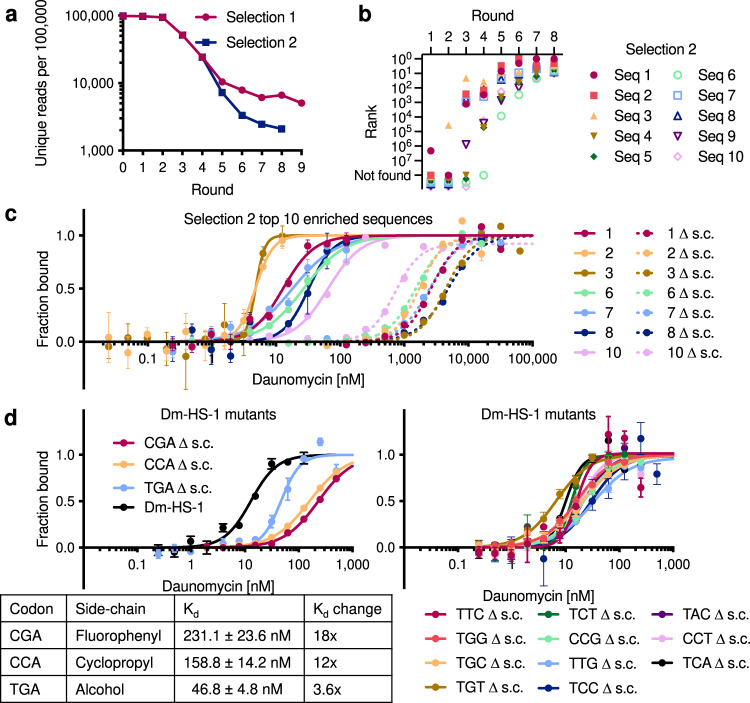
Increased selection stringency yields more potent daunomycin-binding HFNAPs. **a** Sequence convergence for selection 1 and selection 2. **b** The evolutionary trajectory of the top ten most enriched sequences in selection 2. **c** MST characterization of the binding affinity for the top ten most enriched sequences from selection 2. Dm-HS-4, 5, and 9 are omitted for clarity, as Dm-HS-1, 4, 5 and Dm-HS-3, 9 are each related by 1 nucleotide. 1 ∆ s.c. denotes the sequence (Dm-HS-1) without side-chains. Sequences were found to bind with *K*_d_ = 5–65 nM. Values and error bars reflect the mean and SEM for *n* = 3 independent replicates. Error bars for some values are too small to extend beyond the data point. **d** 14 Dm-HS-1 mutants were translated where a single building block is replaced with the corresponding building block lacking any side-chain. MST characterization of the binding affinity for these Dm-HS-1 mutants is shown. CGA ∆ s.c. denotes Dm-HS-1 without a side-chain for the CGA building block. The table shows individual side-chain removals that result in the largest increase in *K*_d_. The activity of the original Dm-HS-1 sequence is shown in black for comparison. Values and error bars reflect the mean and SEM for *n* = 3 independent replicates.

An analysis of the evolutionary trajectory of selection 1 informed our decision to restart the daunomycin-binding selection at round 4 with higher selection stringency. The top ten most enriched sequences from the start of the selection were already highly ranked as early as round 3 and subsequent rounds resulted in minimal changes to the overall ranking and distribution of top sequences (Fig. 2c). However, substantial enrichment occurred over these intermediate rounds, with the top ten most enriched sequences comprising 0.6%, 2.9%, and 10% of rounds 3, 4, and 5a, respectively. The most enriched sequence progressed similarly, representing 0.08%, 0.4%, and 1.4% of rounds 3, 4, and 5a, respectively. Overall, the intermediate rounds 3 to 5a exhibited a 5-fold sequence convergence that slowed thereafter (Fig. 3a). The overrepresentation of the top ten sequences in round 5, as well as the slowing of sequence convergence in subsequent rounds, eliminated the possibility of restarting the selection at round 5. We chose to restart the selection from the round 4 elution because of its favorable sequence diversity and also because the round 4 selection was the first in which we implemented target elution with free daunomycin, which in principle should bias for polymers with binding affinity for free daunomycin and remove those that require the linker or bead.

To increase requirements for target binding (*k*_on_) and dissociation (*k*_off_), we began by raising the selection temperature to 37 °C, as increased temperature generally increases *k*_off_ since dissociation is an entropically favored, TΔS > 0 process^36^. We also anticipated that elevated temperatures could denature marginally stable HFNAPs and thus enrich sequences that adopt more stable conformations. We further increased stringency by introducing multiple elution steps to our protocol. The original selection used a single target elution step of 30 minutes, eluting ≥95% of HFNAPs with a target-bound half-life ≤7 minutes and only partially eluting HFNAPs with longer half-lives. The inverse correlation between binding half-life and *k*_off_ suggests that this approach may result in selection survivors dominated by modest-affinity binders with fast off-rates rather than with strong binders with slow off-rates. To improve selection outcomes by selectively enriching slow off-rate HFNAPs, we implemented an iterated target elution step in which initial 1-minute and 10-minute elutions with 1 mM daunomycin allow weak binders with fast off-rates to elute and be discarded. The final 60-minute elution captures slower off-rate binders to advance into subsequent rounds of selection (Fig. 2a). We anticipated that the combined changes to increase selection stringency would increase the probability of identifying high-affinity HFNAPs and may even select against modest-affinity binders.

### High-stringency selection improves HFNAP binding affinity

After four additional rounds of high-stringency selection (Fig. 2a, selection 2, rounds 4 and 5b-8b), we observed accelerated sequence convergence, consistent with increased selection stringency (Fig. 3a). The increased selection stringency resulted in selection 2 enrichment values between round 4 and round 8b being greater than selection 1 enrichment values between round 4 and round 9a. The top ten most enriched sequences in selection 1 represented 2% of round 4 and 57% of round 9a, while the top ten most enriched sequences in selection 2 represented 0.3% of round 4 and 78% of round 8b, a 9-fold increase in the pooled enrichment (Supplementary Fig. 2). A comparison of the evolutionary trajectories of the top ten sequences in the two selection conditions revealed faster and larger ranking changes for the top sequences in selection 2 (Fig. 3b) compared to selection 1 (Fig. 2c). Increased selection stringencies also resulted in strong side-chain preferences among surviving sequences that favored the polar allylamine and alcohol side-chains (Supplementary Fig. 3), which were not observed in selection 1 (Supplementary Fig. 4). Likewise, we observed decreases in the side-chain frequency of the hydrophobic isopentyl, cyclopentyl, and fluorophenyl side-chains over the course of the selection. These trends contrast sharply with the outcomes of our previous HFNAP selections for binding target proteins, which strongly enriched for hydrophobic side-chains^16,23^. Finally, 676 of the top 1000 most enriched sequences at the end of selection 1 were found to de-enrich in selection 2 (Supplementary Fig. 5), demonstrating the effectiveness of high-stringency selection conditions in removing weak binders and providing further evidence that selection 1 resulted in modest daunomycin affinity. Collectively, these results suggest that the more stringent selection conditions in selection 2 increased enrichment efficiency, enabling identification of rare sequences.

To directly test whether increased selection stringency resulted in the isolation of HFNAPs with improved binding affinity, we used MST to characterize the binding affinities of the top ten most enriched sequences at the end of selection 2. These HFNAPs demonstrated binding affinities between *K*_d_ = 5–65 nM, representing a ~15-200-fold improvement over HFNAPs emerging from selection 1 (Fig. 3c and Supplementary Fig. 6). An independent binding assay using gel filtration of radiolabeled daunomycin corroborated this greatly improved binding affinity compared with the most highly enriched selection 1 HFNAPs (Supplementary Fig. 7). The same HFNAP sequences without the side-chains were also assayed for binding affinity, and showed ~10-1000-fold lower daunomycin affinity (Fig. 3c and Supplementary Fig. 6), indicating that the HFNAP side-chains play important roles in daunomycin binding.

To further characterize specific structure-activity relationships between the HFNAPs surviving selection and their side-chains, we synthesized 14 variants of Dm-HS-1, the most highly enriched sequence at the end of selection 2, round 8b, in which we replaced each building block with the corresponding DNA trinucleotide lacking any side-chain (Fig. 3d) (one building block, TGC, was present twice in Dm-HS-1). We found that two side-chains play a critical role in binding affinity. Removal of the fluorophenyl side-chain from the CGA building block at position 1 or removal of the cyclopropyl side-chain from the CCA building block at position 9 resulted in >10-fold loss in binding affinity. Additionally, loss of the alcohol side-chain from the TGA building block at position 6 decreased binding affinity by 3-fold. Removal of each of the remaining sidechains individually did not substantially affect binding affinity (Fig. 3d and Supplementary Table 2). Together, the results establish that increased selection stringency enriched rare HFNAPs with improved daunomycin-binding affinities that are strongly dependent on the presence of specific side-chains.

### Generative machine learning model methodology

Identifying active biopolymers beyond those closely related to current variants is a critical step towards mapping the global fitness landscape, and is a primary challenge of biopolymer evolution efforts^16^. Additional active variants can be found through increased experimental sampling, which results in more comprehensive sequence space search but is time- and labor-intensive. While multiple, parallel selections against the same target are likely to yield a more diverse set of active variants, each of these selections remains limited by the same sequencing constraints and sequence convergence inherent to in vitro selections. Recently, generative machine learning models have risen to prominence for their ability to generate entirely new text^37^, music^38^, or faces^39^ based on training examples. A generative machine learning model trained on HFNAP in vitro selection data could, in principle, learn the HFNAP fitness landscape for daunomycin binding. The resulting model could then directly generate many high-activity HFNAPs, in theory including those with sequence diversity approaching that of random sequences, obviating the need for increased sampling or search depth.

**Fig. 4:**
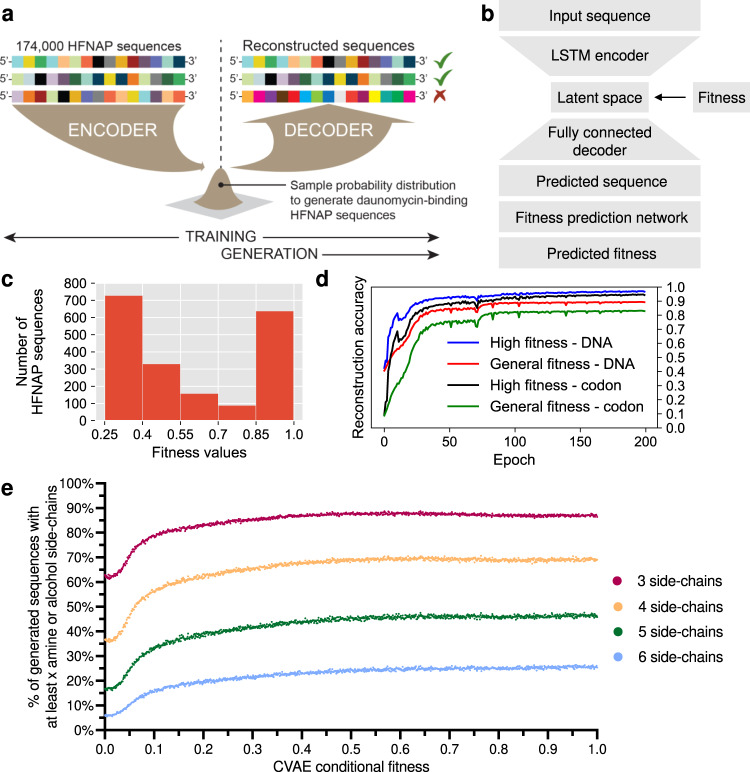
A machine learning model for generating HFNAPs with daunomycin binding affinity. **a** Overview of VAE structure, training and sequence generation. Sequence inputs are encoded into a probability distribution. A sample of the probability distribution is given to the decoder, which must reconstruct the original input sequence. Failure to accurately reconstruct the sequence results in penalties, which are used to optimize the parameters of the encoder, decoder, and probability distribution to improve reconstruction accuracy. After training, the probability distribution is then sampled from and given to the decoder to generate new HFNAP sequences. **b** The CVAE architecture used in this study. Conditional VAEs introduce an additional, conditional variable, which enables the user to specify a specific condition or state. Here, we introduce sequence fitness, a value between 0 and 1 that represents the anticipated binding affinity for daunomycin based on the sequence’s observed enrichment between round 4 and round 8b of selection 2, as the conditional variable (Methods). During training, the fitness condition becomes associated with sequence identity and the latent distribution. Subsequently, the fitness condition enables the user to specify that the trained CVAE generates HFNAPs with desirable binding affinities. **c** Distribution of fitness values assigned to training sequences that were greater than 0.25. **d** Reconstruction accuracy was used to assess training progress. The graph shows the reconstruction accuracies of the final, trained CVAE. Accuracy was calculated either by calculating the fraction of DNA bases that were correct, or the fraction of codons that were correct. **e** Percent of generated sequences with at least 3–6 alcohol or amine side-chains. Each point along the *x*-axis represents 10,000 CVAE-generated sequences that were generated with that given fitness value as the condition.

To generate HFNAP sequences with daunomycin affinity, we used a variational autoencoder (VAE), a type of generative model commonly used to make predictions with biological sequence data^40,41^. VAEs attempt to model the observed data, HFNAP sequences, as a function of a latent or hidden variable (using Bayes’ Theorem). The VAE model architecture begins with an encoder neural network that compresses the input sequence into a lower-dimensional space called the latent space. This dimensionality reduction from the input space to the latent space is critical because it forces the network to embed the latent space with only the information required for successful sequence reconstruction. The decoder network must then reconstruct the same input sequence by taking independent samples from this lower-dimensional representation, the latent probability distribution. During model training, the parameter weights for the two networks and the latent distribution are jointly optimized such that sequences can be accurately embedded into and reconstructed from the latent probability distribution. A visual example is shown in Fig. 4a, in which correct sequence reconstruction results in minimal changes to the model, while incorrect sequence reconstruction results in larger modification of the model to minimize the magnitude of the error. The trained model can then generate new HFNAP sequences by sampling from the latent probability distribution and using the generative decoder to produce new sequences (Fig. 4a).

A trained VAE model could therefore generate HFNAP sequences with a broad range of affinities for daunomycin. In order to direct the model to generate only the most active sequences, however, the model requires data labels that indicate whether sequences are active or inactive. We thus moved from an unsupervised model (without labels), the VAE, to a semi-supervised model (with labels), known as a conditional variational autoencoder (CVAE)^42^ (Fig. 4b). To label the in vitro selection data, we defined a fitness value between 0 and 1 to represent an HFNAP’s anticipated binding affinity for daunomycin based on the sequence’s observed enrichment between round 4 and round 8b of selection 2 (Methods, Fig. 4c, Supplementary Fig. 8). The majority of the fitnesses are near 0, with ~2000 sequences with fitness > 0.25. The latent distribution and fitness value are provided as inputs to the decoder, which first uses the information to reconstruct the sequence (Fig. 4b). The reconstructed sequence is then used as the input to an additional network, which predicts the fitness value given the sequence information. Because fitness prediction and sequence reconstruction are jointly optimized as parts of the decoder, sequence information becomes conditioned on fitness values, which can associate fitness values with regions of sequence and latent space. Successful conditioning will enable the CVAE to generate novel HFNAP sequences with high predicted daunomycin binding affinity.

### Training a CVAE on HFNAP in vitro selection data

We performed extensive architecture search to arrive at a CVAE capable of successful sequence reconstruction and fitness prediction. The CVAEs were trained on the 172,545 HFNAP sequences that were identified between round 4 and round 8b of selection 2. We used standard 80:20 train:test splits, in which we randomly withheld 20% of the data from training (Supplementary Fig. 8), then used the withheld sequences to test the performance of the model on unseen data. Initial progress on model training seemed promising, with >90% DNA reconstruction accuracy on the test set, with reconstruction accuracy defined as the percentage of accurate DNA backbone bases when comparing the correct HFNAP sequence to the HFNAP sequence predicted by the model (Supplementary Fig. 9). Since only 1% of the sequences were likely to have high-affinity, represented by the ~2000 sequences with fitness > 0.25 (Fig. 4c), we were concerned the model may not be accurate for high-affinity sequences and might overemphasize inactive or modest affinity sequences.

To observe the model’s performance on high-affinity sequences, we added an additional held-out high-fitness test set. This decision splits the data into three groups: the training set (80%), the general-fitness test set (19.9%), and the high-fitness test set (0.1%). The 256 high-fitness test set sequences included sequences related to Dm-HS-1-10, giving us confidence that model performance on these sequences would be indicative of model performance on high-affinity sequences. While this additional test set enabled us to specifically monitor model performance on high-affinity sequences, it decreased the number of sequences with high-fitness values (>0.85) available for model training by nearly half. These sequences were chosen based on having high round 4 to round 8b enrichment values in selection 2 (see Methods). Together, the general-fitness test set, comprised mostly of low fitness values, and the high-fitness test set, enabled us to more accurately observe changes to the model’s sequence reconstruction and fitness prediction performance in response to CVAE architecture modifications. After extensive architecture search, we achieved >95% DNA reconstruction accuracy on the high-fitness test set and, importantly, 89% DNA reconstruction accuracy on the general-fitness test set when comparing the input DNA backbone sequences and reconstructed DNA backbone sequences (Fig. 4d).

Model performance on both test sets is important. Supplementary Figure 10 shows the sequence-converged nature of the high-fitness test sequences compared to the general-fitness test sequences. If the model had only achieved high accuracy on the sequence-converged, 256-member high-fitness test set and low accuracy on the general-fitness test set, the trained CVAE might only generate sequences similar to those in the high-fitness test set. Conversely, high accuracy on the general-fitness test set and low accuracy on the high-fitness test set would likely translate to a failure to generate high-affinity sequences. Because the scarcity of active sequences in the large sequence space (~10^22^) presents the greatest challenge towards predicting high-affinity sequences, we have focused thus far on improving the model’s sequence reconstruction accuracy. The model’s high reconstruction accuracy for both test sets suggests that it may have learned the HFNAP fitness landscape for daunomycin binding.

We assessed the quality of our fitness model by comparing its outputs to the fitness values used during conditioning. 97.5% of training set and general-fitness test set sequences were conditioned on fitness values ≤ 0.05 (Supplementary Fig. 8). As a result, the mean squared error (MSE) between the fitness condition and the model outputs for general test set sequences was 0.102 ± 2.61 × 10^−5^ (mean ± standard deviation), compared to 0.0863 ± 2.07 × 10^−5^ for the training set, which suggests high accuracy. For the 849 general-test sequences with fitness value > 0.05, the model also performed well with an MSE of 1.52 ± 2.73 × 10^−4^. The model performed similarly on the high-fitness test set, with an MSE of 6.39 ± 2.43 × 10^−5^. The encouraging performance of the trained model on sequence reconstruction and predicting the fitness condition despite strong regularization enabled us to move forward with further characterization of the model.

Our earlier analysis of the selection 2 results had indicated a strong preference for amine and alcohol side-chains among enriched sequences (Supplementary Fig. 3). Because HFNAP fitness is a function of the sequence, we expect that an accurate understanding of the fitness landscape should capture relationships such as this one. This preference for amine and alcohol side-chains can be further quantified in the selection data by looking at the proportion of sequences with at least 3-6 amine or alcohol sidechains as a function of our assigned fitness values (Supplementary Fig. 11). To observe whether our CVAE captures a similar relationship, we generated HFNAPs by sampling the latent distribution using the reference set, the top 3072 HFNAP sequences from the training set with the highest fitness values. This data pre-processing step allowed us to discard poor-performing HFNAP sequences and focus on regions of latent space from the best-performing sequences. Sampling from this modified latent distribution and using the full range of fitness values to generate ten million new HFNAP sequences, we found that the model captured both the general frequencies and relationship between fitness and the number of amine or alcohol side-chains that was found in the training data (Fig. 4e and Supplementary Fig. 11). Interestingly, we found that the amine and alcohol side-chain proportion remained steady, even when conditioned on extended fitness values of 1.05-1.10 (Supplementary Fig. 12). While capping fitness values at 1 may ultimately limit the affinity of CVAE-generated HFNAPs, we note that conditioning on limited increases in fitness values exceeding 1 may be tolerated by the CVAE. These analyses indicated that the CVAE had learned a relationship between sequence and fitness, and suggested that the CVAE could be used to generate novel sequences with activity. Thus, we proceeded with experimental characterization, to determine if the CVAE had developed a sufficient understanding of the HFNAP fitness landscape for daunomycin binding to generate high-affinity sequences.

### Generating HFNAPs with high predicted daunomycin affinity

Satisfied with the CVAE’s sequence reconstruction and fitness prediction performance on computational tasks, we next investigated whether the model could generate HFNAP sequences with experimentally measured binding affinity for daunomycin. We used the CVAE to generate 10,000 HFNAP sequences, specifying the desired fitness values in increments of 0.05 ranging from 0.5 to 0.95 and sampling from the reference set latent distribution. The result was 1000 sequences at each fitness value: 0.5, 0.55, … 0.95, constituting 10,000 CVAE-generated HFNAP sequences for experimental validation.

To choose sequences for low-throughput binding affinity characterization of individual HFNAPs, we identified groups of generated sequences with higher levels of sequence similarity. Although the CVAE sampled randomly from the latent distribution, our previous experiment demonstrates that CVAE-generated sequences exhibit a strong preference for amine and alcohol side-chain encoding codons (Fig. 4e). Any sequence similarities found in the generated sequences could arise from this codon preference, or represent regions of increased model confidence that reflect important factors relevant for daunomycin binding. To determine sequence similarity among the CVAE-generated sequences, we calculated the pairwise Levenshtein distance (LD)^43,44^ between all 10,000 CVAE-generated HFNAP sequences (Supplementary Fig. 13). Levenshtein distance is the edit distance between two sequences using insertions, deletions, or substitutions; low Levenshtein distances indicate high sequence similarity.

**Fig. 5:**
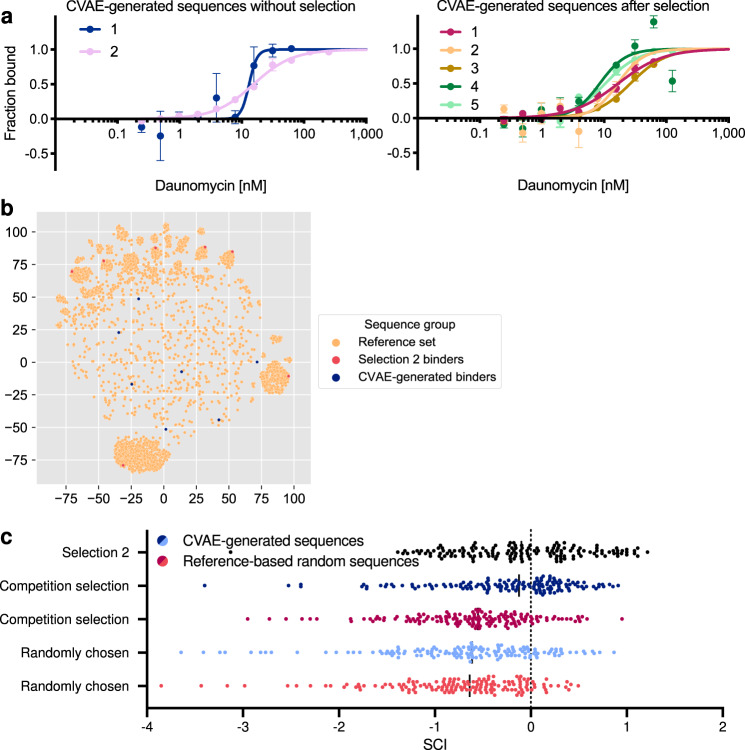
CVAE-generated sequences bind potently to daunomycin yet are unrelated to experimental sequences. **a** MST characterization of the binding affinity of CVAE-generated daunomycin-binding HFNAPs identified directly or with a single round of high-stringency selection. Sequences were found to bind with *K*_d_ = 13–15 nM and *K*_d_ = 9–26 nM respectively. Values and error bars reflect mean and SEM for *n* = 3 independent replicates. **b** UMAP projection (a non-linear method of dimensionality reduction) of reference set sequences, selection 2 daunomycin-binding HFNAP sequences, and CVAE-generated daunomycin-binding HFNAP sequences. 3072 reference-based random sequences and 3072 truly random sequences were included in the distance matrix and embedding but omitted from the final UMAP plot for clarity. Reference set sequences form clusters, indicating sequence similarity. Selection 2 daunomycin-binding HFNAPs, which are sequence related to the reference set, are found within these clusters. The selection 2 daunomycin-binding HFNAPs used were Dm-HS-1, 2, 3, 6, 7, 8, and 10. Dm-HS-4 and Dm-HS-5 were omitted for sequence similarity to Dm-HS-1, and Dm-HS-9 was omitted for sequence similarity to Dm-HS-3. **c** Aggregate SCI (structure conservation index) scores for CVAE-generated sequences, reference-based random sequences, and selection 2 sequences to Dm-HS-1-5 and Dm-HS-9. SCI ≈ 1 indicates complete conservation and SCI = 0 or less indicates lack of structure conservation. Many of the top 25 CVAE-generated sequences from the competition selection demonstrate structure conservation with Dm-HS-1-5 and Dm-HS-9, while structure conservation is rarer for reference-based random sequences. CVAE-generated sequences achieve increased numbers of positive SCI scores and larger SCI scores. Randomly chosen CVAE-generated sequences demonstrate increased structure conservation to Dm-HS sequences compared to the structure conservation between randomly chosen reference-based random sequences and Dm-HS sequences. Median values are indicated using solid black lines. Five sequences with SCI values < −4 are omitted for clarity. Two CVAE-generated sequences from the competition selection, two randomly chosen CVAE-generated sequences, and one reference-based random sequence from the competition selection.

Although most of the CVAE-generated sequences were distinct and characterized by distances similar to those of randomly sampled HFNAP sequences (Supplementary Fig. 14), 136 sequences exhibited pairwise LD ≤ 9. It is tempting to speculate that these rare clusters of increased sequence similarity reflect possible areas of model confidence. This subset of sequences characterized by pairwise LD ≤ 9 constituted about 1% of the generated HFNAPs, and can be further segmented into 128 single-pairs, six double-pairs, and two triple-pairs. A triple-pair is a generated HFNAP sequence that is related to three other generated HFNAPs by LD ≤ 9. Because triple-pairs may reflect local areas of model confidence, we chose the two triple-pairs (eight sequences total) for further characterization. MST characterization indicated that two of these sequences bound with *K*_d_ = 13–15 nM affinity (Fig. 5a and Supplementary Table 3).

### Experimental characterization of daunomycin affinity of CVAE-generated HFNAPs

To directly compare the binding affinity of CVAE-generated HFNAPs to the top experimentally derived sequences from selection 2, we competed them directly in an in vitro daunomycin-binding selection. DNA templates for the 10,000 CVAE-generated HFNAPs and the top 2000 HFNAPs from the training set with the highest fitness values (competition set) were translated as a mixture into HFNAPs, then subjected to a single round of high-stringency selection for daunomycin binding. Analysis of the HTS results indicated that of the 2433 sequences that had enriched during the selection, 48.6% were from the CVAE-generated sequences and 51.4% were from the competition set. The 1183 CVAE-generated sequences performed as well as, or better than, members of the competition set in the single round of high-stringency selection (Supplementary Fig. 15). We individually translated the five CVAE-generated HFNAP sequences with the highest enrichments for MST characterization and found them to bind daunomycin with 9–26 nM affinity (Fig. 5a and Supplementary Table 4). These model-generated, high-affinity binders exceed or match the daunomycin-binding affinity of experimentally isolated sequences from selection 1 and selection 2 that were subject to competition with ≥10^13^ variants. These results show further evidence that the trained CVAE can generate multiple validated HFNAPs with affinity for daunomycin comparable to that of HFNAPs that had survived multiple rounds of high-stringency selection.

While our primary goal was sequence generation of active HFNAPs with high daunomycin affinity, the competition selection also enabled us to explore how our conditioned fitness values relate to experimentally validated head-to-head enrichment. Assigning accurate fitness condition values for an entire in vitro selection is challenging, as round-to-round enrichments from HTS data are noisy proxies. Analysis of the previous head-to-head competition selection indicates that the correlation between the fitness condition and competition selection enrichment for the 2000 competition set sequences is 0.16 (Pearson *r*, *p* < 0.01, Supplementary Fig. 16). 143 of the 547 sequences assigned a fitness value of 0.999 did not enrich in this competition selection, which further supports the stringency of the selection and the noisiness inherent with enrichment values.

No correlation was found between the fitness condition and the competition selection enrichment for the 10,000 CVAE-generated sequences (Pearson *r* = −0.022, *p* = 0.028, Supplementary Fig. 16). We reasoned that this may be caused by our choice of fitness conditions > 0.5 when generating the HFNAPs. We therefore conducted an additional high-stringency, competition selection, using only CVAE-generated sequences, conditioned on fitness values ranging from 0.001 to 0.999 (see Methods). By competing 12,000 CVAE-generated HFNAPs without the addition of the competition set, we hoped to lower the internal selection stringency and observe how HFNAPs conditioned on low fitness value would perform. Including the competition set would likely prevent enrichment of the majority of the CVAE-generated HFNAPs, hindering us from exploring the relationship between fitness condition and enrichment. Initial analysis seemed to indicate that there was a negative correlation between conditioned fitness and enrichment (Pearson *r* = −0.627, *p* < 0.01; Spearman *r* = −0.766, *p* < 0.01; Supplementary Fig. 17). However, further analysis of the negative selection step also demonstrates the same trend, identifying increased enrichment of CVAE-generated HFNAPs conditioned on low fitness values for the negative selection condition (Pearson *r* = −0.642, *p* < 0.01; Spearman *r* = −0.708, *p* < 0.01; Supplementary Fig. 17). These results suggest that CVAE-generated HFNAPs conditioned on low fitness values may be nonspecific binders or only possess modest affinity.

In contrast, if we filter the 12,000 CVAE-generated HFNAPs for positive selection enrichments >1 or >2, we see that there is a positive correlation between conditioned fitness and enrichment (Pearson *r*_enrichment > 1_ = 0.198, *p* = 0.192; Spearman *r*_enrichment > 1_ = 0.449, *p* = 0.0020; Pearson *r*_enrichment > 2_ = 0.164, *p* = 0.283; Spearman *r*_enrichment > 2_ = 0.399, *p* = 0.0066; Supplementary Fig. 17). These results suggest that conditioning on high-fitness values produces HFNAPs with increased enrichment compared to conditioning on low fitness values, provided the generated HFNAP is active. Conditioning on high-fitness values is less likely to produce active HFNAPs, as demonstrated by the decreased percentage of sequences that enriched >1 or >2 (Supplementary Fig. 17). High-affinity HFNAPs are rare compared to modest-affinity HFNAPs, making the decreased frequency of generating active HFNAPs at high-fitness values consistent with in vitro selection principles. Together, these findings suggest a complex relationship between fitness and enrichment where conditioning on low fitness values produces nonspecific binders or modest affinity HFNAPs, and conditioning on high-fitness values produces higher affinity HFNAPs at a decreased rate.

To ensure that the enrichment of CVAE-generated HFNAPs was dependent on the model and not simply the observed amine and alcohol side-chain preference, we randomly sampled from the same building block frequency distribution as the reference set (the 3072 top-performing sequences from selection 2) to create the reference-based random set for a competition selection. DNA templates for the 10,000-member reference-based random set and the 2000-member competition set were translated as a mixture into HFNAPs, then subjected to a single round of high-stringency selection for daunomycin binding. HTS results revealed that no sequence in the reference-based random set enriched, with the highest enrichment value being 0.69, indicating widespread de-enrichment (Supplementary Fig. 18). We individually translated five sequences with the highest enrichment values for MST characterization and found none with notable daunomycin-binding activity (all had *K*_d_ ≥ 10 µM) (Supplementary Fig. 19). This result indicates that the building block distribution alone is not sufficient to confer daunomycin-binding activity, and that the affinity of the CVAE-generated sequences is indeed the result of the model having learned a useful representation of the daunomycin fitness landscape.

### CVAE-generated active HFNAP sequences are unrelated to reference set sequences

Understanding the global fitness landscape is critical to achieving multi-objective optimization for properties such as stability, binding affinity, and selectivity. To populate unknown regions of the global fitness landscape, CVAE-generated sequences should be unrelated to known sequences. Establishing the degree of dissimilarity between CVAE-generated active sequences and subsets of the training data also demonstrates whether the model has learned a fitness landscape that is distal to the local fitness landscape identified from selection 2. Here, we use Levenshtein distance and dimensionality reduction to determine if CVAE-generated HFNAP sequences are dissimilar to the reference set, establishing that the CVAE had learned sequence determinants of daunomycin activity beyond sequence similarity to reference set sequences, and also dissimilar to each other, establishing that the CVAE was capable of identifying diverse and distinct regions of active sequence space.

To determine whether the CVAE-generated daunomycin-binding HFNAPs are related to the reference set, we used the Levenshtein distance as our metric of sequence similarity and visualized sequences in two dimensions using dimensionality reduction by UMAP^45^. The UMAP plot visually demonstrates that the seven validated CVAE-generated HFNAP sequences are dissimilar from the reference set, as they remain separated from the clusters (Fig. 5b). In contrast, the seven validated selection 2 sequences are found within large clusters, indicating sequence similarity resulting from selection-induced sequence convergence. The distribution of Levenshtein distances to the reference set can be visualized directly, where we observe the seven CVAE-generated daunomycin-binding HFNAPs are separated from all 3072 reference set sequences by a Levenshtein distance of 15 or more (Supplementary Fig. 20, mean LD_reference set_ = 22.9 ± 1.21, *n* = 7) and from all training sequences by a Levenshtein distance of 12 or more (Supplementary Fig. 21, mean LD_training set_ = 23.20 ± 2.05, *n* = 7), indicating large dissimilarity. The distance distribution from CVAE-generated sequences to the reference set are similar to ones from seven reference-based random HFNAP sequences to the reference set (mean LD_reference set_ = 24.1 ± 0.78, *n* = 7) and from seven truly random HFNAP sequences to the reference set (1/32 building block distribution, mean LD_reference set_ = 24.9 ± 0.64, *n* = 7), with the closest sequence being 17 and 16 LD away, respectively (Supplementary Fig. 20). In contrast, 39% of the reference set is within a Levenshtein distance of only 1–2 to at least one of the seven validated selection 2 daunomycin-binding HFNAPs, an expected result of selection-induced sequence convergence (Supplementary Fig. 20, mean LD_reference set_ = 20.2 ± 1.90, *n* = 7). These data demonstrate that the CVAE-generated daunomycin-binding HFNAPs are unrelated to the reference set, and provides further support that the model has learned the daunomycin-binding fitness landscape distal to the local fitness landscape from selection 2.

Lack of broad sequence similarity to the reference set is maintained, even when considering all 10,000 CVAE-generated sequences. We began by calculating Levenshtein distances to the reference set from 10,000 training set sequences, 10,000 CVAE-generated sequences, 10,000 reference-based random sequences, and 10,000 truly random sequences (Supplementary Figs. 22-23). Distance distributions from the reference-based random sequences and truly random sequences to the reference set are representative of broad sequence dissimilarity that is derived from their random sampling. If the Levenshtein distances from the 10,000 CVAE-generated sequences to the reference set are similar to what is observed in the two random controls, then overall CVAE-generated sequence similarity to the reference set should be minimal. To assess similarity of the distance distributions, we calculated the Jensen-Shannon (JS) distance^46,47^, a metric of similarity between two probability distributions where low values reflect increased similarity. We find that CVAE-generated sequences are dissimilar to the reference set, as its Levenshtein distance distribution to the reference set is similar to those of the two random controls: reference-based random sequences (JS distance = 0.160) and truly random sequences (JS distance = 0.249). In contrast, the top 10,000 training set sequences are more similar to the reference set, as indicated by the presence of low Levenshtein distance values and the larger JS distances to the two random controls: reference-based random sequences (JS distance = 0.258) and truly random sequences (JS distance = 0.321). Additionally, the closest Levenshtein distances for the 10,000 reference-based random sequences and the 10,000 truly random sequences to the reference set are 11 and 12, respectively (Supplementary Fig. 22). We find that only 16 and 43 of the 10,000 CVAE-generated sequences exhibit Levenshtein distances to the reference set that are <11 or <12, respectively. The dissimilarity between CVAE-generated HFNAPs and the reference set indicates the model is likely generating HFNAPs based on an understanding of the broad fitness landscape, rather than relying on sequence similarity to the reference set.

### CVAE-generated active HFNAPs explore distinct regions of the global fitness landscape

To assess whether the validated CVAE-generated daunomycin-binding HFNAPs are derived from a single local fitness landscape as opposed to different areas of the global fitness landscape, we compared the pairwise Levenshtein distances within groups of HFNAPs. The seven validated CVAE-generated daunomycin-binding HFNAPs have a median pairwise distance of 23 LD. Because the distance distribution of Dm-HS-1-7 would be skewed by Dm-HS-4-5 (point mutants of Dm-HS-1), we chose to examine experimental sequences Dm-HS-1-3, Dm-HS-6-8, and Dm-HS-10 (Dm-HS-9 omitted for similar reasons), and found a median pairwise distance of 21 LD (Supplementary Fig. 24). To determine if there are significant differences in median pairwise distances between groups of sequences, we analyzed the pairwise distance distributions using permutational analysis of variance (PERMANOVA)^48^. PERMANOVA establishes the statistical significance of the larger median pairwise distances between the seven validated CVAE-generated HFNAPs (LD = 23) compared to the seven selection 2 HFNAPs (LD = 21), as indicated by larger pseudo F statistic values (pseudo *F* = 1.531, *p* = 0.0494) (Supplementary Fig. 24), which provides supporting evidence that validated CVAE-generated HFNAPs sample broader sequence space compared to the validated selection 2 HFNAPs. Furthermore, pairwise distances are also a measure of sequence diversity, and we find the sequence diversity of the seven CVAE-generated HFNAPs to be similar to that of the seven reference-based random sequences (pseudo *F* = 1.001, *p* = 0.485). In contrast, the selection 2 HFNAPs differ significantly from the reference-based random sequences (pseudo *F* = 1.674, *p* = 0.0234), although part of the effect may be attributed to heterogenous dispersions (*p* = 0.0458) (see Methods). These results suggest that the model produces active HFNAPs with sequence diversity exceeding what we obtained from in vitro selection. Extending the pairwise distance calculations to all 10,000 CVAE-generated HFNAPs, we observe similar relationships qualitatively (Supplementary Fig. 25). The pairwise distance distribution between the validated CVAE-generated daunomycin-binding HFNAPs exhibits sequence diversity exceeding that of experimentally derived sequences from in vitro selection, which further demonstrates that the CVAE is able to access distinct areas of the fitness landscape.

### CVAE-generated and in vitro selected HFNAPs share similar predicted secondary structures

To begin to understand the CVAE’s ability to generate active, daunomycin-binding HFNAPs despite the lack of sequence similarity with HFNAPs from the in vitro selection, we analyzed the predicted secondary structures of various HFNAP-DNA backbone sequences using the DNA parameter set in RNAstructure^49^. While structure prediction for HFNAPs is more difficult as the side-chains can influence HFNAP secondary structure and folding^16^, similar secondary structure elements have been observed in modified nucleic acid polymers^50^. As expected, given that Dm-HS-1 was highly enriched and the dominant family of sequences in our in vitro selection, several of the most enriched CVAE-generated HFNAPs from the competition selection (Dm-CCS variants) had predicted structures with qualitative structural similarities with the predicted structures of Dm-HS-1 (Supplementary Figs. 26-27). Using RNAdistance^51,52^, we calculated that Dm-CCS-3 structure 2 was 24 base-pair-edit distance away from the Dm-HS-1 MFE (minimum free energy) structure, and the Dm-CCS-5 MFE structure was 30 base-pair-edit distance away from Dm-HS-1 structure 3. These smaller base-pair-edit distances were relatively rare, as indicated by our expanded base-pair-edit distance analysis of the top 25 CVAE-generated sequences and reference-based random sequences from the competition selection, and 25 randomly chosen sequences from each of these sequence sets (Supplementary Fig. 28). These initial structural similarities between validated, daunomycin-binding CVAE-generated HFNAPs and Dm-HS-1 prompted us to fully characterize the extent of structural similarity between CVAE-generated and experimental sequences.

To determine if predicted structures were similar, we calculated the structure conservation index (SCI)^53^ (see Methods). SCI score has been used as the standard metric for structure conservation and structure similarity^54,55^, whereas base-pair-edit distance has been shown to be a less reliable metric, especially if sequences have limited sequence similarity^54^. SCI scores near or below 0 indicate lack of structural similarity and SCI ≈ 1 indicates complete structural conservation. We calculated the SCI scores from Dm-HS-1-10 to the top 25 CVAE-generated sequences from the competition selection, top 25 reference-based random sequences from the competition selection, 25 randomly chosen CVAE-generated sequences, 25 randomly chosen reference-based random sequences, and the top 25 most enriched selection 2 sequences. The aggregate results are shown in Supplementary Fig. 29. We observed that the CVAE-generated sequences have increased structural similarity with some (Dm-HS-1-5 and Dm-HS-9) but not all of the Dm-HS sequences, as indicated by the presence of increased number of structures with positive SCI scores than in the reference-based random sequences, along with the larger SCI scores (Fig. 5c, and Supplementary Figs. 30 and 31). Both the CVAE-generated sequences from the competition selection and randomly chosen sets demonstrate this increased structural similarity when compared to the reference-based random sequences, indicating that the structural similarities observed are a function of the CVAE sequence generation and not just a consequence of the selection process.

The selective structural similarity is likely a result of Dm-HS-1-3 dominating the high-fitness training data. Dm-HS-1-5 and Dm-HS-9 represent the three major families from selection 2, as Dm-HS-4-5 are point mutants of Dm-HS-1 and Dm-HS-9 is a point mutant of Dm-HS-3. Of the 1000 selection 2 sequences with the highest assigned fitness values (fitness > 0.4666), 42.9% are within LD 1–2 of Dm-HS-1-3. The observed increase in structural similarity between CVAE-generated sequences and these three major families is therefore likely a product of our specific training data. As a result, the overrepresentation of these related sequences as high-fitness sequences likely resulted in a strong contribution to the CVAE’s understanding of daunomycin binding.

To probe if Dm-HS-1-3 or any top Dm-HS sequence contributes to CVAE sequence prediction, we first analyzed our selection 2 HTS data using AptaTrace to identify potential motifs. AptaTrace is a computational method for identifying sequence-structure motifs based on motif-induced selection trends^56^. To determine if any of the potential motifs were selectively enriched in CVAE-generated sequences, we used Sequenceserver to run Basic Local Alignment Search Tool (BLAST) on custom BLAST databases created from our 10,000 CVAE-generated sequences and 10,000 reference-based random sequences^57,58^. We identified three motifs that were overrepresented in the 10,000 CVAE-generated sequences compared to the 10,000 reference-based random sequences (Supplementary Fig. 32). For a given *E*-value, the number of expected alignments that would occur based on chance given the number and length of sequences in the database, these motifs were identified in CVAE-generated sequences at least ≥2.66-fold more often than in reference-based random sequences, with the exception of motif 2 (*E*-value 0.18). These results suggest that the CVAE learned the importance of including motifs 1-3 for predicting sequences with high affinity.

To better understand the CVAE’s insertion of these motifs in predicted HFNAP sequences, we characterized motifs 1-3 in the CVAE-generated sequences by comparing motif positioning and the predicted structural similarity to the corresponding Dm-HS sequence. Motif 1, which can be found in Dm-HS-10, is positioned at the beginning of the HFNAP coding region in selection 2 round 8b sequences (Supplementary Fig. 33). When we compared where this motif was found in the 10,000 CVAE-generated sequences (12 instances) and 10,000 reference-based random sequences (10 instances), we observed that CVAE-generated sequences containing motif 1 had also positioned the motif at the beginning of the HFNAP coding region. Reference-based random sequences containing motif 1 were distributed throughout the coding region with a slight increase near the center of the coding region. A similar pattern is observed for motif 2, found in Dm-HS-5. Motif 2 is positioned at the end of the coding region in selection 2 round 8b sequences, which matches where the motif 2 was most commonly found in the 103 CVAE-generated sequences containing motif 2. Motif 3, found in Dm-HS-8, is centered in the HFNAP coding region of selection 2 round 8b sequences (position 16-27). While the 130 CVAE-generated sequences containing motif 3 achieve a similar centering, the exact positions differs, with the majority of CVAE-generated sequences incorporating motif 3 at position 25-36. The 26 reference-based random sequences incorporating motif 3 are again distributed throughout the coding region. Surprisingly, we were able to identify one CVAE-generated sequence that combines motif 2 and 3 with a 3 bp overlap (BLAST *E*-value 8.60 × 10^−5^). This 18 bp combined motif is not found in any training data sequence. Three sequences contain a 15 bp sequence that matches the combined motif (BLAST *E*-value 6.31 × 10^−2^), one of which is in the general-fitness test set and was not used for model training. While the sequence did not enrich in the competition selection, this finding demonstrates the ability of the CVAE to combine motifs. The positional matching of these motifs in CVAE-generated sequences is supporting evidence that the CVAE has identified specific motifs to incorporate when generating high-affinity HFNAPs.

We then calculated SCI scores to determine whether the top 50 CVAE-generated HFNAPs incorporating these motifs had increased structural similarity to the corresponding Dm-HS sequence. The top 50 CVAE-generated sequences containing motif 1 (by BLAST bitscore) were found with increased structural similarity to Dm-HS-10, compared to the top 50 reference-based random sequences containing motif 1, 50 randomly chosen CVAE-generated sequences, or 50 randomly chosen reference-based random sequences (Supplementary Fig. 34). SCI scores for motif 2 and 3 produced similar findings. These results are surprising given that incorporation of the 12-13mer motif sequence does not translate directly to structural similarity for the 81mer sequence. The combined evidence of CVAE-generated sequences containing motifs 1-3 exhibiting structural similarity to the corresponding Dm-HS sequences and positional matching of these motifs to selection 2 round 8b sequences further suggests the CVAE has learned to incorporate important motifs when generating HFNAPs.

Importantly, these structural similarities are present despite the lack of broad sequence similarity. Supplementary Figs. 30 and 31 plot the LD from Dm-HS-1-10 to 25 CVAE-generated sequences, reference-based random sequences, and selection 2 sequences. Here we observe that only selection 2 sequences exhibit low LD values, indicating sequence similarity. There is limited sequence similarity between Dm-HS-1-10 and the top 25 CVAE-generated sequences from the competition selection, the top 25 reference-based random sequences from the competition selection, the 25 randomly chosen CVAE-generated sequences, or the 25 randomly chosen reference-based random sequences. Collectively, these results suggest how structure similarity to the three major families from selection 2 is a partial contributor for CVAE model performance. By training on the sequence and fitness data, the CVAE may indirectly identify structural motifs that contribute to fitness, and uses these learned motif representations in at least some cases to generate sequences with high daunomycin-binding activity.

## Discussion

Insights into the global fitness landscape of proteins, nucleic acids and other functional sequence-defined polymers promote the accurate prediction of high-fitness sequences and a broader understanding of their binding interactions. While in vitro selections are an efficient method for exploring large areas of sequence space, selection-induced sequence convergence and limited sequencing depth constrain the characterization of fitness landscapes to the few regions containing sequences that survived multiple rounds of selection and that were successfully synthesized. While additional rounds of in vitro selection could, in principle, enable access to a larger area of sequence space, this solution is costly in terms of time and resources. In contrast to performing several rounds of in vitro selection, machine learning models can be trained to make accurate predictions about the sequence space, even when data is limited. These models could then expand the sequence diversity of known active sequences and enable a fuller characterization of the fitness landscape of functional sequence-defined polymers.

In the present study, we trained a generative machine learning model on HFNAP in vitro selection data in order to generate diverse HFNAPs with high binding affinity for the small-molecule daunomycin. By leveraging the chemical functionality of HFNAPs and improving selection conditions, we produced a dataset of 10^5^ experimental sequences. We assigned fitness values to the sequences based on their enrichment during in vitro selection and trained a conditional variational autoencoder (CVAE), a type of generative machine learning model. Although active sequences were far outnumbered by inactive sequences, the CVAE learned the joint probability distribution between sequence identity and binding affinity and then directly generated HFNAPs with potent daunomycin-binding affinities. Importantly, the CVAE-generated sequences were unrelated in sequence to experimentally identified active HFNAPs, and a competition selection demonstrated that select CVAE-generated sequences performed as well as or better than sequences emerging from multiple rounds of high-stringency in vitro selection. While daunomycin-binding HFNAPs from the in vitro selection and CVAE-generated sequences fold into a variety of different predicted structures, secondary structure analysis suggests that the CVAE’s understanding of the daunomycin-binding fitness landscape is supported in part by its ability to generate secondary structures resembling those of Dm-HS-1-3. The lack of sequence similarity to Dm-HS-1-3, despite the fact that these three sequence families were major components of the high-fitness sequences, highlights the sequence diversity of CVAE-generated sequences. As a result, CVAE sequence generation can counteract the sequence convergence typical of traditional in vitro selections, while maintaining high on-target activity. Our method expands the sequence diversity of sequences, thereby increasing the likelihood of identifying candidate sequences with diverse properties including those that may not have been explicitly selected, but which are desirable. For example, increasing levels of backbone and 2'-position modifications usually reduces on-target activity^59,60^, but having diverse sequences increases the probability of identifying sequences amenable to chemical modification. Collectively, these results demonstrate that generative machine learning models can be used to generate active polymer sequences and reduce the number of in vitro selections required to discover diverse and highly active variants.

Researchers in the life sciences are increasingly recognizing the power of machine learning models to make predictions about biological data^61,62^ and to improve protein function by efficiently exploring fitness landscapes^24,27^. Notably, Biswas and colleagues have made impressive progress in successfully limiting the amount of data a model needs to be trained on to achieve predictive power^27^. Pre-training models on the ~24 million UniRef50 amino-acid sequences allows them to learn fundamental features necessary for protein function. The resulting models can then be fine-tuned using <100 functional variants of the desired protein, minimizing the amount of labeled data that needs to be individually characterized. Additionally, Stokes and co-workers showed that message passing neural networks trained on small molecules can guide drug discovery by making predictions on new, unrelated compounds^61^. Both of these examples illustrate the importance of training on useful datasets to achieve sufficient predictive power. We anticipate that advances in model construction and training will only continue to improve researchers’ abilities to generate useful predictions that can outperform training examples.

More broadly, this work demonstrates that integrating in vitro selection with machine learning can help researchers explore a much larger swath of a polymer’s total sequence space than would be possible from in vitro selections alone. The improved sequence and structural diversity that can result from such an exploration increases the likelihood of accessing polymers with properties suitable for biotechnological or therapeutic applications, such as amenability for post-selection chemical modifications that can have varying sequence- and structure-dependent effects on binding affinity^63^. Traditional in vitro selections use only a small fraction of information-rich HTS data to identify enriched clones and putative consensus sequences. In contrast, the integrated in vitro selection and machine learning approach developed here makes better use of this information and may enable the discovery of active sequences that survive difficult selection criteria with few solutions. Our approach may also facilitate the generation of synthetic polymer catalysts with activities not known among previously described biopolymers.

## Methods

### General methods

Oligonucleotides were purchased from IDT and are listed in the Supplementary Information. Building blocks were synthesized as described previously^16,21^. Building blocks were synthesized either directly from phosphoramidites (Glen Research) or via coupling of an amine to the NHS ester containing phosphoramidite as described previously^16,21^. T3 DNA ligase, ATP, and T4 RNA ligase buffer were purchased from New England BioLabs. MyOne Streptavidin C1 magnetic beads (Life Technologies) were used in immobilization. MST samples were prepared using HBS-P + buffer (GE Healthcare Life Sciences).

### Synthesis of HFNAP libraries by DNA-templated translation

DNA template libraries (125 pmol), initiation primer (188 pmol), termination primer (188 pmol), monomer mix (1.25 nmole/monomer), and 13 µL of water were mixed and added to a PCR strip. Strips were then incubated at the following temperatures using a thermal cycler: 95 °C for 10 s, 65 °C for 4 min, followed by a 0.1 °C per 10 s ramp to 4 °C. 6.25 µL of ATP and 6.25 µL of T3 DNA ligase were then added at 4 °C, and then incubated for 16 h at 4 °C.

250 µL of streptavidin beads were then prepared by washing in B&W buffer according to manufacturer protocol. The beads were then changed into 125 µL of 2× B&W (20 mM Tris-HCl, 2 M NaCl, 2 mM EDTA, pH 7.5). The translation reaction was then combined with the streptavidin beads and mixed for 1 h on a rotary mixer. The supernatant was discarded, and beads were washed thrice to remove nonspecific binders. The HFNAP strands were then selectively eluted from the biotinylated template using two 5-min washes with 250 µL of freshly prepared 20 mM NaOH. The combined elution was mixed with 5 volumes of column-binding buffer (2:3 sat. aq. guanidine•HCl:isopropanol, 1% v/v 3 M NaOAc, 0.1%  pH indicator). The combined sample was then column purified using a QiaPrep 2.0 column and eluted with 52 µL of EB (Qiagen). A 1-µL aliquot was saved as the pre-selection library.

Subsequent rounds of translation require streptavidin bead immobilization of double-stranded DNA templates and 20 mM NaOH washes to obtain ssDNA templates for translation. Beads are then washed twice with T4 RNA ligase buffer to neutralize any remaining base before proceeding with synthesis and translation as usual. These bead immobilized templates are then used in lieu of in solution templates.

### Synthesis of individual HFNAPs by DNA-templated translation

Synthesis of individual HFNAPs follows a similar protocol as library synthesis. The major difference is use of 10 equivalents of the building blocks for each HFNAP as opposed to a master mix. Following translation, HFNAP isolation and elution, and column cleanup, the sample is then PAGE purified on a denaturing 10% TBE-Urea gel (Criterion, Bio-Rad). The band is visualized by UV shadowing, where DNA bands were identified by the shadow cast by DNA onto a TLC plate with F254 indicator when the gel was illuminated by a UV lamp. The bands were excised from the gel and extruded through stacked microcentrifuge tubes (0.2 mL, 0.5 mL, 1.5 mL), with 17 gauge and 27 gauge holes made in the first two tubes respectively. The resulting material was then resuspended in 400 µL of Crush Soak (1× TE, 200 mM NaCl), and then shaken overnight at 37 °C.

The gel pieces were then removed using 0.22 µm PVDF centrifugal filters (Millipore). The remaining liquid was then combined with 5× equivalents of column-binding buffer, DNA column purified, and eluted with water.

### Synthesis of positive and negative selection targets

Biotinylated daunomycin was synthesized by reacting EZ-Link Sulfo-NHS-SS-Biotin (Thermo Scientific) with daunomycin (Selleckchem)^33^. The product was HPLC purified, and concentration determined by A260.
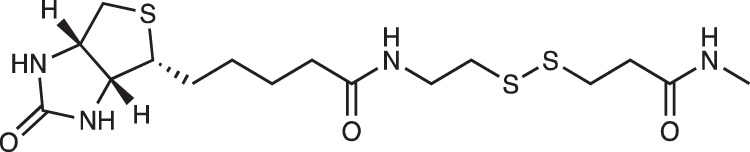


The negative selection target, Me-SS-Biotin, was prepared according to the following procedure. The reaction was conducted under N_2_ atmosphere. EZ-Link™ Sulfo-NHS-SS-Biotin (ThermoFisher Scientific) (31.0 mg, 0.0511 mmol) was weighed directly into an oven-dried vial sealed with a Teflon septum and containing a magnetic stir bar. The vial was cooled to 0 °C, and 10 mL of 2 M methylamine in methanol was added to the vial dropwise. The reaction mixture was allowed to warm to room temperature slowly and stirred for 24 h. After this time, the solvent and excess methylamine were removed *in vacuo*. The crude reaction mixture was dissolved in 30/70 acetonitrile:water and purified by reverse phase HPLC (0/100 to 30/70 of acetonitrile/water with 0.1% trifluoroacetic acid). The product was lyophilized overnight before further use.

^**1**^**H NMR** (400 MHz, CD_3_OD) δ 4.521(ddd, *J* = 8, 4.8, 1.2 Hz, 1H), 4.336 (dd, *J* = 8, 4.4 Hz, 1H), 3.509 (*t*, *J* = 6.8 Hz, 2H), 3.239 (ddd, *J* = 10.4, 4.4, 4 Hz, 1H), 2.979 (*t*, *J* = 8 Hz, 2H), 2.960 (dd, *J* = 14, 4.8 Hz, 1H), 2.856 (*t*, *J* = 5.2 Hz, 2H), 2.755 (*s*, 3H), 2.738 (*d*, *J* = 14 Hz, 1H), 2.615 (*t*, *J* = 7.2 Hz, 2H), 2.250 (*t*, *J* = 7.6 Hz, 2H), 1.695 (*m*, 4H), 1.479 (*p*, *J* = 7.6 Hz, 2H).

^**13**^**C{**^**1**^**H} NMR** (151 MHz, CD_3_OD) δ 177.09, 175.15, 166.99, 64.24, 62.50, 57.85, 41.90, 40.37, 39.41, 37.59, 37.43, 36.05, 30.60, 30.33, 27.67, 27.24.

**MS** (ESI): *m/z* for C_16_H_28_N_4_O_3_S_3_ [M + TFA-H]^−1^ calcd.: 533.12, found: 533.14 (see Supplementary Note 2).

### Daunomycin affinity selection

Daunomycin-linked beads and negative selection beads were prepared prior to the start of each selection round. Streptavidin-linked beads were washed with B&W according to manufacturer protocol, then incubated with biotinylated daunomycin or the negative selection linker (see Methods) for 1 h. The beads were then washed in selection buffer (20 mM Tris-HCl (pH 7.4), 140 mM NaCl, 5 mM KCl, 1 mM MgCl_2_, 1 mM CaCl_2_, 0.05% [v/v] Tween 20) thrice, and then resuspended in 2× selection buffer.

The selection began with incubation of column purified HFNAP libraries with immobilized daunomycin on streptavidin-linked beads. The selection was placed on a rotary mixer for 1 h. The flow-through was then collected, the beads washed 3× using selection buffer, and finally putative binders were eluted according to the scheme in Fig. 2a. When eluting with 1 mM daunomycin, the beads were washed twice with selection buffer after the initial incubation with daunomycin. The combined material constitutes the eluted material. The eluted material was then mixed with 5× equivalents of column-binding buffer, DNA column purified and eluted with water.

Selections started with a negative selection after round 4. The column-purified HFNAP libraries were mixed with negative selection beads on a rotary mixer for 30 min. The flow-through was then collected, and mixed with positive selection, daunomycin-linked beads for 1 h.

A 1-µL aliquot of each sample was analyzed by qPCR using Q5 Hot Start High Fidelity 2× Master Mix (New England BioLabs) and 0.5× SYBR Green I (Life Technologies). The qPCR cycle number was used to determine the number of cycles of amplification for the eluted material. The eluted material was amplified by PCR using Q5 Hot Start High Fidelity 2× Master Mix (New England BioLabs), and a primer set in which one of the primers was biotinylated. The resulting PCR reaction was then DNA column-purified using 5× equivalents of column-binding buffer, and eluted with water.

The eluted material was then gel purified in a 10% TBE native PAGE gel (Criterion, Bio-Rad). The gel was visualized by UV shadowing, where DNA bands were identified by the shadow cast by DNA onto a TLC plate with F254 in which when the gel was illuminated by a UV lamp. The bands were excised from the gel and extruded through stacked microcentrifuge tubes (0.2 mL, 0.5 mL, 1.5 mL), with 17-gauge and 27-gauge holes made in the first two tubes respectively. The resulting material was then resuspended in 400 µL of Crush Soak (1× TE, 200 mM NaCl), and then shaken overnight at 37 °C.

The gel pieces were then removed using 0.22 µm PVDF centrifugal filters (Millipore). The remaining liquid was then combined with 5× equivalents of column-binding buffer, DNA column purified, and eluted with EB. The absorbance was then measured to determine the concentration of the eluent, for subsequent rounds of translation.

### High-stringency selection scheme

The two main differences for the high-stringency selection scheme were the selection temperature and the elution conditions. The positive and negative selections were both performed at 37 °C, with an initial 2-min incubation in a thermocycler to rapidly raise the temperature of the selection to 37 °C prior to incubation on a rotary mixer in a 37 °C warm room. The elution condition was then modified to contain multiple washes with 1 mM daunomycin, followed by 2 washes with selection buffer. The incubation times and the elution that was carried forward to the next round of selection are shown in Fig. 2a.

### HTS analysis of libraries

Samples to be analyzed were amplified by PCR using Q5 Hot Start High-Fidelity 2× Master Mix to install Illumina sequencing adapters, with amplification cycle number determined by qPCR. Samples were then DNA column purified, followed by an additional PCR round to install Illumina barcodes. The resulting samples were then combined, DNA column purified, and the concentration was determined by Kapa Library Quantification Kit (Kapa Biosystems). Fastq files were then parsed using AptaSuite, setting a strict cutoff for full-length sequences^64^. The parsed sequences were exported through AptaSuite in Fastq format, and subsequently converted into csv’s for downstream analysis.

### Microscale Thermophoresis (MST)

MST analysis was conducted using a Monolith NT.Automated (Nanotemper). Analyses were conducted at 25 °C in HBS-P + buffer (Cytiva/GE Healthcare Life Sciences) with additional 5 mM KCl, 1 mM MgCl_2_, and 1 mM CaCl_2_. Sequences were translated using a Cy5 tag and PEG18 spacer, for use in tracking the HFNAP. Generally, the laser excitation energy was set to 10 % to minimize photobleaching, and the IR laser power was set to high for all readings.

Dilution series for each replicate was made separately, using serial dilutions from the highest concentration to the lowest concentration. The amount of labeled HFNAP that was used was subsaturating, as determined by fluorescence intensity measured against a known standard. Measurements were made in triplicate. Data was processed and fitted using standard Nanotemper software (MO.Affinity) and exported to Prism for plotting.

### Gel filtration

Gel filtration was conducted using [^3^H]-daunomycin, from Perkin-Elmer or American Radiolabeled Chemicals^65^. Analyses were conducted at 25 °C in selection buffer (20 mM Tris-HCl (pH 7.4), 140 mM NaCl, 5 mM KCl, 1 mM MgCl_2_, 1 mM CaCl_2_, 0.05% [v/v] Tween 20). 25 µL of a 40 nM solution of the HFNAP in selection buffer was mixed 1:1 with 25 µL of serial dilutions of radiolabeled daunomycin. The initial daunomycin mixture was made by mixing [^3^H]-daunomycin with non-radiolabeled daunomycin, followed by 1:1 serial dilution with selection buffer. Samples were incubated in the dark at 25 °C for at least 30 min. During incubation, Centri-Sep gel filtration column (Princeton Separations catalog number CS-901) were prepared according to manufacturer instructions by reconstituting with selection buffer. The 50 µL reaction was loaded onto the prepared Centri-Sep columns, followed by centrifugation according to manufacturer instructions. The resulting flow-through was pipetted into 5 mL of Ultima Gold (Perkin-Elmer) in a scintillation vial, vortexed, and counted using a Tri-Carb 2910 TR liquid scintillation counter. The process was conducted in parallel with an unrelated sequence from a different selection against a different target (gel filtration control sequence). Fraction bound by Dm-HS-1 was calculated by taking the difference between the amount of [^3^H]-daunomcyin bound by Dm-HS-1 and the amount bound by the control sequence.

### PERMANOVA

Permutational multivariate analysis of variance was conducted using the scikit-bio package. A symmetric matrix of precomputed Levenshtein distances between two groups of seven sequences was used as the distance matrix. Comparisons were first tested for heterogenous dispersions using the permdisp function (permutations = 1000, test = centroid), repeated ten times, and taking the average *p*-value. PERMANOVA was then applied using the permanova function, (permutations = 1000) 1000 times, and the average *p*-value was reported.

### SCI scores

Structure conservation index (SCI) is defined by the equation:

$${{{{{{{\rm{SCI}}}}}}}}={E}_{{cons}}/{\bar{E}}_{{single}}$$

Where ${E}_{{cons}}$ is the consensus sequence MFE, and ${\bar{E}}_{{single}}$ is the average MFE of individual sequences. SCI scores were calculated by first conducting a structural alignment of sequence pairs using multilign^66^, a package in RNAstructure^49^. The aligned sequences were then passed through RNAalifold to calculate ${E}_{{cons}}$^55,67–69^. The individual MFEs were then calculated using RNAeval^54^, using the individually folded, predicted structures from RNAstructure.

### Base-pair-edit distance

Base-pair-edit distance was calculated between sequences by converting predicted structures from CT files to dot-bracket notation using RNAstructure. Sequences in dot-bracket notation were then compared using RNAdistance^51,52^.

### Motif position mapping

Using AptaTrace^56^ sequence-structural motifs from selection 2 were identified and aligned against the 10,000 CVAE-generated sequences and the 10,000 reference-based random sequences using BLAST. The aggregate aligned positions are plotted using a cutoff bitscore (80% of the highest value found in the CVAE-generated sequences).

### Pearson correlation and spearman correlation

Correlations were calculated using GraphPad Prism version 9 for MacOS, GraphPad Software, San Diego, California USA, www.graphpad.com. Two-tailed *p*-values were reported.

## Machine learning model

To gain further insight into the fitness landscape of daunomycin binding, we applied deep learning techniques to model the HFNAP sequences from selection 2.

### Training dataset

The training dataset was constructed from HTS analysis of the high-stringency selection, round 4 through round 8b. To begin, the five rounds of selection 2 were subject to HTS, resulting in sequence abundances (counts) for each round of the selection. To account for differences in sequencing read depth for each of these rounds, and to normalize the number of reads per sample, we bootstrapped the sequence abundances. Data for each round was randomly sampled 1 million times with replacement, and this process was iterated 1000 times to calculate bootstrapped sequence abundances. The rounds were then combined to create the training dataset, where each row is a single, unique sequence, and each column is the normalized sequence abundance for that sequence in that round. Missing values were assigned an abundance of 1.

The fitness value was then calculated by dividing the round 8b abundance by the round 4 abundance, and then dividing the resulting enrichment value by 20. A ceiling was introduced such that enrichment values greater than 1 were then adjusted to 0.999. This gave us ~2000 sequences with fitness greater than 0.25, which seemed to align with what we might expect given the sequence similarities within the top 2000 sequences. Fitness values were not corrected for HFNAP translation biases because improved translation characteristics can be a positive side effect of HFNAP in vitro selection. These translation biases arise largely from ligase sequence preferences, side-chain induced translation, or reverse translation biases^23^. Previous work has demonstrated that HFNAP sequences are recovered faithfully during reverse translation^16,21,23^. The 256-member high-fitness test set was chosen by ranking the sequences by enrichment (round 4 to round 8b). Every other highest-ranking sequence was included in the high-fitness test set, which prevented withholding the best 256 sequences from the data.

### Model construction and training

To learn the relationship between HFNAP sequence space and daunomycin binding, we begin by positing that the relationship is a function of a hidden (latent) variable ${{{{{{{\bf{z}}}}}}}}$. To learn the posterior distribution $p({{{{{{{\bf{z}}}}}}}}\vert{{{{{{{\bf{x}}}}}}}})$ over the latent ${{{{{{{\bf{z}}}}}}}}$, we can apply Bayes' Theorem given our observed data ${{{{{{{\bf{x}}}}}}}}$.

1 $${p}_{\theta }\left({{{{{{{\bf{z}}}}}}}}\vert{{{{{{{\bf{x}}}}}}}}\right)=\frac{{p}_{\theta }\left({{{{{{{\bf{x}}}}}}}}\vert{{{{{{{\bf{z}}}}}}}}\right){p}_{\theta }\left({{{{{{{\bf{z}}}}}}}}\right)}{p\left({{{{{{{\bf{x}}}}}}}}\right)}=\frac{{p}_{\theta }({{{{{{{\bf{x}}}}}}}},{{{{{{{\bf{z}}}}}}}})}{p({{{{{{{\bf{x}}}}}}}})}$$

However, computing ${p}_{\theta }\left({{{{{{{\bf{x}}}}}}}}\right)$ is intractable because it scales exponentially with the size of the latent space.

2 $${p}_{\theta }\left({{{{{{{\bf{x}}}}}}}}\right)=\int {p}_{\theta }\left({{{{{{{\bf{x}}}}}}}},{{{{{{{\bf{z}}}}}}}}\right)d{{{{{{{\bf{z}}}}}}}}=\int {p}_{\theta }\left({{{{{{{\bf{x}}}}}}}}{{{{\vert}}}}{{{{{{{\bf{z}}}}}}}}\right){p}_{\theta }({{{{{{{\bf{z}}}}}}}})d{{{{{{{\bf{z}}}}}}}}$$

Instead, we can use variational inference to approximate the posterior ${p}_{\theta }\left({{{{{{{\bf{z}}}}}}}}\vert{{{{{{{\bf{x}}}}}}}}\right)$, resulting in a variational distribution $q$ with parameters $\phi$^40,70^.

3 $${p}_{\theta }\left({{{{{{{\bf{z}}}}}}}}\vert{{{{{{{\bf{x}}}}}}}}\right)\,\approx\, {q}_{\phi }({{{{{{{\bf{z}}}}}}}}\vert{{{{{{{\bf{x}}}}}}}})$$

Because approximation ${q}_{\phi }({{{{{{{\bf{z}}}}}}}}\vert{{{{{{{\bf{x}}}}}}}})$ and ${p}_{\theta }\left({{{{{{{\bf{x}}}}}}}},{{{{{{{\bf{z}}}}}}}}\right)$ are tractable, we can find the marginal likelihood of ${p}_{\theta }\left({{{{{{{\bf{x}}}}}}}}\right)$.

4 $$\log{p}_{\theta }\left({{{{{\bf{x}}}}}}\right) 	={\mathbb{E}}_{{q}_{\phi }\left({{{{{\bf{z}}}}}}\vert{{{{{\bf{x}}}}}}\right)}\left[{\log}\,{p}_{\theta}\left({{{{{\bf{x}}}}}}\right)\right] \\ 	={\mathbb{E}}_{{q}_{\phi}\left({{{{{\bf{z}}}}}}\vert{{{{{\bf{x}}}}}}\right)}\left[{\log}\left[\frac{{p}_{\theta }\left({{{{{\bf{x}}}}}},{{{{{\bf{z}}}}}}\right)}{{p}_{\theta }\left({{{{{\bf{z}}}}}}\vert{{{{{\bf{x}}}}}}\right)}\right]\right] \\ 	={\mathbb{E}}_{{q}_{\phi }\left({{{{{\bf{z}}}}}}\vert{{{{{\bf{x}}}}}}\right)}\left[{\log}\left[\frac{{p}_{\theta}\left({{{{{\bf{x}}}}}},{{{{{\bf{z}}}}}}\right)}{{q}_{\phi }\left({{{{{\bf{z}}}}}}\vert{{{{{\bf{x}}}}}}\right)}\right]\right]+{\mathbb{E}}_{{q}_{\phi }\left({{{{{\bf{z}}}}}}\vert{{{{{\bf{x}}}}}}\right)}\left[{\log}\left[\frac{{q}_{\phi }\left({{{{{\bf{z}}}}}}\vert{{{{{\bf{x}}}}}}\right)}{{p}_{\theta}\left({{{{{\bf{z}}}}}}\vert{{{{{\bf{x}}}}}}\right)}\right]\right] \\ 	={{{{{\mathcal{L}}}}}}_{\theta,\phi }({{{{{\bf{x}}}}}})+{D}_{{{{{\rm{KL}}}}}}\left({q}_{\phi }\left({{{{{\bf{z}}}}}}{\vert}{{{{{\bf{x}}}}}}\right){\vert\vert}{p}_{\theta }\left({{{{{\bf{z}}}}}}\vert{{{{{\bf{x}}}}}}\right)\right)$$

The first term is the evidence lower bound (ELBO), and the second term is the Kullback-Leibler (KL) divergence. Because the KL divergence is always non-negative, the ELBO becomes the lower bound of ${{\log }}\,{p}_{\theta }\left({{{{{{{\bf{x}}}}}}}}\right)$.

5 $${{{{{{{{\mathcal{L}}}}}}}}}_{\theta,\phi }\left({{{{{{{\bf{x}}}}}}}}\right) 	={{\log }}\,{p}_{\theta }\left({{{{{{{\bf{x}}}}}}}}\right)-{D}_{{{{{{{{\rm{KL}}}}}}}}}\left({q}_{\phi }\left({{{{{{{\bf{z}}}}}}}}{{{{\vert}}}}{{{{{{{\bf{x}}}}}}}}\right){{{{\vert\vert}}}}{p}_{\theta }\left({{{{{{{\bf{z}}}}}}}}\vert{{{{{{{\bf{x}}}}}}}}\right)\right)\\ {{{{{{{{\mathcal{L}}}}}}}}}_{\theta,\phi }\left({{{{{{{\bf{x}}}}}}}}\right) 	 \le {{\log }}\,{p}_{\theta }\left({{{{{{{\bf{x}}}}}}}}\right)-{D}_{{{{{{{{\rm{KL}}}}}}}}}\left({q}_{\phi }\left({{{{{{{\bf{z}}}}}}}}{{{{\vert}}}}{{{{{{{\bf{x}}}}}}}}\right){{{{\vert\vert}}}}{p}_{\theta }\left({{{{{{{\bf{z}}}}}}}}\vert{{{{{{{\bf{x}}}}}}}}\right)\right)$$

Therefore, maximizing the ELBO will simultaneously maximize the log-likelihood of the model ${p}_{\theta }\left({{{{{{{\bf{x}}}}}}}}\right)$ and minimize the KL divergence between the approximation ${q}_{\phi }({{{{{{{\bf{z}}}}}}}}\vert{{{{{{{\bf{x}}}}}}}})$ and the posterior ${p}_{\theta }\left({{{{{{{\bf{z}}}}}}}}\vert{{{{{{{\bf{x}}}}}}}}\right)$. Re-writing the ELBO can also capture a more intuitive understanding of the VAE objective function, where we see terms for the inference model ${q}_{\phi }\left({{{{{{{\bf{z}}}}}}}}\vert{{{{{{{\bf{x}}}}}}}}\right)$ and the generative model ${p}_{\theta }\left({{{{{{{\bf{x}}}}}}}}\vert{{{{{{{\bf{z}}}}}}}}\right)$.

6 $${{{{{\mathcal{L}}}}}}_{\theta,\phi }\left({{{{{\bf{x}}}}}}\right)={\mathbb{E}}_{{q}_{\phi }\left({{{{{\bf{z}}}}}}{{\vert}}{{{{{\bf{x}}}}}}\right)}\left[{\log}\,{p}_{\theta }\left({{{{{\bf{x}}}}}}{\vert}{{{{{\bf{z}}}}}}\right)\right]-{D}_{{{{{\rm{KL}}}}}}\left({q}_{\phi }\left({{{{{\bf{z}}}}}}{\vert}{{{{{\bf{x}}}}}}\right){\vert\vert}{p}_{\theta}({{{{{\bf{z}}}}}})\right)$$

Here we see that the objective is to maximize the log-likelihood of reconstructing sequences and minimize the KL divergence between the inference model ${q}_{\phi }({{{{{{{\bf{z}}}}}}}}\vert{{{{{{{\bf{x}}}}}}}})$ and the prior ${p}_{\theta }({{{{{{{\bf{z}}}}}}}})$. To take gradients of the ELBO, we used the reparameterization trick^40,71^. In practice, we incorporated convex combination linear inverse autoregressive flows to obtain full rank covariance Gaussian posteriors^72^.

Models were built using Python 3.6 and PyTorch 1.3.1. Sequences were input to the model as 15mer vectors, where 45mer ACGT sequences were converted to 15mers of numbers ranging from 0-31 based on the 32 available building blocks for HFNAP translation. Sequence vector batches were then fed through an embedding layer (32,15), resulting in a (batch, 15,15) tensor output that is passed to the encoder. The encoder consisted of bidirectional stateful LSTMs (2 layers, 200 nodes, tanh activations), which results in a (batch, 400) intermediate that is passed to a linear layer with tanh activation resulting in an encoder output (batch, 15). This matrix is then passed to two separate linear layers (batch,15), one for the mean and one for the log variance. The decoder used fully connected nodes. The sequence reconstruction network contained two layers (1×480)(32×15) and tanh activations. The fitness prediction network takes the output (32×15) matrix from the sequence reconstruction network, and passes it through a (1×480) layer with sigmoid activation to get the predicted fitness value. Fitness values are concatenated to the z’s and fed to the decoder. Reconstruction accuracy was determined by building block reconstruction accuracy, where the input 15mer was checked against the output 15mer. DNA reconstruction accuracy was used for qualitative analysis. Reconstruction loss is computed with a cross entropy loss, and fitness loss is computed by mean squared error. The loss is regularized using a KL divergence between the latent distribution and a multivariate Gaussian normal. The model was trained on the training set described previously, comprised of 172,545 selection 2 sequences, with corresponding fitness values. To select model architecture and hyperparameters, we empirically performed grid search across a range of values including the size and number of neural network layers, the layer architecture, the KL annealing, and the weighting between sequence reconstruction loss, fitness MSE, and the KL divergence. The sequence reconstruction loss weighting was varied between 1 to 15 and the fitness MSE weighting was varied between 100 to 200. KL annealing occurred over 5000 batches using a sigmoid annealing schedule^73^. The Adam optimizer was used with a learning rate of 0.001 and *β*_1_ = 0.9 and *β*_2_ = 0.99^74^.

### Generating HFNAP sequences with putative binding affinity for daunomycin

After model parameters were sufficiently trained and reconstruction loss was minimized, sequences were generated for testing. The top 3072 sequences as determined by fitness, was designated as the reference set. The reference set was fed into the trained model to calculate the $z$ distribution to sample from. The corresponding $z$ distribution was then randomly sampled, and the decoder used to generate 10,000 HFNAP sequences for testing. We varied the fitness in increments of 0.05 ranging from 0.5 to 0.95. This gave us 1000 sequences at each fitness value: 0.5, 0.55, … 0.95.

### Generated sequences selection competition

The 10,000 CVAE-generated HFNAP sequences and top 2000 sequences by fitness from selection 2 were synthesized (Twist Bioscience). The templates were amplified using a biotinylated primer, native PAGE purified on a 10% TBE gel, gel extracted, and DNA column purified. A mastermix of the building block distribution of the 12,000 sequences was then made, and used in the HFNAP translation. The translated sequences were then subjected to the high-stringency selection conditions, with 1 min, 10 min, and 60 min elutions. The 60 min elution sample was then DNA column purified, PCR amplified, and native PAGE purified on a 10% TBE gel. The gel extracted material was then DNA column purified and prepared for HTS as shown previously. The pre-selection library, a saved sample post translation and before selection, was prepared similarly. The two samples were subject to HTS on a NextSeq, and analyzed by AptaSuite^64^. The resulting data was used to calculate the enrichments of all of the original 12,000 sequences. The templates for the top 5 most enriched sequences from the 10,000 generated HFNAPs were then ordered from IDT. The templates were then used for HFNAP translations to prepare samples for MST characterization.

### Random sequences selection competition

10,000 reference-based random HFNAPs were sampled using the building block distribution frequency of the reference set. These HFNAPs were then subjected to the same selection that the 10,000 CVAE-generated HFNAPs underwent.

### Full fitness sequences selection competition

A total of 12,000 CVAE-generated HFNAPs were generated using the following fitness conditions: 0.001, 0.002, 0.003, 0.004, 0.005, 0.006, 0.007, 0.008, 0.009, 0.01, 0.02, 0.03, 0.04, 0.05, 0.06, 0.07, 0.08, 0.09, 0.1, 0.11, 0.12, 0.13, 0.14, 0.15, 0.16, 0.17, 0.18, 0.19, 0.2, 0.25, 0.3, 0.35, 0.4, 0.45, 0.5, 0.55, 0.6, 0.65, 0.7, 0.75, 0.8, 0.85, 0.9, 0.95, and 0.999. 250 sequences for each fitness ≤0.25, and 300 sequences for each fitness > 0.25. These HFNAPs were then subjected to the same selection used to process the previous 10,000 CVAE-generated HFNAPs.

### Reporting summary

Further information on research design is available in the Nature Research Reporting Summary linked to this article.

## Supplementary information

Supplementary Information

Reporting Summary

Supplementary Data 1

## Data Availability

The principal data supporting the findings of this work are available in the main text or the supplementary materials. High-throughput sequencing data will be available from the NCBI Sequence Read Archive under accession code PRJNA854957. Data used for training has been included in the Supplementary Information. Additional data and code that support the findings of this study are available from the authors on request.

## Code availability

Additional data and code that support the findings of this study are available from the authors on request.
