# Supplementary material for: Generating experimentally unrelated target molecule-binding highly functionalized nucleic-acid polymers using machine learning
Source: Nat Commun. 2022 Aug 4;13:4541. doi: 10.1038/s41467-022-31955-4 (PMC9352670; doi:10.1038/s41467-022-31955-4)
Supplement: Supplementary file 1 — Supplementary Information [file 41467_2022_31955_MOESM1_ESM.pdf]

Supplementary Information

Generating experimentally unrelated target molecule-binding highly functionalized nucleic-acid polymers using machine learning

Jonathan C. Chen[1-3], Jonathan P. Chen[4,5], Max W. Shen[1-3,6], Michael Wornow[1,2], Minwoo Bae[1,2], Wei-Hsi Yeh,[1-3,7] Alvin Hsu[1-3], David R. Liu[1-3]*

[1]Merkin Institute of Transformative Technologies in Healthcare, Broad Institute of Harvard and MIT, Cambridge, MA USA

[2]Department of Chemistry and Chemical Biology, Harvard University, Cambridge, MA, USA

[3]Howard Hughes Medical Institute, Harvard University, Cambridge, MA, USA

[4]Work conducted at Uber AI Labs, Uber Technologies, Inc., San Francisco, CA, USA

[5]Meta Platforms, Menlo Park, CA, USA

[6]Computational and Systems Biology Program, Massachusetts Institute of Technology, Cambridge, MA, USA

[7]Program in Speech and Hearing Bioscience and Technology, Harvard Medical School, Boston, MA, USA

*Correspondence to: drliu@fas.harvard.edu

Supplementary Table 1 | Binding affinities for top four most enriched daunomycin-binding HFNAPs from selection 1. 3
Supplementary Table 2 | MST characterization of daunomycin binding affinity for mutants of Dm-HS-1 lacking side-chains. 5
Supplementary Table 4 | Daunomycin K_d measurements of CVAE-generated sequences after selection. 6
Supplementary Figure 1 | Quantifying selection progress by comparing non-binding flow-throughs to the eluent.7
Supplementary Figure 2 | Differences in enrichment values between selection 1 and selection 2. 8
Supplementary Figure 3 | Changes in side-chain distribution for selection 2. 9
Supplementary Figure 4 | Changes in side-chain distribution for selection 1. 10
Supplementary Figure 5 | Performance of selection-1 sequences in selection 2. 11
Supplementary Figure 6 | MST characterization of daunomycin affinity for the top 10 most enriched sequences from selection 2. 12
Supplementary Figure 7 | Gel filtration characterization of Dm-HS-1 daunomycin affinity. 13
Supplementary Figure 8 | Distribution of fitness values for training and general test set sequences. 15
Supplementary Figure 9 | CVAE performance of an early model. 16
Supplementary Figure 10 | tSNE plot of general fitness test set sequences and high fitness test set sequences. 17
Supplementary Figure 11 | Percent of training sequences with at least 3-6 alcohol or amine side-chains. 18
Supplementary Figure 12 | Percent of CVAE-generated sequences with at least 3-6 alcohol or amine side-chains. 19
Supplementary Figure 13 | Pairwise Levenshtein distances for 10,000 CVAE-generated HFNAPs. 20
Supplementary Figure 14 | Pairwise Levenshtein distances for 10,000 truly random HFNAPs. 21
Supplementary Figure 15 | High-stringency selection of mixture of 10,000 CVAE-generated HFNAPs and 2,000 most highly enriched sequences from round 4 to round 8b of selection 2 (competition set). 22
Supplementary Figure 16 | Enrichments from the competition selection. 23
Supplementary Figure 17 | Aggregate enrichments from the 12,000 CVAE-generated HFNAP competition selection. 24
Supplementary Figure 18 | High-stringency selection of mixture of 10,000 reference-based random HFNAPs and 2,000 most highly enriched sequences from round 4 to round 8b of selection 2 (competition set). 26
Supplementary Figure 19 | MST characterization of the five most enriched sequences from the reference-based random set after selection. 27
Supplementary Figure 20 | Comparison of the Levenshtein distances from the 3,072-member reference set to the top seven sequences from each of the four groups. 28
Supplementary Figure 21 | Pairwise Levenshtein distances between seven sequences and 172,545-member training set. 29
Supplementary Figure 22 | Pairwise Levenshtein distances from reference set to 10,000-member libraries. 30
Supplementary Figure 23 | Pairwise Levenshtein distances from reference set to 10,000-member libraries. 31
Supplementary Figure 24 | Distance distributions for pairwise Levenshtein distances for seven validated CVAE-generated HFNAP sequences, seven validated selection-2 HFNAP sequences, seven reference-based random HFNAP sequences, and seven truly random HFNAP sequences. 32
Supplementary Figure 25 | Pairwise Levenshtein distances amongst 10,000-member libraries. 33
Supplementary Figure 26 | Predicted secondary structures for Dm-HS-1 from RNAstructure. 34
Supplementary Figure 27 | Predicted secondary structures for CVAE-generated sequences after competition selection with qualitative structural similarities to Dm-HS-1 predicted structures. 35
Supplementary Figure 28 | Base-pair-edit distance between Dm-HS-1 structures and predicted secondary structures from CVAE-generated sequences and reference-based random sequences. 36
Supplementary Figure 29 | Distribution of SCI scores for CVAE-generated sequences, reference-based random sequences, and selection-2 sequences to Dm-HS-1-10. 37
Supplementary Figure 30 | Distribution of SCI scores and Levenshtein distances for CVAE-generated sequences, reference-based random sequences, and selection-2 sequences to Dm-HS-1-5. 38
Supplementary Figure 31 | Distribution of SCI scores and Levenshtein distances for CVAE-generated sequences, reference-based random sequences, and selection-2 sequences to Dm-HS-6-10. 39
Supplementary Figure 32 | Number of sequences containing motifs 1-3 for a given E value in 10,000 CVAE-generated sequences and 10,000 reference-based random sequences. 40
Supplementary Figure 33 | HFNAP positioning of sequence-structural motifs identified by AptaTrace in CVAE-generated sequences, reference-based random sequences, and selection 2 round 8b sequences. 41
Supplementary Figure 34 | Distribution of SCI scores for CVAE-generated sequences and reference-based random sequences to Dm-HS-10, -5, and -8 for motifs 1-3, respectively. 42
Supplementary Note 1 | Oligonucleotides used in selections, HFNAP preparation, and HTS. 40
Supplementary Note 2 | Mass spectrum of negative selection target. 43
Supplementary Data 1 | Excel file with HFNAP sequences from training data, 10,000 sequence sets, and competition set. 44

Sequence	K_d
Dm-LS-1	1.11 ± 0.06 μM
Dm-LS-2	0.97 ± 0.06 μM
Dm-LS-3	2.23 ± 0.10 μM
Dm-LS-4	1.98 ± 0.07 μM

Supplementary Table 1 | Binding affinities for top four most enriched daunomycin-binding HFNAPs from selection 1. Binding affinity for daunomycin was measured by microscale thermophoresis (MST). The mean and SEM of n = 3 independent replicates are shown.

Codon	Sidechain	K_d	K_d change
CGA	Fluorophenyl	231.1 ± 23.6 nM	18x
TTC	Allylamine	16.2 ± 3.2 nM	1.25x
TGG	Allylamine	16.0 ± 2.2 nM	1.25x
TGC	Alcohol (2x)	20.3 ± 4.4 nM	1.6x
TGT	Phenol	6.7 ± 1.0 nM	0.52x
TGA	Alcohol	46.8 ± 4.8 nM	3.6x
TCT	Allylamine	12.9 ± 1.5 nM	1x
CCG	Cyclopentyl	21.9 ± 2.6 nM	1.7x
CCA	Cyclopropyl	158.8 ± 14.2 nM	12x
TTG	Phenol	32.6 ± 5.0 nM	2.5x
TCC	Imidazole	29.9 ± 10.2 nM	2.3x
TAC	Imidazole	21.7 ± 2.1 nM	1.7x
CCT	Cyclopropyl	18.8 ± 2.6 nM	1.5x
TCA	Imidazole	10.1 ± 1.6 nM	0.8x

Supplementary Table 2 | MST characterization of daunomycin binding affinity for mutants of Dm-HS-1 lacking side-chains. Fourteen single mutants of Dm-HS-1 were translated without side-chains for the indicated codon position. MST characterization of the binding affinities of the resulting mutants are shown. The K_d change is relative to unmutated Dm-HS-1. The mean and SEM of n = 3 independent replicates are shown.

CVAE-generated sequences without selection	K_d
Dm-CVAE-without-selection-1	13.2 ± 3.2 nM
Dm-CVAE-without-selection-2	15.5 ± 1.3 nM

Supplementary Table 3 | Daunomycin K_d measurements of CVAE-generated HFNAPs without selection. MST was used to measure binding affinity for daunomycin. The mean and SEM of n = 3 independent replicates are shown.

CVAE-generated sequences after selection	K_d
Dm-CCS-1	16.7 ± 6.0 nM
Dm-CCS-2	16.6 ± 14.0 nM
Dm-CCS-3	26.4 ± 6.9 nM
Dm-CCS-4	8.5 ± 2.3 nM
Dm-CCS-5	10.4 ± 1.5 nM

Supplementary Table 4 | Daunomycin K_d measurements of CVAE-generated sequences after selection. MST was used to measure binding affinity for daunomycin. The mean and SEM of n = 3 independent replicates are shown.

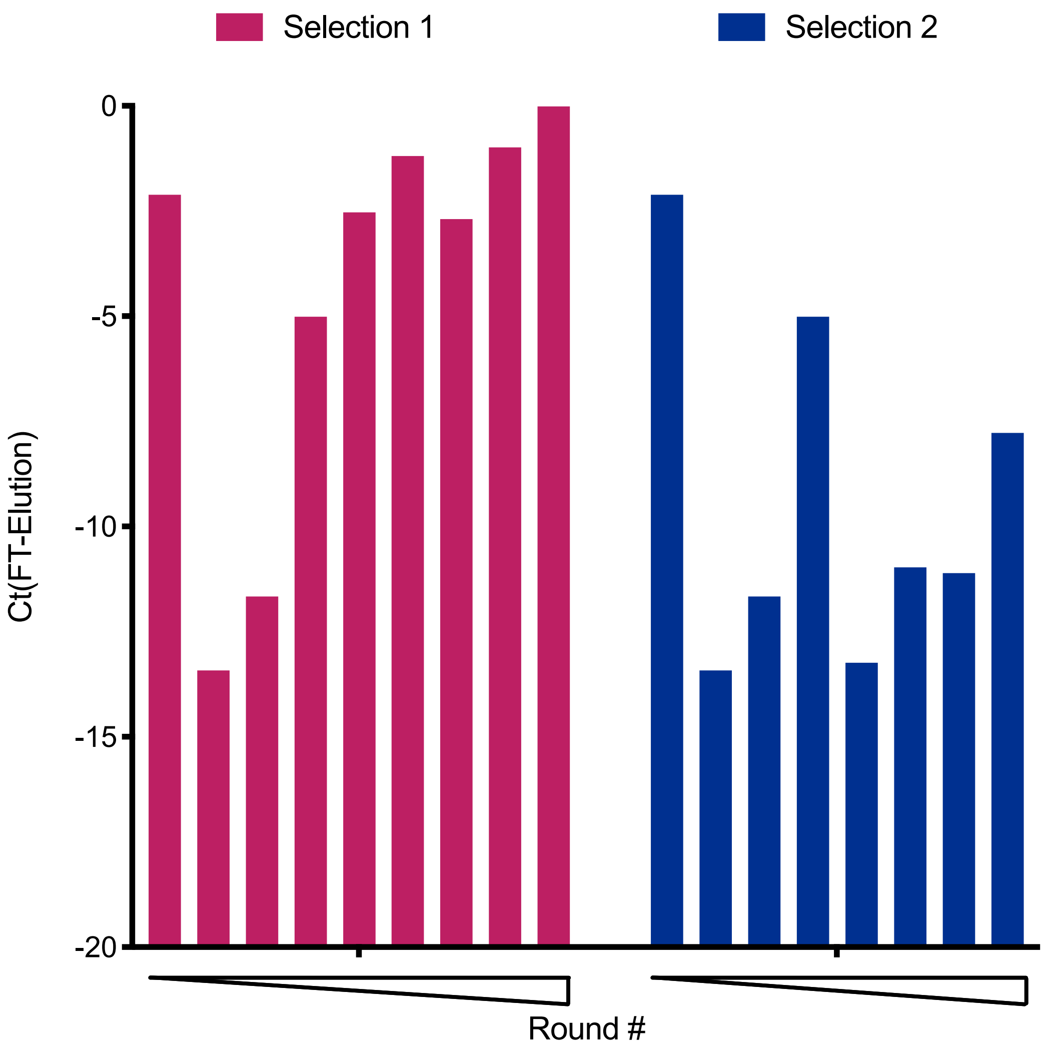

Supplementary Figure 1 | Quantifying selection progress by comparing non-binding flow-throughs to the eluent. Selection 1 and selection 2 progress was followed by qPCR Ct value. Higher bars indicate more HFNAPs were found in the elution compared to the flow-through.

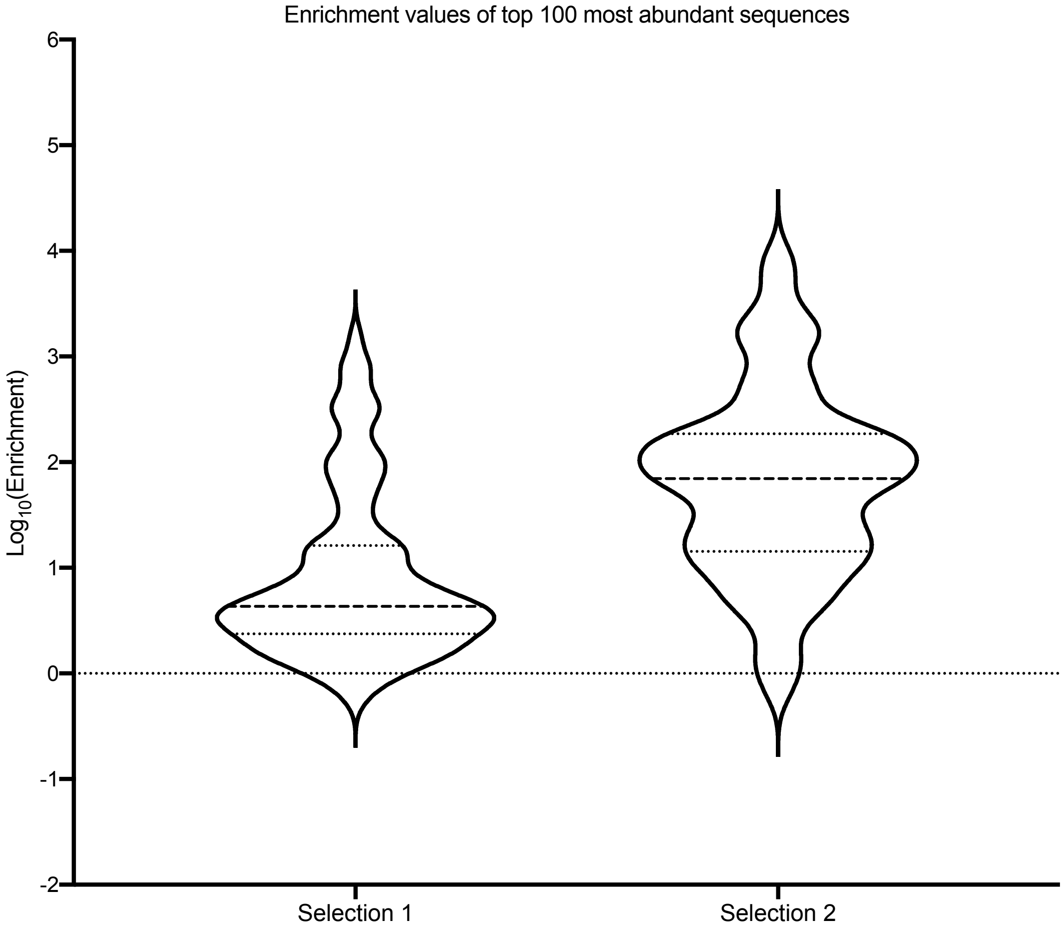

Median enrichment values			
Top	Selection 1	Selection 2	Fold-change
10	14.9	1635	109.5
20	10.1	142	14.0
25	8.0	141	17.5
30	7.2	119	16.6
40	5.4	90	16.8
50	5.4	81	15.2
100	4.3	70	16.2

Average enrichment values			
Top	Selection 1	Selection 2	Fold-change
10	36.3	2581	71.1
20	36.4	1719	47.2
25	30.7	1466	47.8
30	26.3	1293	49.3
40	20.5	1042	50.7
50	64.2	848	13.2
100	69.3	481	6.9

Supplementary Figure 2 | Differences in enrichment values between selection 1 and selection 2. Violin plot center line designates the median; dotted line used for upper and lower quartiles. Selection 1 enrichment values were calculated by dividing each HFNAP sequence's frequency in the last round of selection 1 (9a) by its frequency in round 4. Selection 2 enrichment values were calculated by dividing the frequency of each HFNAP sequence in the last round of selection 2 (8b) by its frequency in round 4. If sequences were not found in round 4, the subsequent round frequency was used. A $\log_{10}$(enrichment) value of zero denotes no change. Positive values indicate enrichment and negative values indicate de-enrichment.

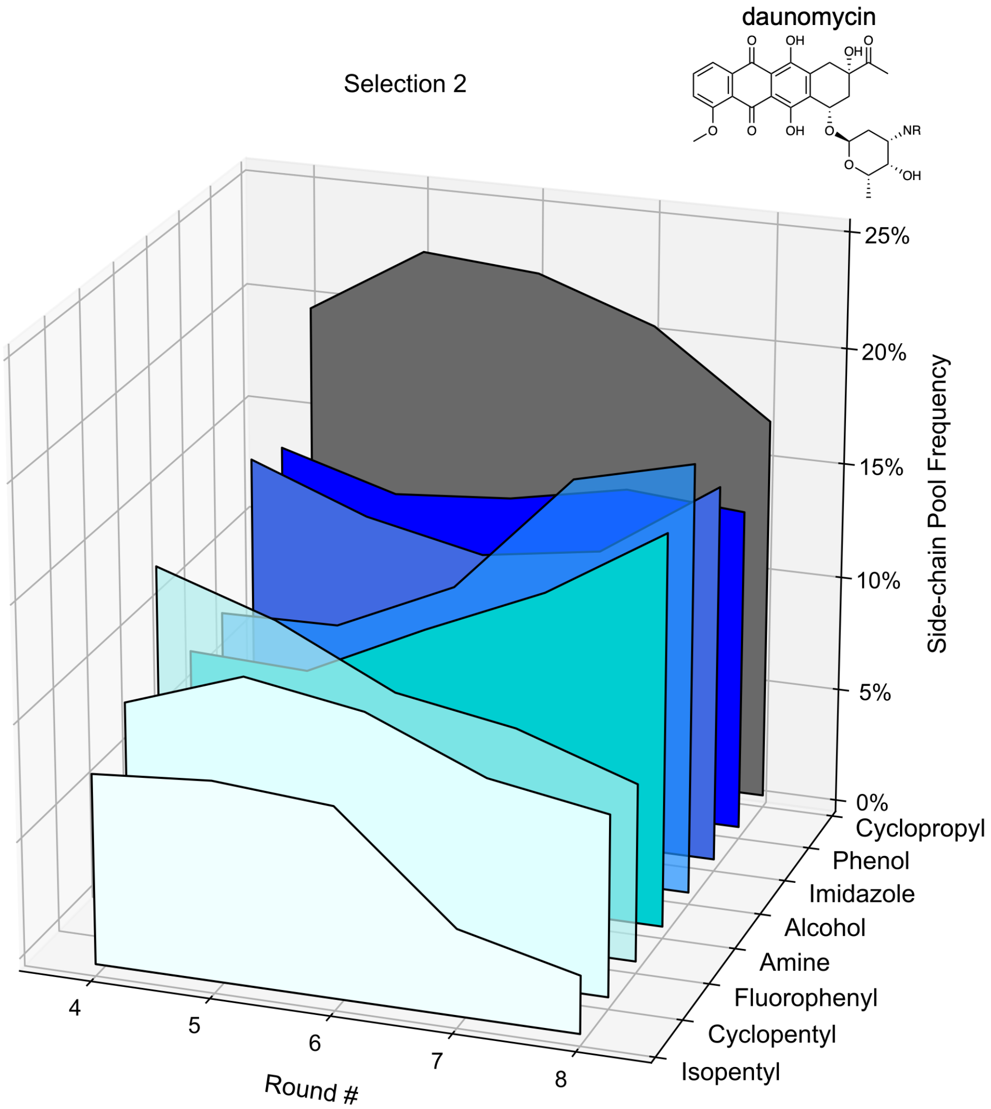

Supplementary Figure 3 | Changes in side-chain distribution for selection 2. Overall side-chain frequencies are plotted over the course of the selection. Decreases in the frequency of isopentyl, cyclopropyl and fluorophenyl side-chains are accompanied by increases in the frequency of amine and alcohol side-chains. Daunomycin (daunorubicin) structure is shown for reference, with R referring to the linker (see Methods).

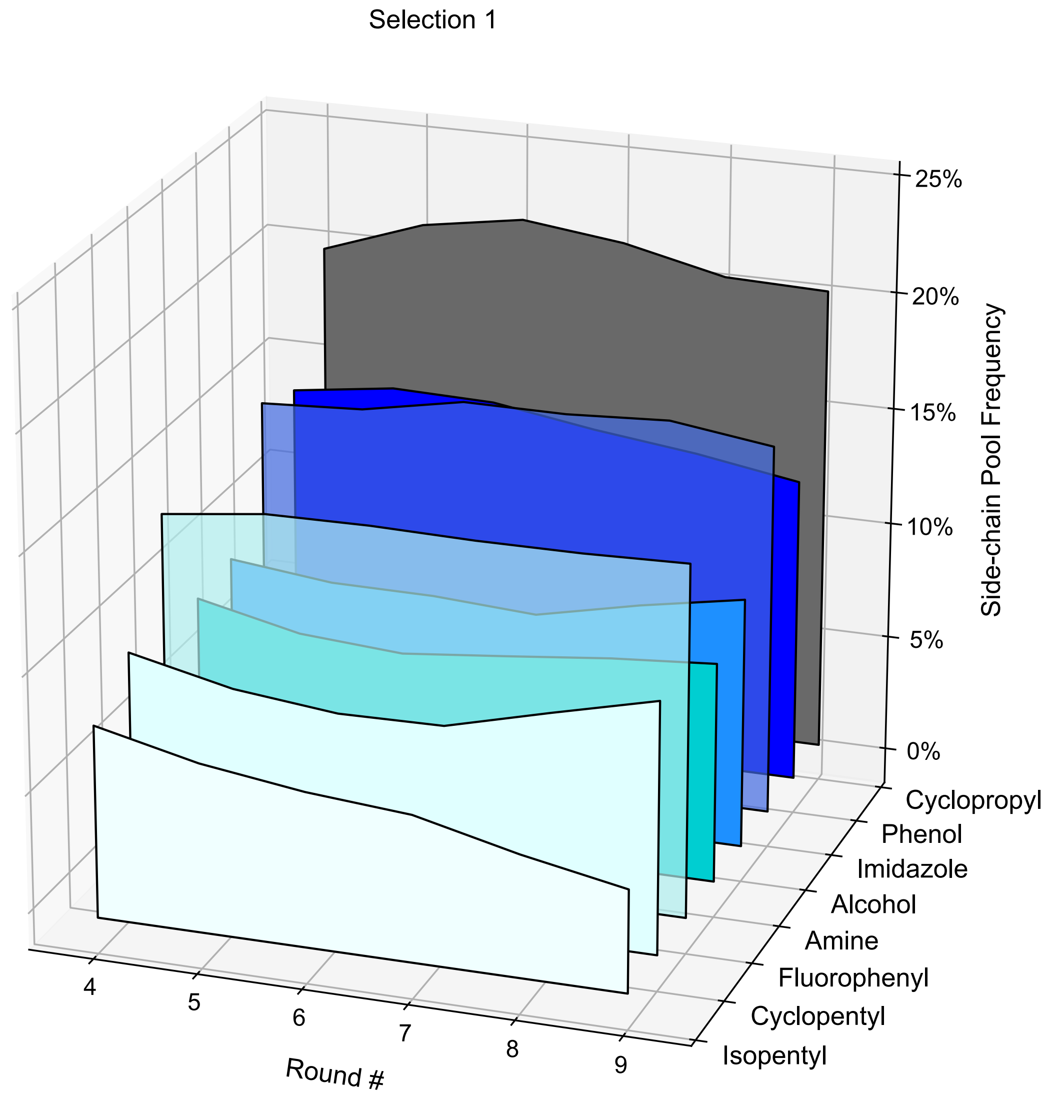

Supplementary Figure 4 | Changes in side-chain distribution for selection 1. Overall side-chain frequencies are plotted over the course of the selection for daunomycin binding. Minimal changes to the side-chain frequencies are observed.

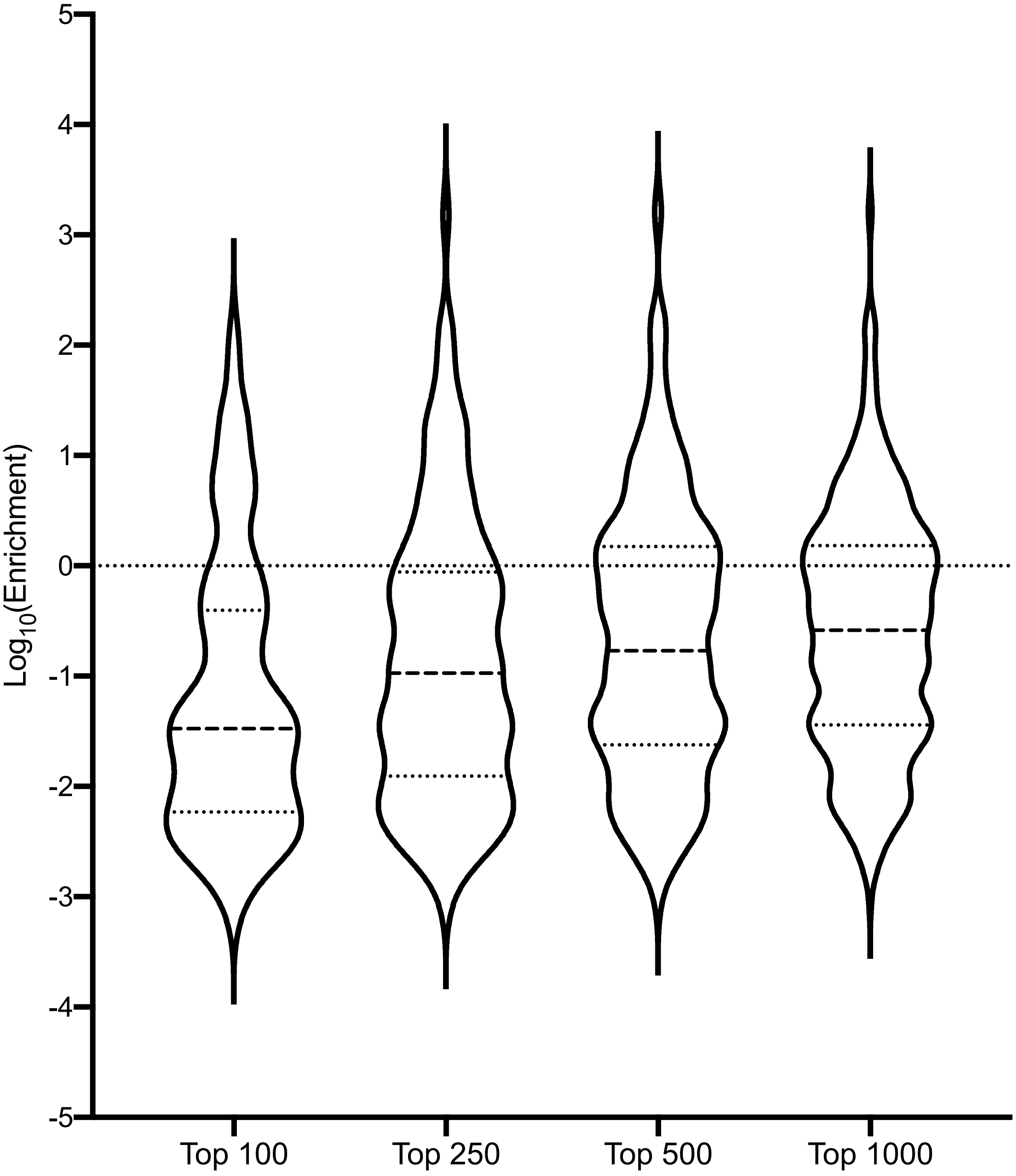

Supplementary Figure 5 | Performance of selection-1 sequences in selection 2. Violin plot center line designates the median; dotted line used for upper and lower quartiles. The selection 2 enrichment values of the top 1000 sequences from selection 1 (by relative abundance) were calculated. The 1000 selection 1 sequences were identified in selection 2, and their sequence frequencies at the end of selection 2 (round 8b) were divided by the sequence frequencies at the start of selection 2 (round 4). If sequences were not found in round 4, the subsequent round frequency was used. If sequences were not found in round 8, the previous round frequency was used. A $\log_{10}$(enrichment) value of 0 denotes no change. Positive values indicate enrichment and negative values indicate de-enrichment.

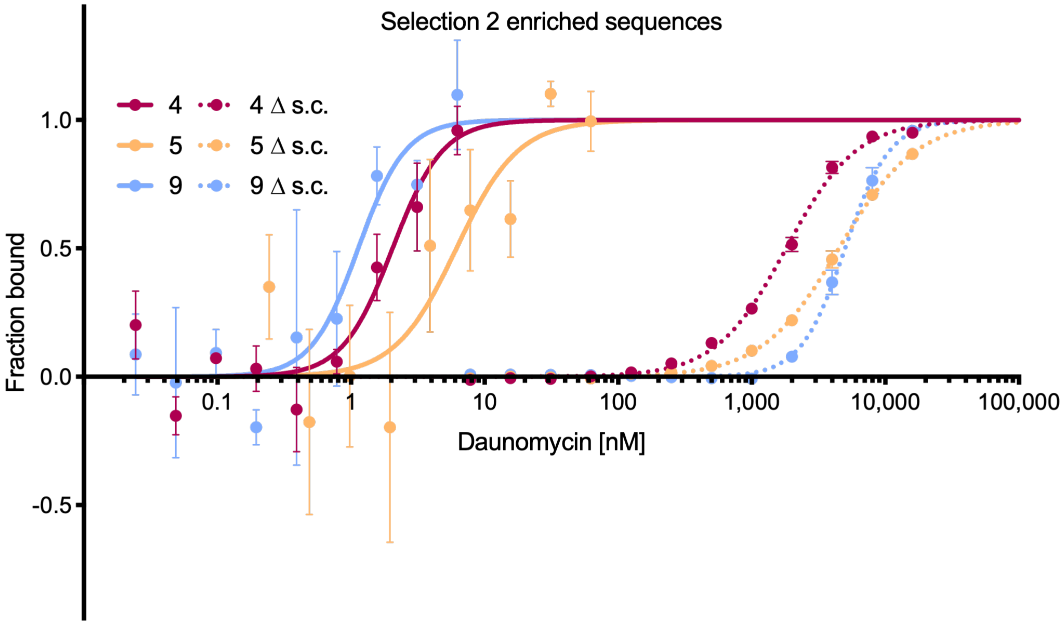

Sequence	K_d	K_d Δ side-chains	K_d change
Dm-HS-1	12.85 ± 1.6 nM	2.61 ± 0.19 μM	200x
Dm-HS-2	4.77 ± 1.0 nM	1.70 ± 0.17 μM	350x
Dm-HS-3	4.78 ± 0.95 nM	4.45 ± 0.28 μM	930x
Dm-HS-4	2.05 ± 0.65 nM	1.81 ± 0.057 μM	880x
Dm-HS-5	6.16 ± 3.4 nM	4.50 ± 0.21 μM	730x
Dm-HS-6	26.86 ± 4.2 nM	1.51 ± 0.065 μM	56x
Dm-HS-7	18.09 ± 5.2 nM	2.49 ± 0.12 μM	140x
Dm-HS-8	32.26 ± 8.9 nM	4.84 ± 0.26 μM	150x
Dm-HS-9	1.13 ± 0.45 nM	5.00 ± 0.14 μM	4000x
Dm-HS-10	64.68 ± 3.9 nM	0.713 ± 0.028 μM	11x

Supplementary Figure 6 | MST characterization of daunomycin affinity for the top 10 most enriched sequences from selection 2. Dm-HS-4 and Dm-HS-5 differ from Dm-HS-1 by one nucleotide, and Dm-HS-9 differs from Dm-HS-3 by one nucleotide. The mean and SEM of n = 3 independent replicates are shown. Δ s.c. denotes sequences without side-chains. The mean and SEM of n = 3 independent replicates are shown.

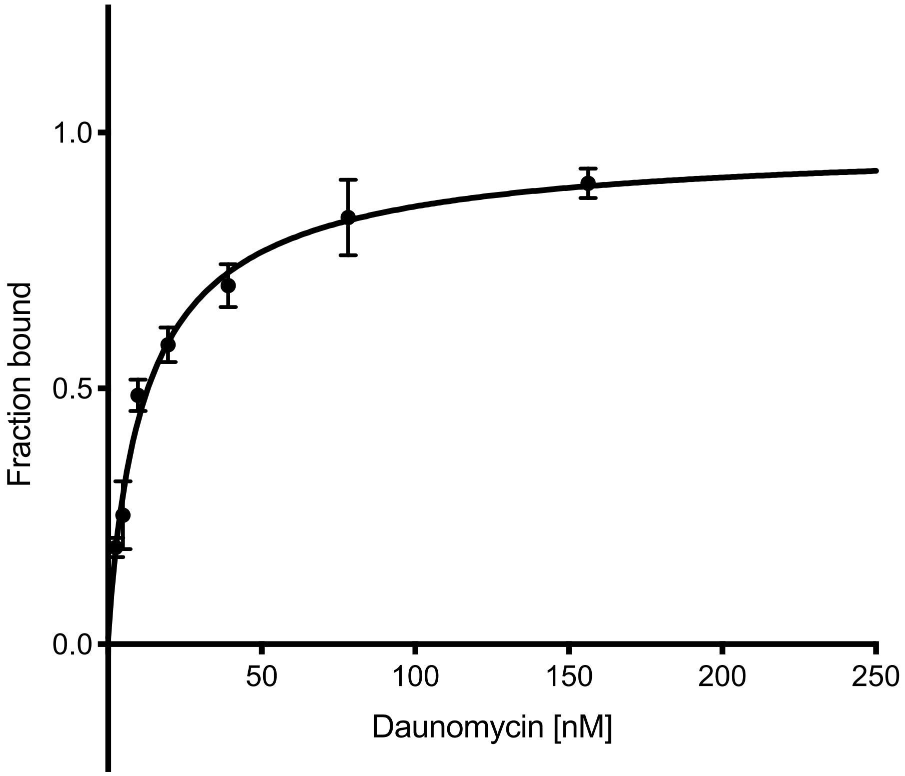

Supplementary Figure 7 | Gel filtration characterization of Dm-HS-1 daunomycin affinity. ^{3}H-daunomycin was used to characterize Dm-HS-1 daunomycin affinity in a gel filtration assay. The mean and SEM of n = 3 independent replicates are shown. K_d = 12.6 ± 3.5 nM which reflects mean and SEM of n = 3 independent replicates.

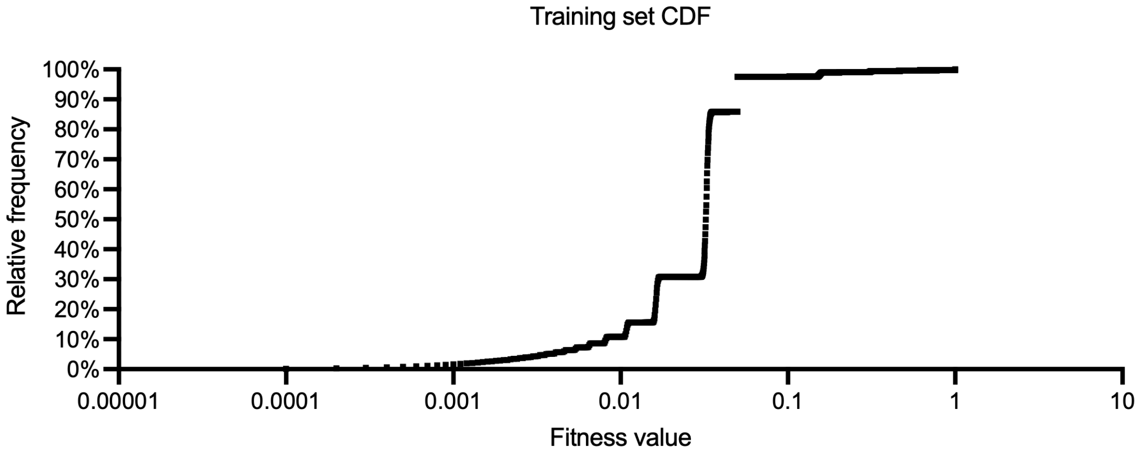
Training set CDF
Relative frequency
100%
90%
80%
70%
60%
50%
40%
30%
20%
10%
0%
0.00001
0.0001
0.001
0.01
0.1
1
10
Fitness value

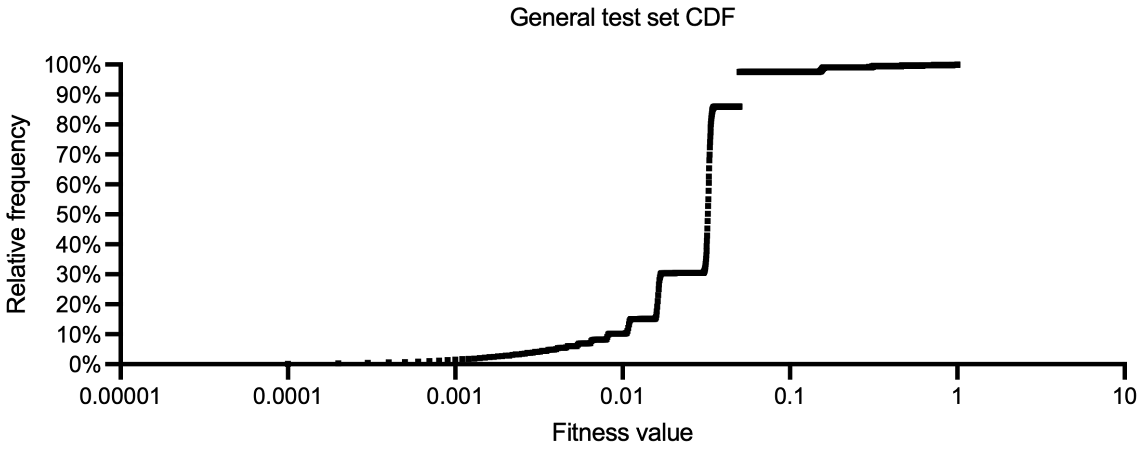
General test set CDF
Relative frequency
100%
90%
80%
70%
60%
50%
40%
30%
20%
10%
0%
0.00001
0.0001
0.001
0.01
0.1
1
10
Fitness value

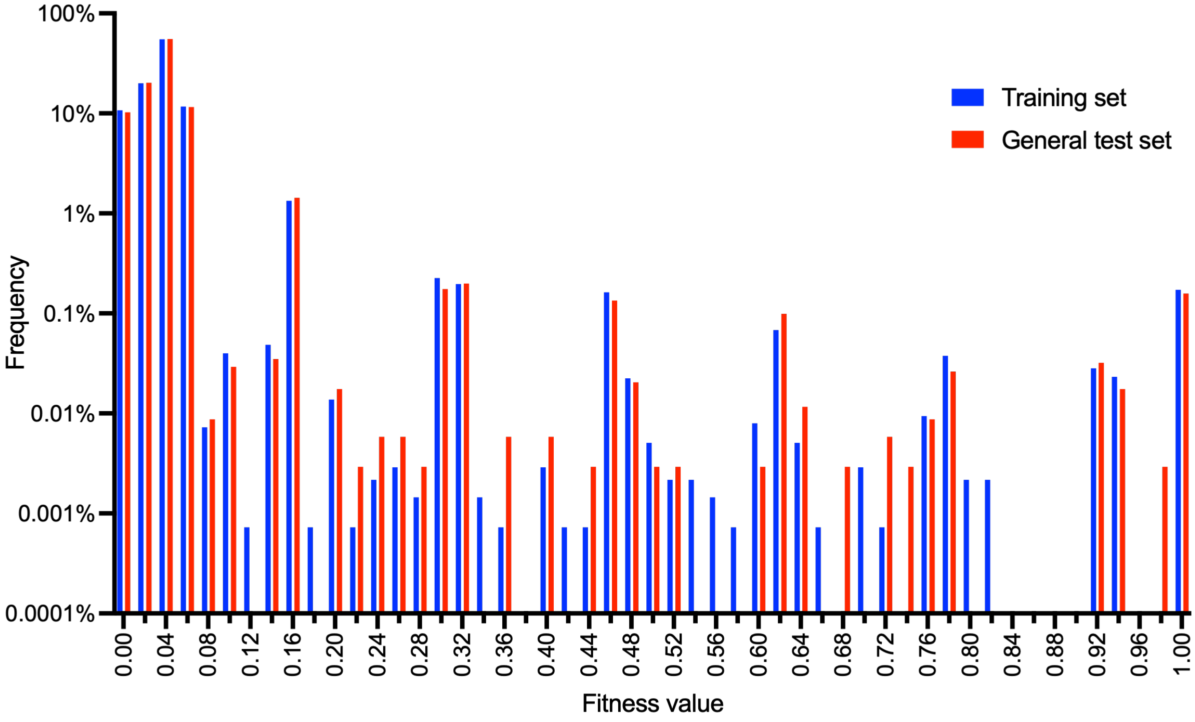
Frequency
100%
10%
1%
0.1%
0.01%
0.001%
0.0001%
Training set
General test set
0.00
0.04
0.08
0.12
0.16
0.20
0.24
0.28
0.32
0.36
0.40
0.44
0.48
0.52
0.56
0.60
0.64
0.68
0.72
0.76
0.80
0.84
0.88
0.92
0.96
1.00
Fitness value

Supplementary Figure 8 | Distribution of fitness values for training and general test set sequences. Selection 2 sequences were randomly assigned to the training set or general test set using an 80:20 ratio. Training set and general test set sequences are plotted against $\log_{10}$(fitness) and demonstrate comparable fitness distributions. The histogram of fitness values is then plotted with the bin center labeled on the X-axis. For example, the fitness value = 0.50 bin includes all sequences in the range: 0.49 ≥ fitness value > 0.51. Fitness values were assigned based on selection 2 enrichment values (see Methods), resulting in discontinuity in assigned fitness values.

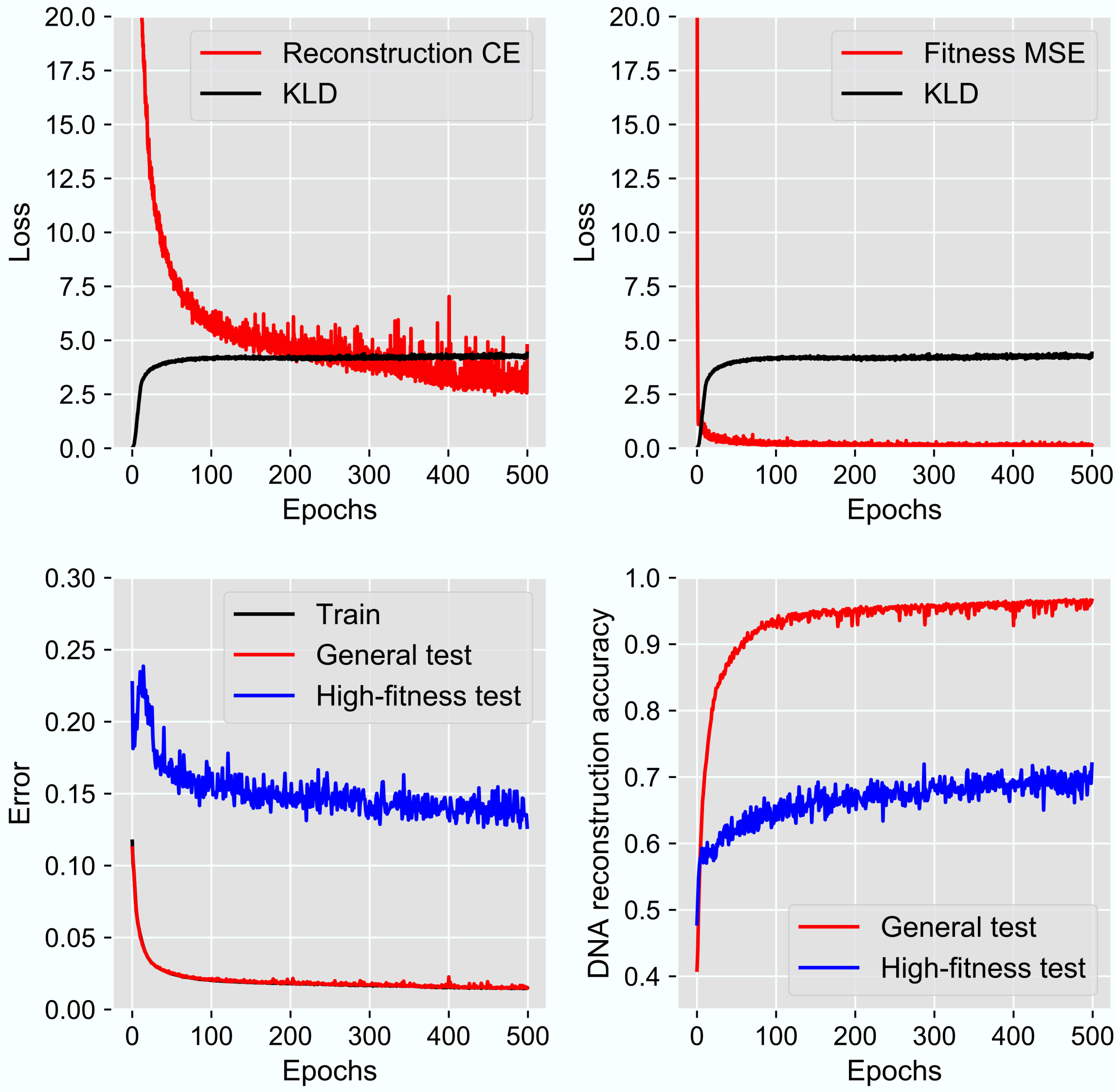

Supplementary Figure 9 | CVAE performance of an early model. Total sequence reconstruction loss as calculated by cross entropy and fitness reconstruction loss as calculated by mean-squared error are shown. Model DNA reconstruction accuracy on 20% held out general test set was high. Further analysis of the model reveals low DNA reconstruction accuracy for high-fitness sequences.

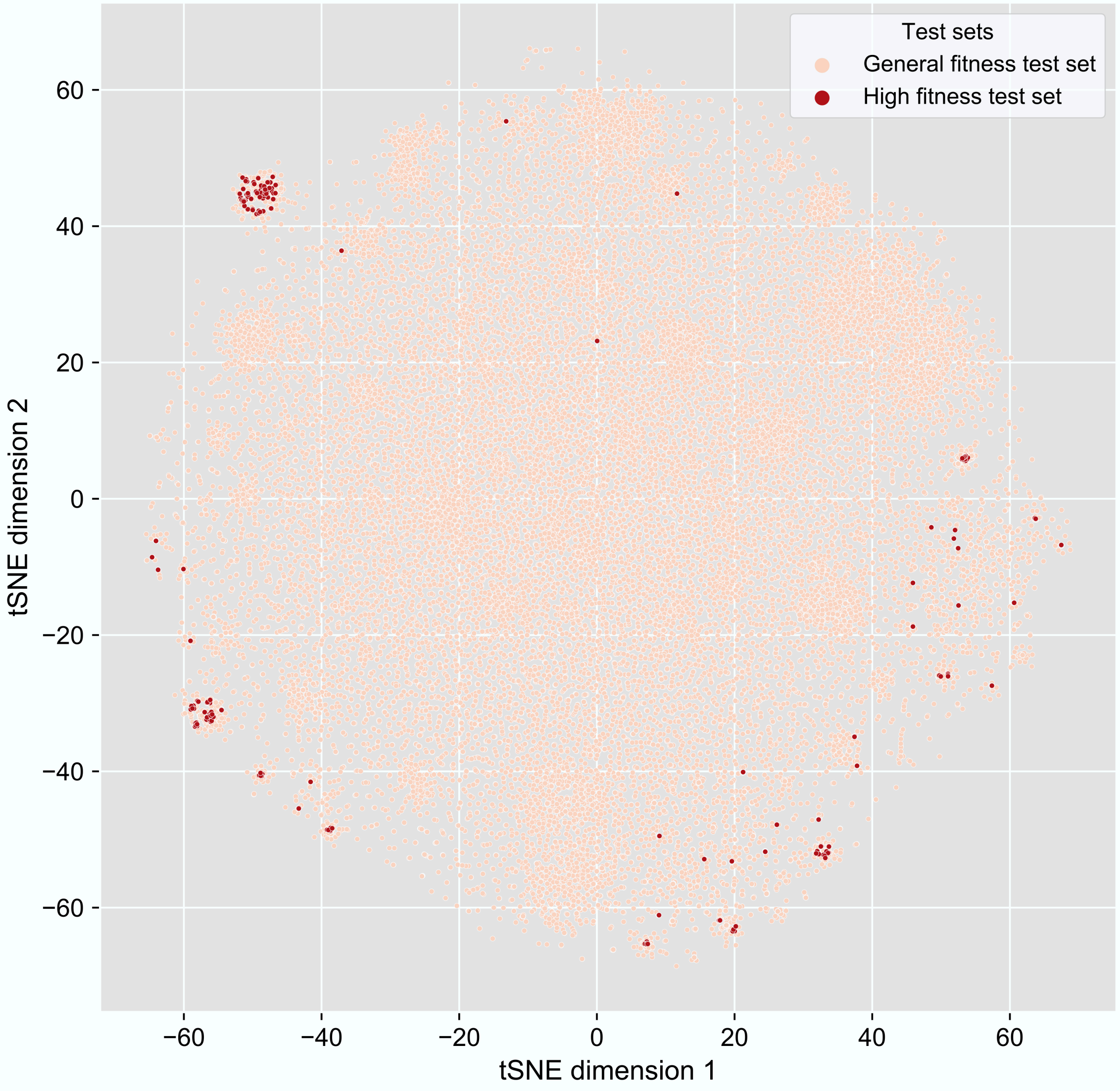

Supplementary Figure 10 | tSNE plot of general fitness test set sequences and high fitness test set sequences. Members of the high fitness test set are converged, as evidenced by local clustering of sequences.

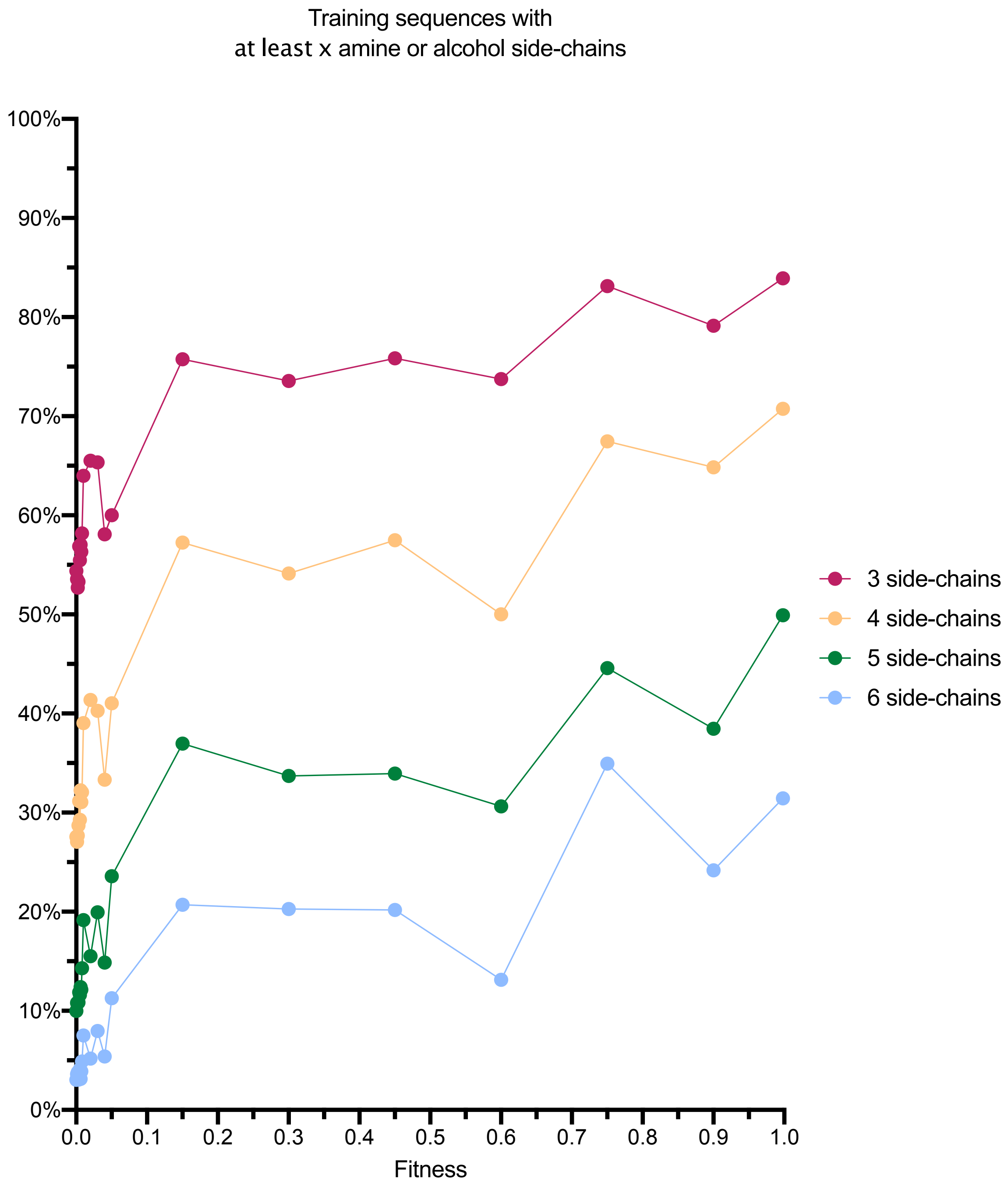

Supplementary Figure 11 | Percent of training sequences with at least 3-6 alcohol or amine side-chains. Selection sequences were grouped based on their fitness values. Each point along the X-axis represents the sequences with fitness greater than the x value and less than or equal to the next x value. For example, the 0.6 data point refer to all sequences with fitness >0.6 and ≤0.75.

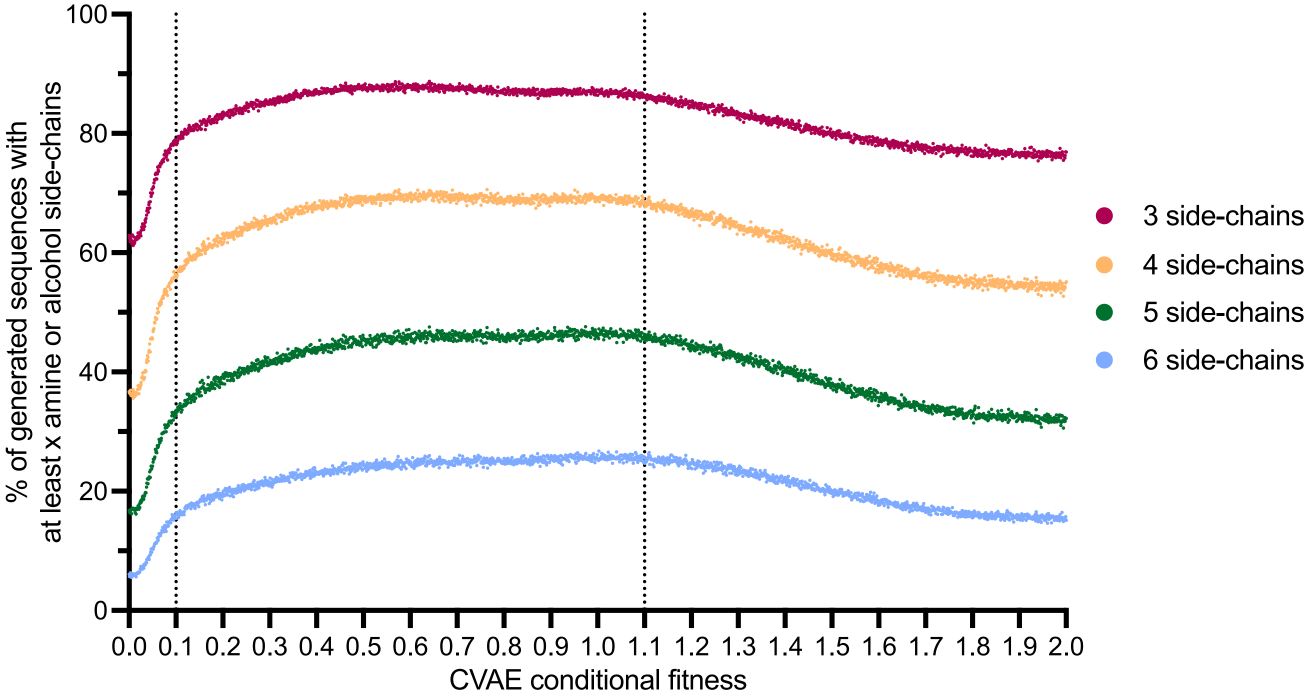

Supplementary Figure 12 | Percent of CVAE-generated sequences with at least 3-6 alcohol or amine side-chains. Each point along the x-axis represents 10,000 CVAE-generated sequences that were generated with the corresponding fitness condition.

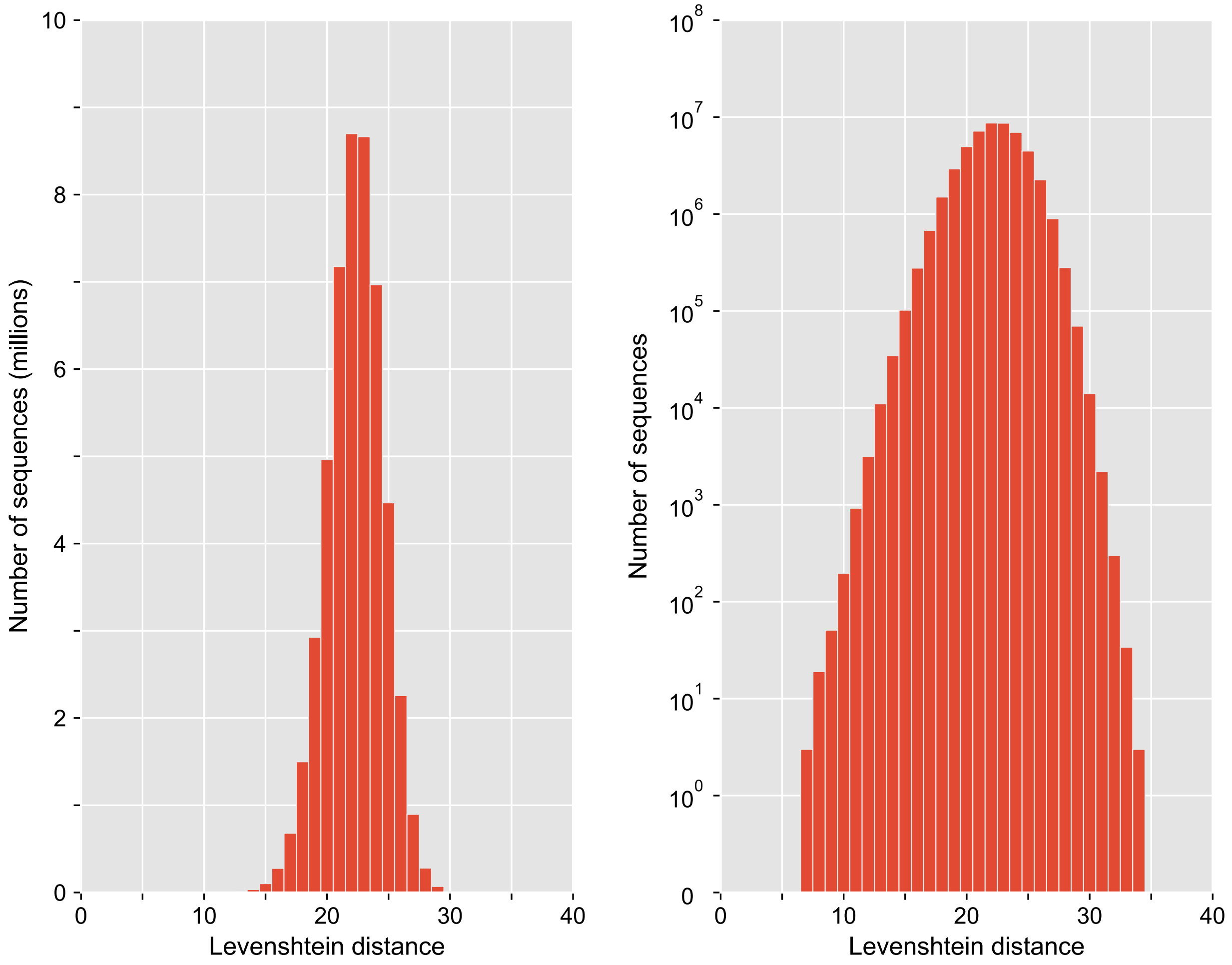

Supplementary Figure 13 | Pairwise Levenshtein distances for 10,000 CVAE-generated HFNAPs. Pairwise Levenshtein distances were calculated to identify areas with increased sequence similarity.

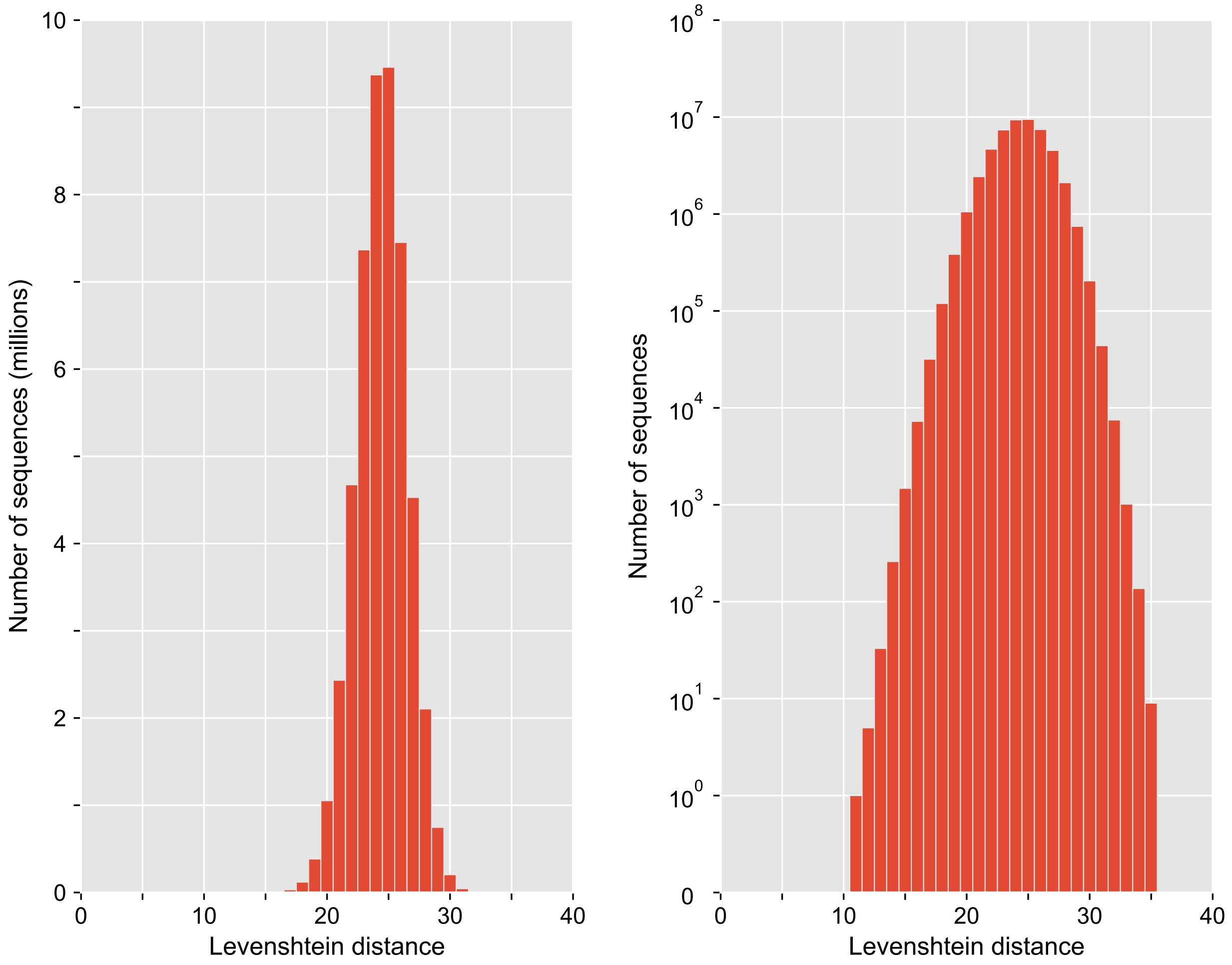

Supplementary Figure 14 | Pairwise Levenshtein distances for 10,000 truly random HFNAPs. Pairwise distance distributions for randomly sampled sequences using a 1/32 frequency for each codon are shown.

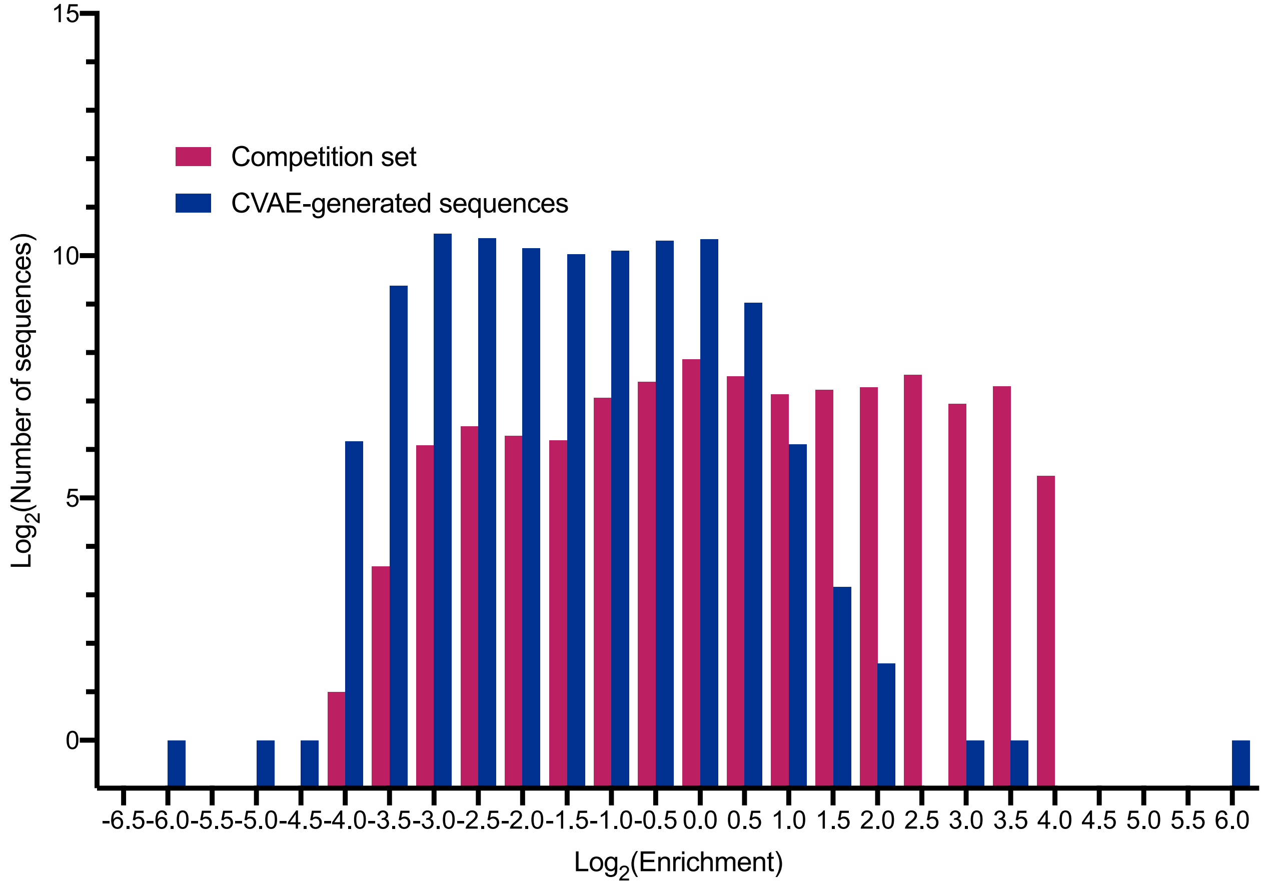

Supplementary Figure 15 | High-stringency selection of mixture of 10,000 CVAE-generated HFNAPs and 2,000 most highly enriched sequences from round 4 to round 8b of selection 2 (competition set). Enrichment values and the number of sequences are shown on $\log_2$ scale, with enrichment calculated by dividing the frequency for that sequence at the 60 min elution sample by the pre-selection frequency. For competition set HFNAPs, 1,250 of the 2,000 sequences were identified with $\log_2$(enrichments)>0. For CVAE-generated HFNAPs, 1,183 of the 10,000 sequences were identified with $\log_2$(enrichments)>0. Two CVAE-generated sequences were identified in the elution but not in the pre-selection sequencing reads, and so approximate enrichment values were assigned using the rarest identified sequence frequency in the pre-selection sequencing reads. 19 CVAE-generated sequences that were found in the pre-selection reads but not the elution reads, and 34 CVAE-generated sequences that were not found in either the pre-selection or elution reads, are omitted. A single competition selection sequence was not identified in either the pre-selection or elution reads and also omitted. X-axis bin values denote bin centers. For example, the $\log_2$(enrichment) = 0 bin includes all sequences in the range: $-0.25 \geq \log_2$(enrichment) > 0.25.

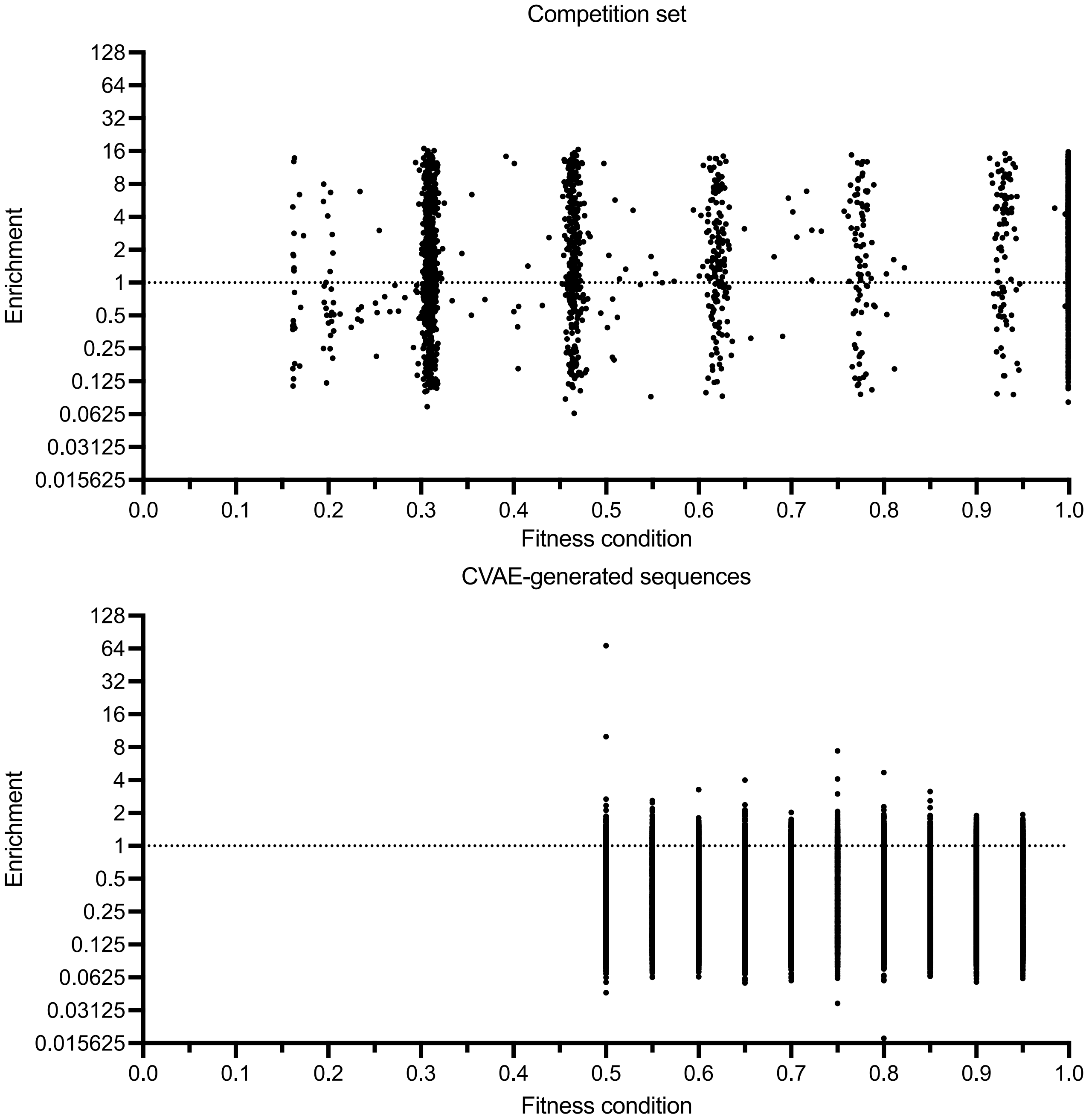

Supplementary Figure 16 | Enrichments from the competition selection. Enrichment values are calculated by dividing the frequency for that sequence at the 60 min elution sample by the pre-selection frequency. Two CVAE-generated sequences were identified in the elution but not in the pre-selection sequencing reads, and so approximate enrichment values were assigned using the rarest identified sequence frequency in the pre-selection sequencing reads. 53 CVAE-generated sequences were not found in the elution reads and therefore omitted. A single competition selection sequence was not identified in either the pre-selection or elution reads and also omitted. For the competition set, the x-axis fitness condition refers to the fitness condition assigned to a given sequence based on the selection 2 enrichments as described in the Methods. Fitness condition values are not derived from the competition selection enrichments. Correlation between the fitness condition and enrichment for the competition set was Pearson r = 0.16, $p_{two\text{-}tailed}=1.84\times10^{-13}$. For the CVAE-generated sequences, x-axis is the fitness condition used to generate the given sequence. Correlation between the fitness condition and enrichment for the CVAE-generated sequences was Pearson r = -0.022, $p_{two\text{-}tailed}=0.028$.

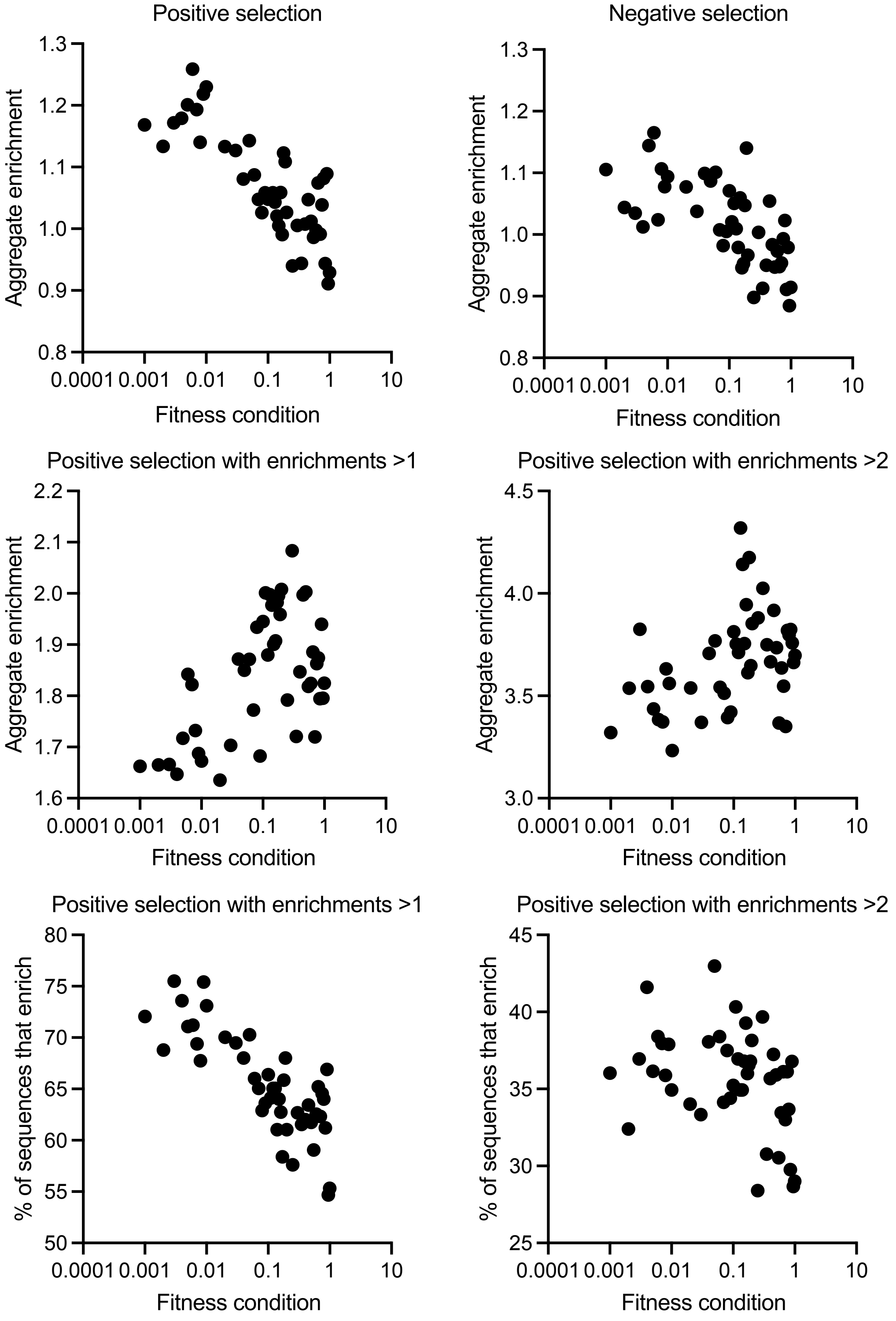

Supplementary Figure 17 | Aggregate enrichments from the 12,000 CVAE-generated HFNAP competition selection. 12,000 CVAE-generated HFNAPs were generated using fitness conditions ranging from 0.001 to 0.999 (see Methods). 250 sequences for each fitness ≤0.25, and 300 sequences for each fitness >0.25. The aggregate enrichment for each fitness condition was the sum of the 60 min elution frequencies divided by the sum of the pre-selection frequencies, for a given fitness condition. The positive selection exhibits negative correlation between the conditioned fitnesses and the aggregate enrichments (Pearson r = -0.627, $p_{\text{two-tailed}}=4.13\times10^{-6}$; Spearman r = -0.766, $p_{\text{two-tailed}}=8.44\times10^{-10}$). The negative selection exhibits negative correlation between the

conditioned fitnesses and the aggregate enrichments (Pearson r = -0.642, $p_{two\text{-}tailed}=2.00\times10^{-6}$; Spearman r = -0.708, $p_{two\text{-}tailed}=5.42\times10^{-8}$). When filtering the positive selection enrichments for enrichments >1, we observe a positive correlation between the conditioned fitnesses and the aggregate enrichments (Pearson r = 0.198, $p_{two\text{-}tailed}=0.192$; Spearman r = 0.449, $p_{two\text{-}tailed}=0.0020$). When filtering the positive selection enrichments for enrichments >2, we observe a positive correlation between the conditioned fitnesses and the aggregate enrichments (Pearson r = 0.164, $p_{two\text{-}tailed}=0.283$; Spearman r = 0.399, $p_{two\text{-}tailed}=0.0066$). The percentage of sequences that enrich in the positive selection with enrichment >1 and enrichment >2 are shown.

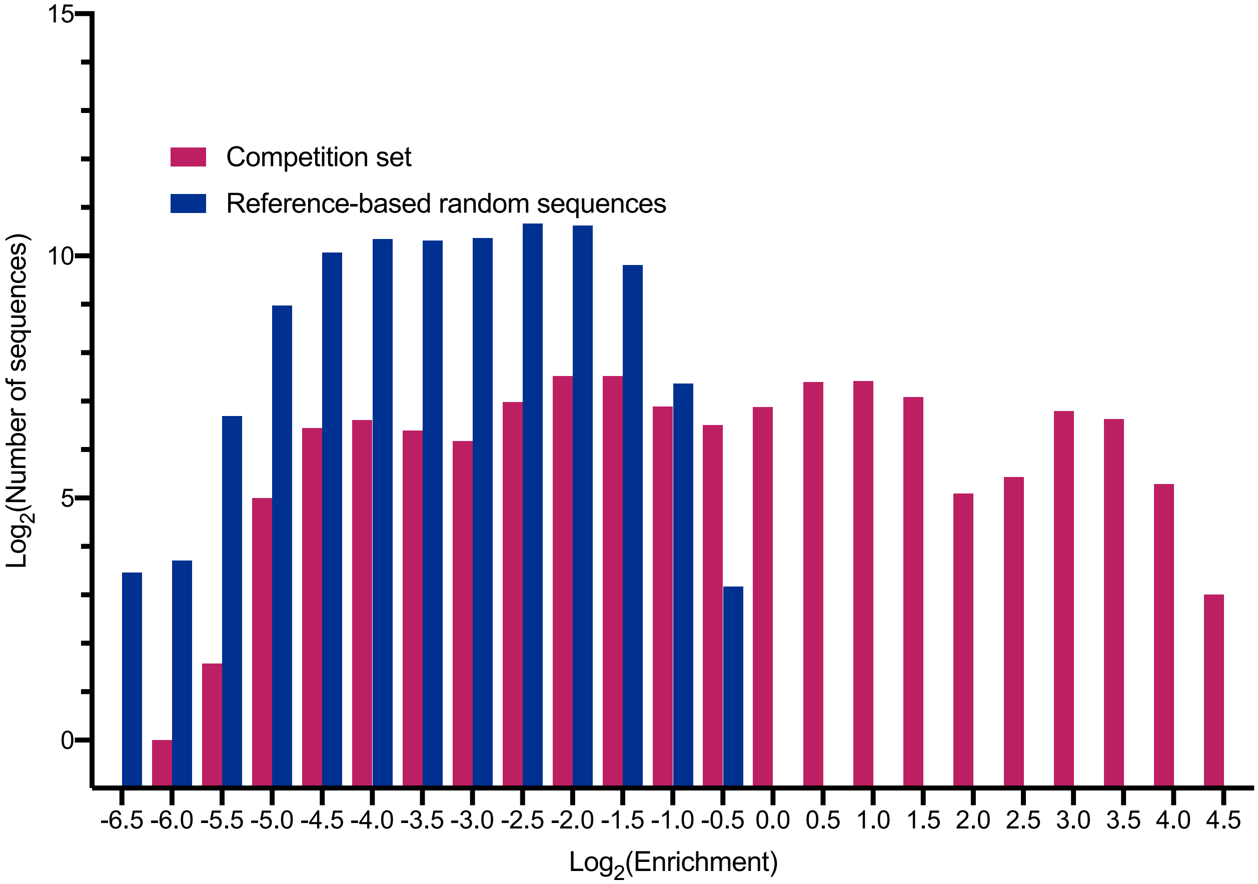

Supplementary Figure 18 | High-stringency selection of mixture of 10,000 reference-based random HFNAPs and 2,000 most highly enriched sequences from round 4 to round 8b of selection 2 (competition set). Enrichment values and the number of sequences are shown on a $\log_2$ scale, with enrichment calculated by dividing the frequency for that sequence at the 60 min elution sample by the pre-selection frequency. No reference-based random sequence enriched, as denoted by the lack of reference-based random sequences with $\log_2$(enrichment) ≥ 0. The highest observed enrichment value was 0.69 (linear scale value). X-axis bin values denote bin centers. For example, the $\log_2$(enrichment) = 0 bin includes all sequences in the range: -0.25 ≥ $\log_2$(enrichment) > 0.25.

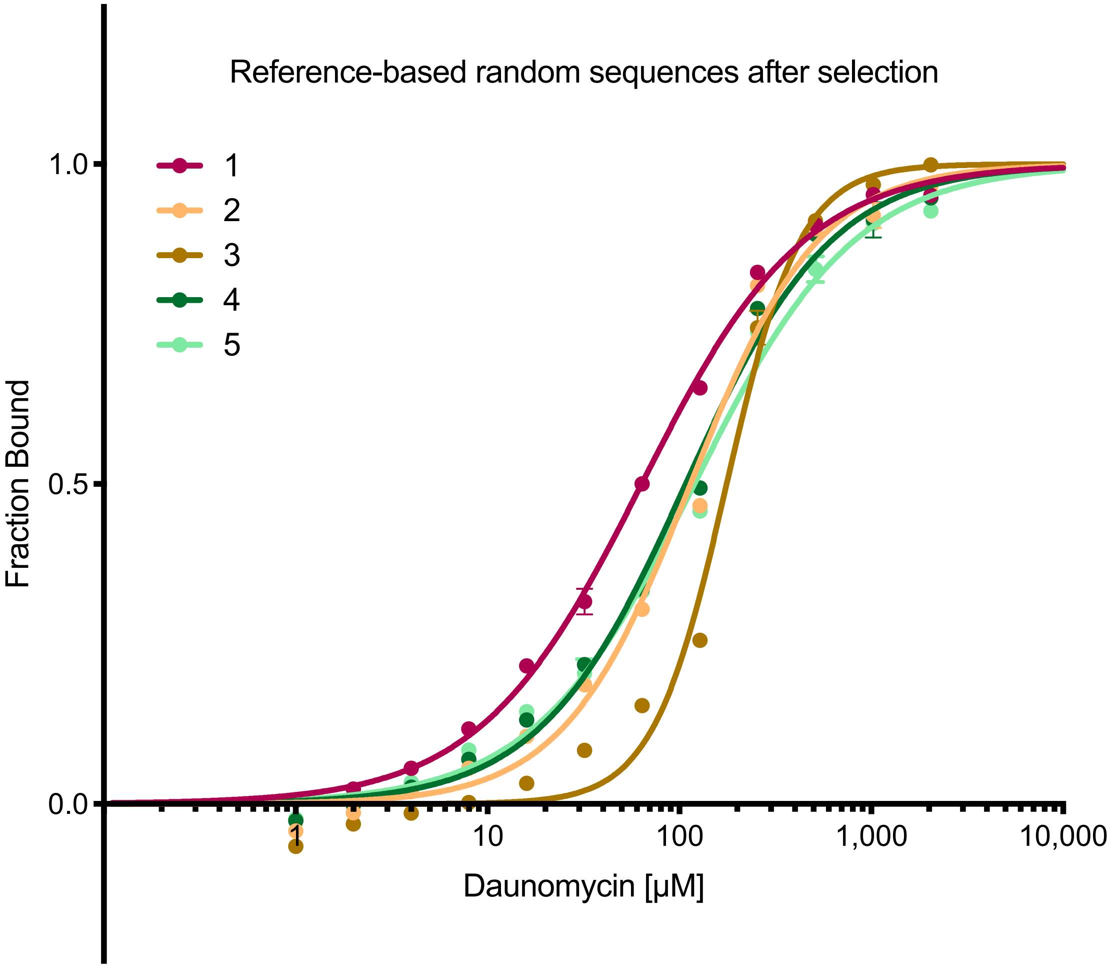

Sequence	K_d
Dm-reference-based-random-after-selection-1	63.6 ± 2.4 µM
Dm-reference-based-random-after-selection-2	114.4 ± 7.4 µM
Dm-reference-based-random-after-selection-3	176.5 ± 8.0 µM
Dm-reference-based-random-after-selection-4	107.9 ± 6.9 µM
Dm-reference-based-random-after-selection-5	119.7 ± 9.3 µM

Supplementary Figure 19 | MST characterization of the five most enriched sequences from the reference-based random set after selection. 10,000 reference-based random sequences and the top 2,000 sequences by fitness from selection 2 (competition set) were subject to HFNAP translation and one round of high-stringency selection. The top five most enriched sequences from the reference-based random set were translated and characterized by MST. Sequences bound with K_d >10 µM. The mean and SEM of n = 3 independent replicates are shown. Error bars for some values are too small to extend beyond the data point.

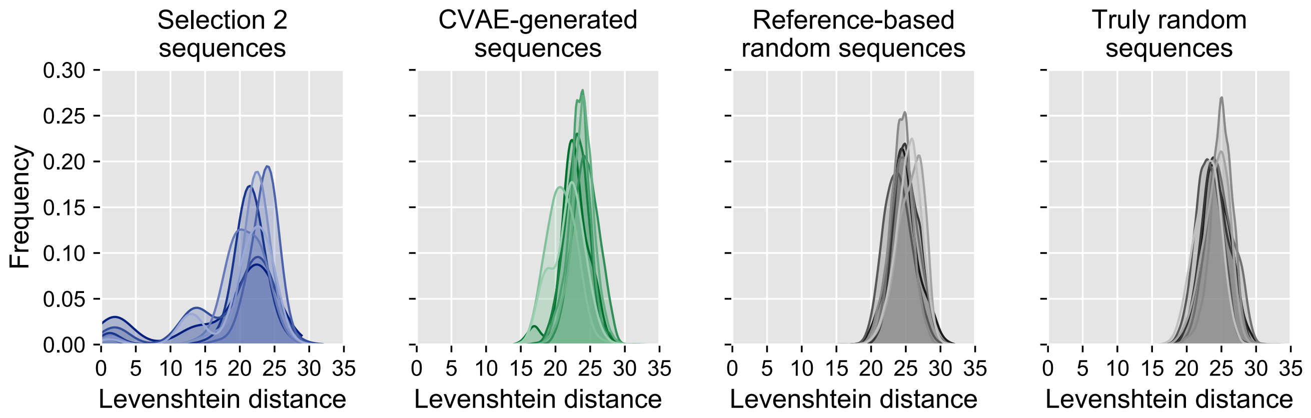

Supplementary Figure 20 | Comparison of the Levenshtein distances from the 3,072-member reference set to the top seven sequences from each of the four groups. Reference-based random sequences are HFNAP sequences randomly sampled using the same building block frequency as the reference set. Truly random sequences are HFNAP sequences randomly sampled using a uniform building block frequency distribution of 1/32. Selection 2 daunomycin-binding HFNAPs include Dm-HS-1, 2, 3, 6, 7, 8, and 10. Dm-HS-4 and Dm-HS-5 were omitted for sequence similarity to Dm-HS-1, and Dm-HS-9 was omitted for sequence similarity to Dm-HS-3.

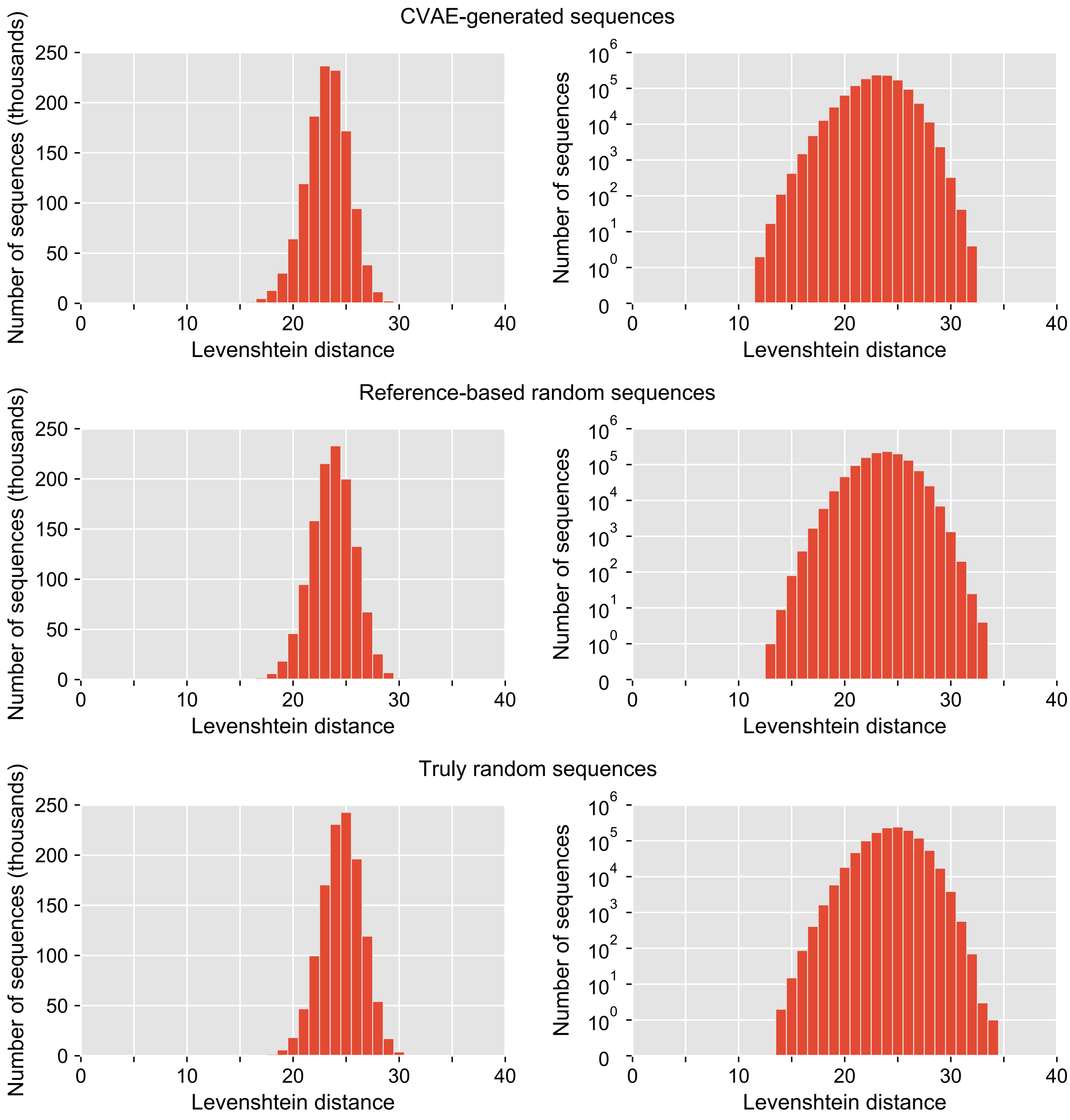

Supplementary Figure 21 | Pairwise Levenshtein distances between seven sequences and 172,545-member training set. Pairwise distances for the seven CVAE-generated daunomycin-binding HFNAPs, seven reference-based random sequences, and seven truly random sequences were calculated with respect to the training set, and are shown in aggregate.

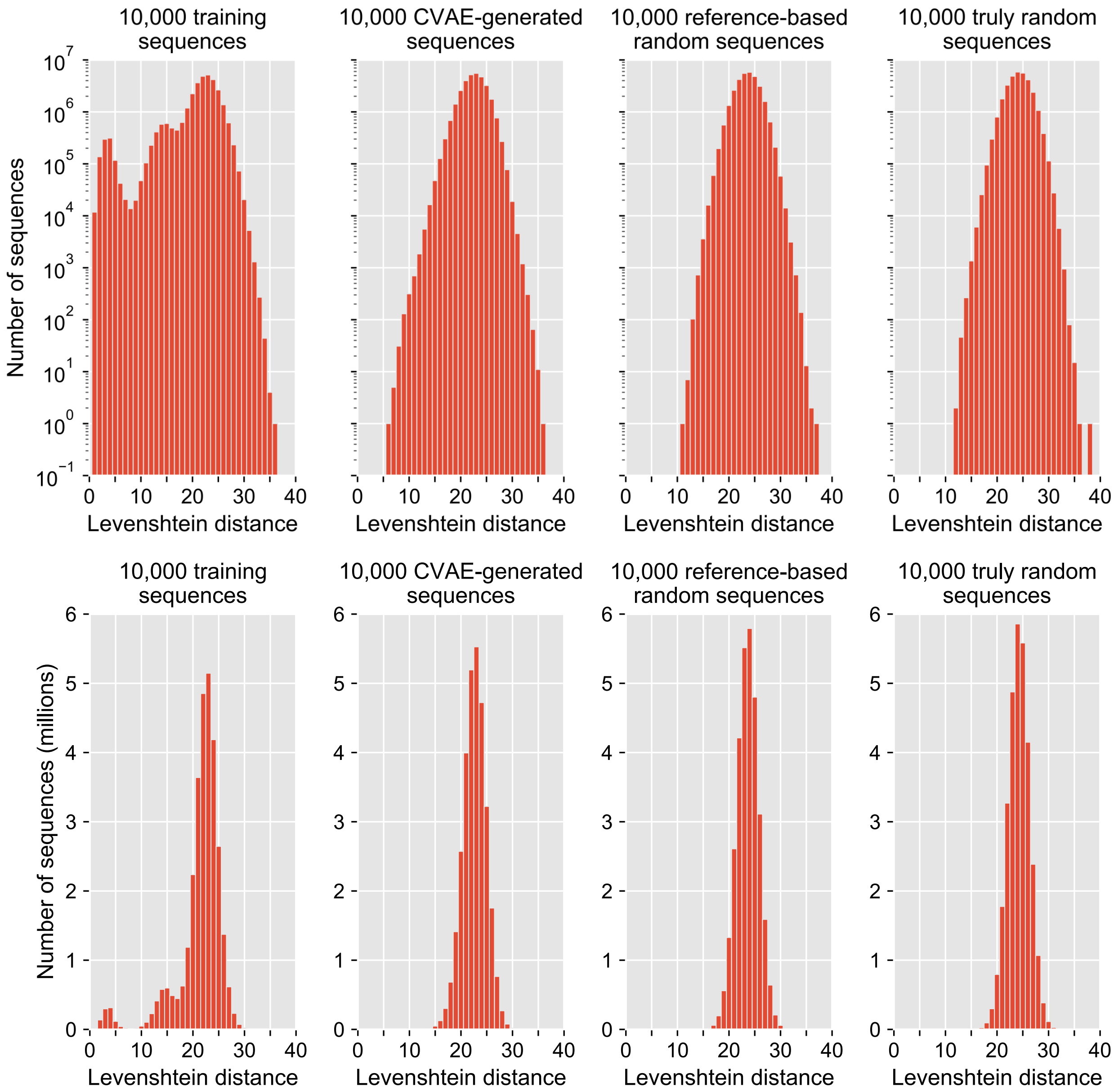

Supplementary Figure 22 | Pairwise Levenshtein distances from reference set to 10,000-member libraries. Pairwise Levenshtein distances are calculated and plotted in aggregate.

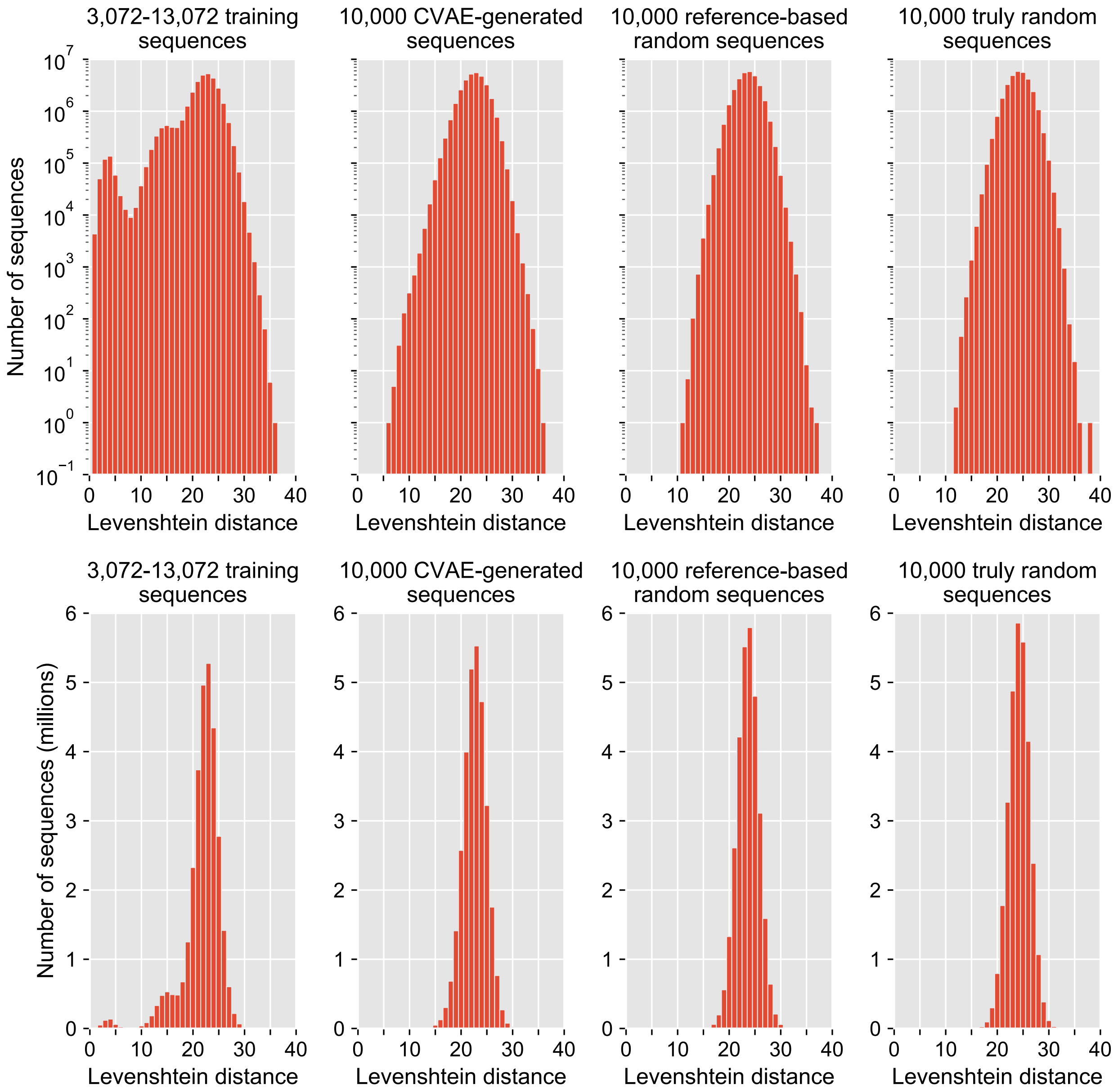

Supplementary Figure 23 | Pairwise Levenshtein distances from reference set to 10,000-member libraries. The reference set consists of the top 3,072 HFNAP sequences with the highest fitness in selection 2. Top 3,072 to 13,072 training set sequences by fitness are used to remove direct overlaps present in the reference set to the top training set sequences. Pairwise Levenshtein distances are calculated and plotted in aggregate.

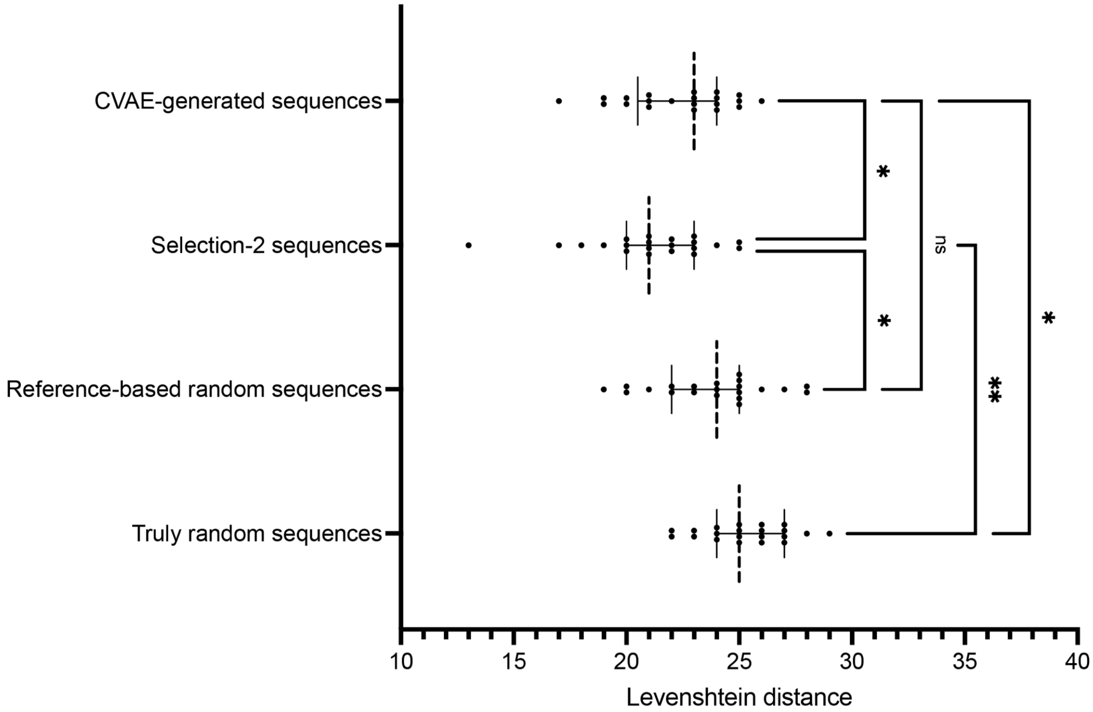

A	B	pseudo F statistic	PERMANOVA p value	Permdisp p value
CVAE-generated sequences	Selection 2 sequences	1.531	0.0494	0.211
CVAE-generated sequences	Reference-based random sequences	1.001	0.485	0.229
CVAE-generated sequences	Truly random sequences	1.532	0.0249	0.00639
Selection 2 sequences	Reference-based random sequences	1.674	0.0234	0.0458
Selection 2 sequences	Truly random sequences	1.957	0.00159	0.00170

Supplementary Figure 24 | Distance distributions for pairwise Levenshtein distances for seven validated CVAE-generated HFNAP sequences, seven validated selection-2 HFNAP sequences, seven reference-based random HFNAP sequences, and seven truly random HFNAP sequences. Median and quartile distances are shown. Distance distributions are compared using PERMANOVA (see Methods). P-values are one-tailed.

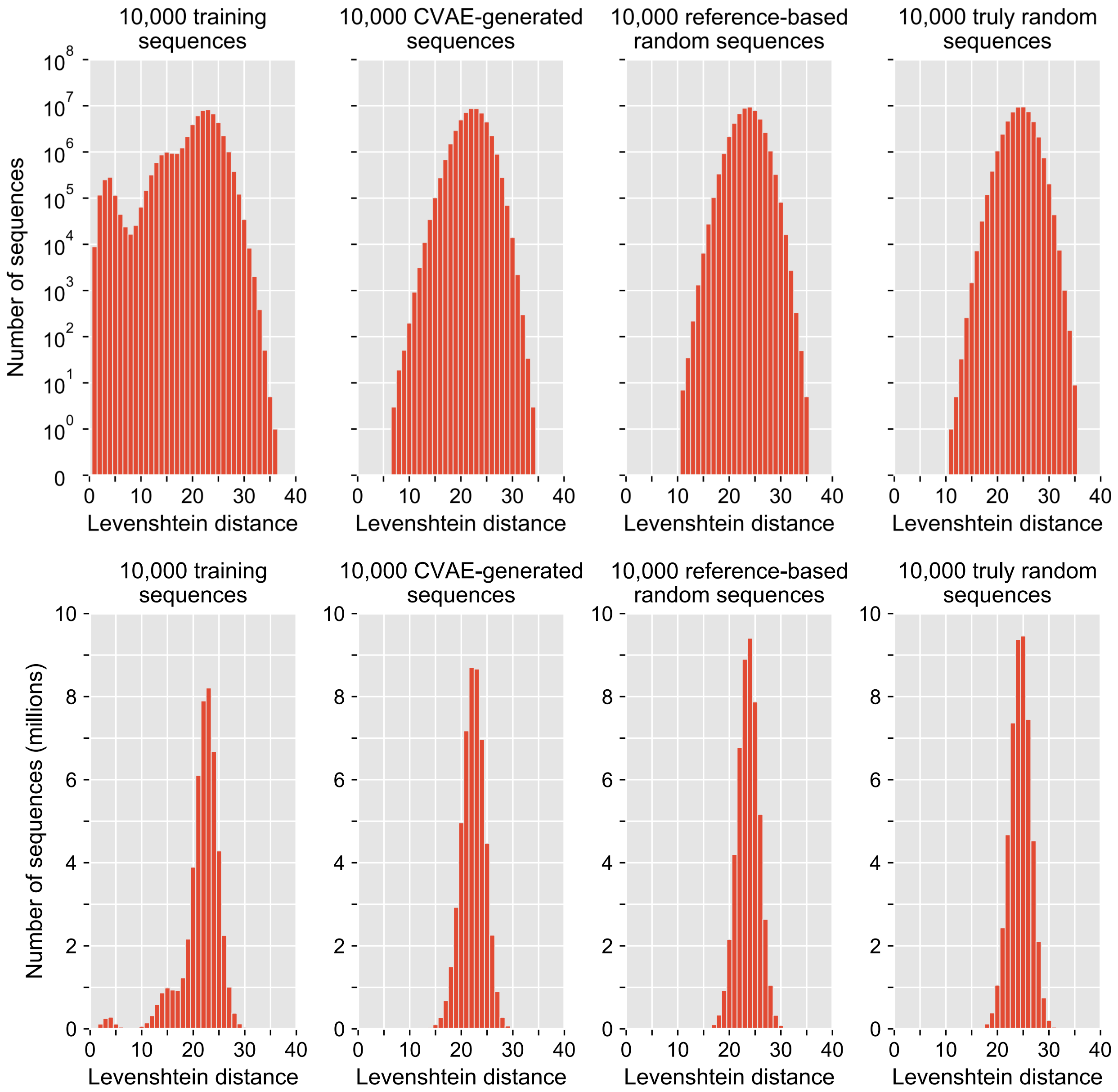

Supplementary Figure 25 | Pairwise Levenshtein distances amongst 10,000-member libraries. Pairwise Levenshtein distances are calculated between every member of the given library and shown in aggregate.

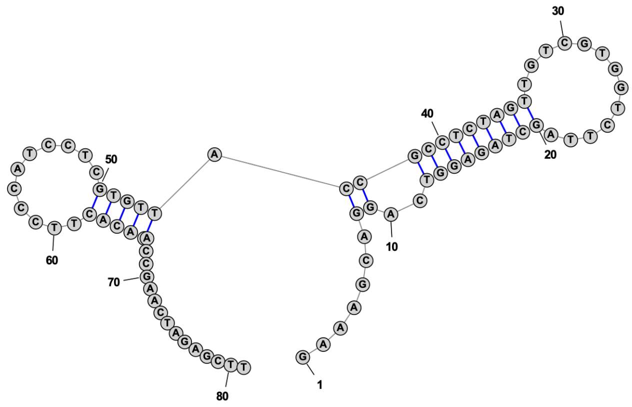

Dm-HS-1, MFE

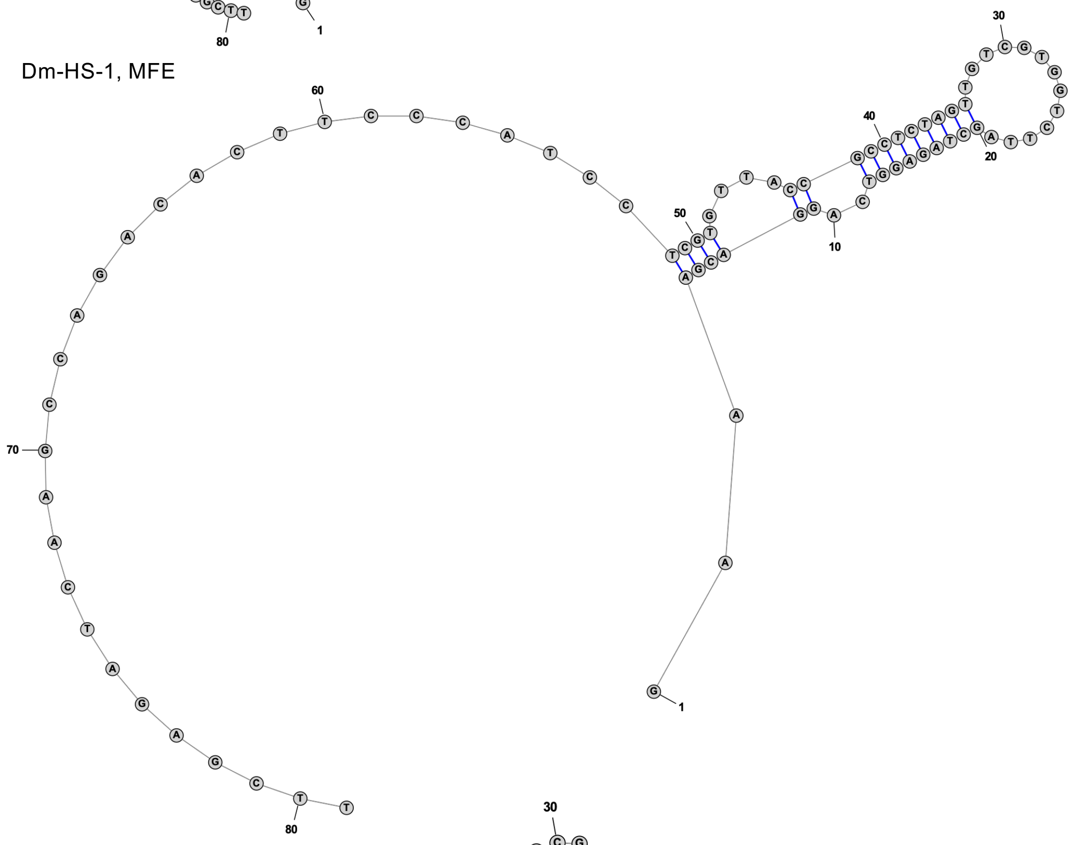

Dm-HS-1, structure 2

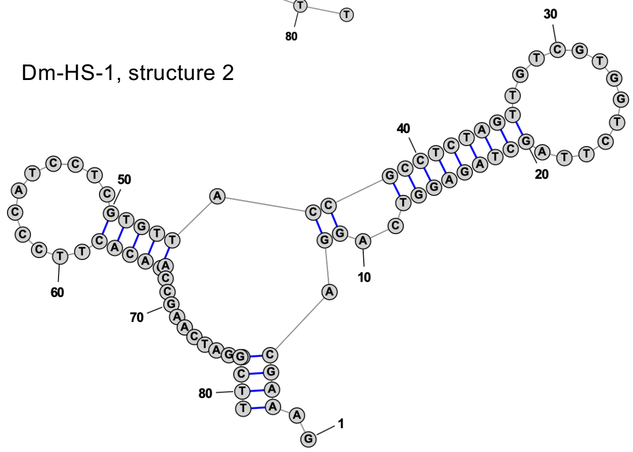

Dm-HS-1, structure 3

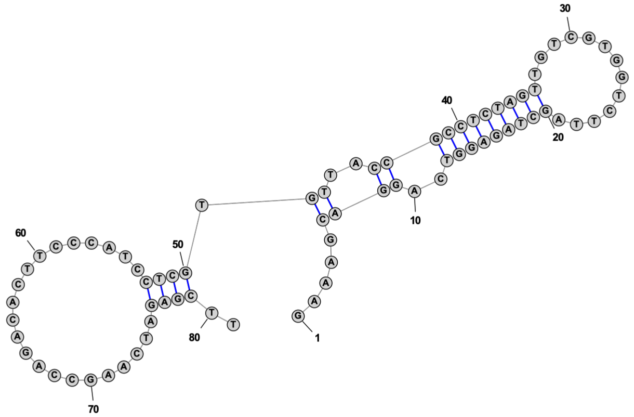

Dm-HS-1, structure 4

Supplementary Figure 26 | Predicted secondary structures for Dm-HS-1 from RNAstructure. Sequence was folded using the Fold program, using default DNA parameters.

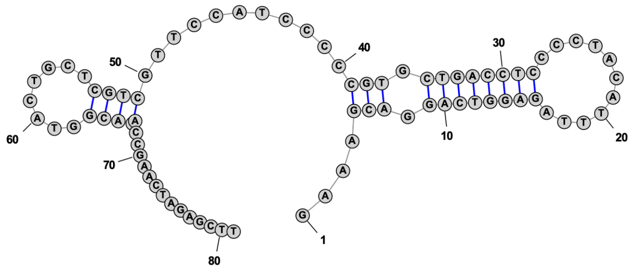

Dm-CCS-3, structure 2

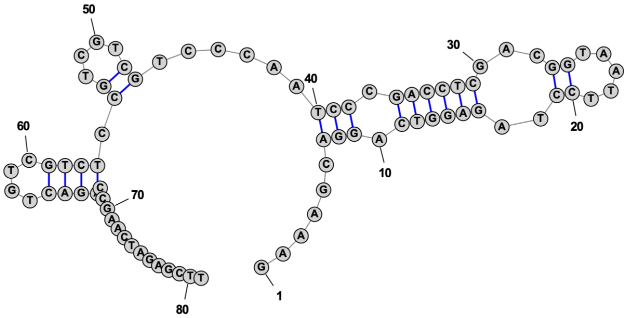

Dm-CCS-2, structure 3

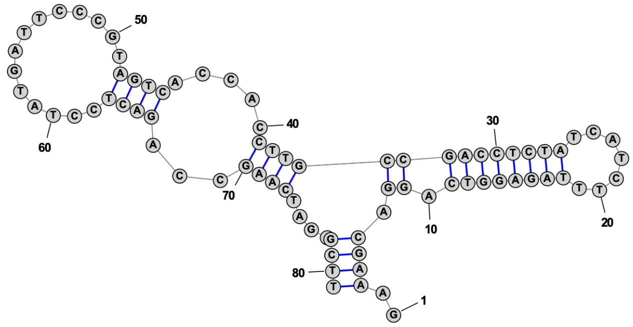

Dm-CCS-5, MFE

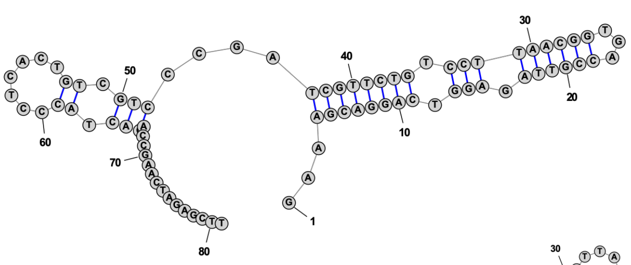

Dm-CCS-6, structure 3

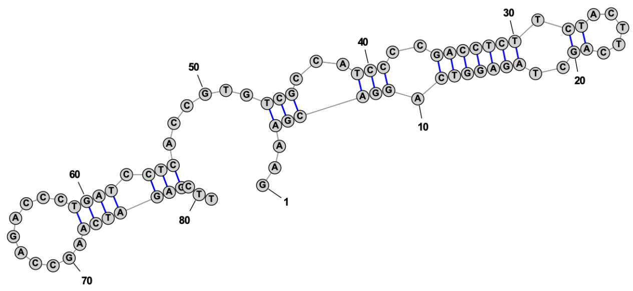

Dm-CCS-7, MFE

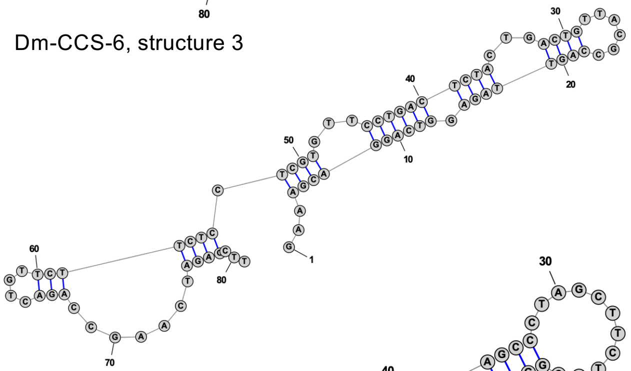

Dm-CCS-8, MFE

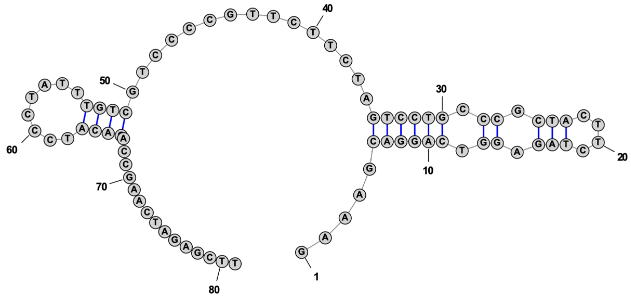

Dm-CCS-10, MFE

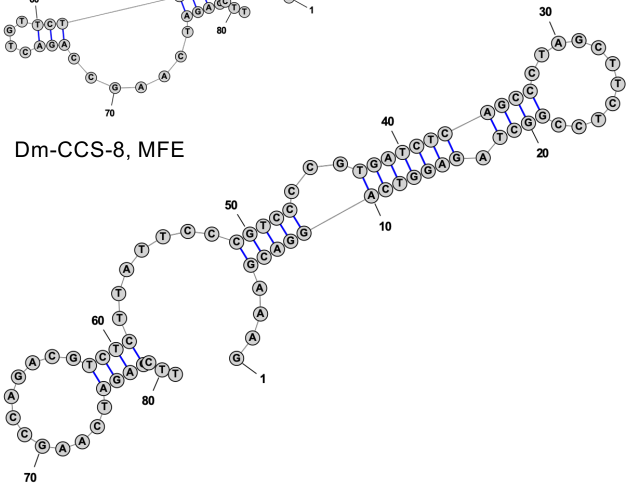

Dm-CCS-11, structure 2

Supplementary Figure 27 | Predicted secondary structures for CVAE-generated sequences after competition selection with qualitative structural similarities to Dm-HS-1 predicted structures. Dm-CCS-3 structure 2 is 24 base-pair-edit distance away from the Dm-HS-1 MFE (minimum free energy) structure, and the Dm-CCS-5 MFE structure was 30 base-pair-edit distance away from Dm-HS-1 structure 3.

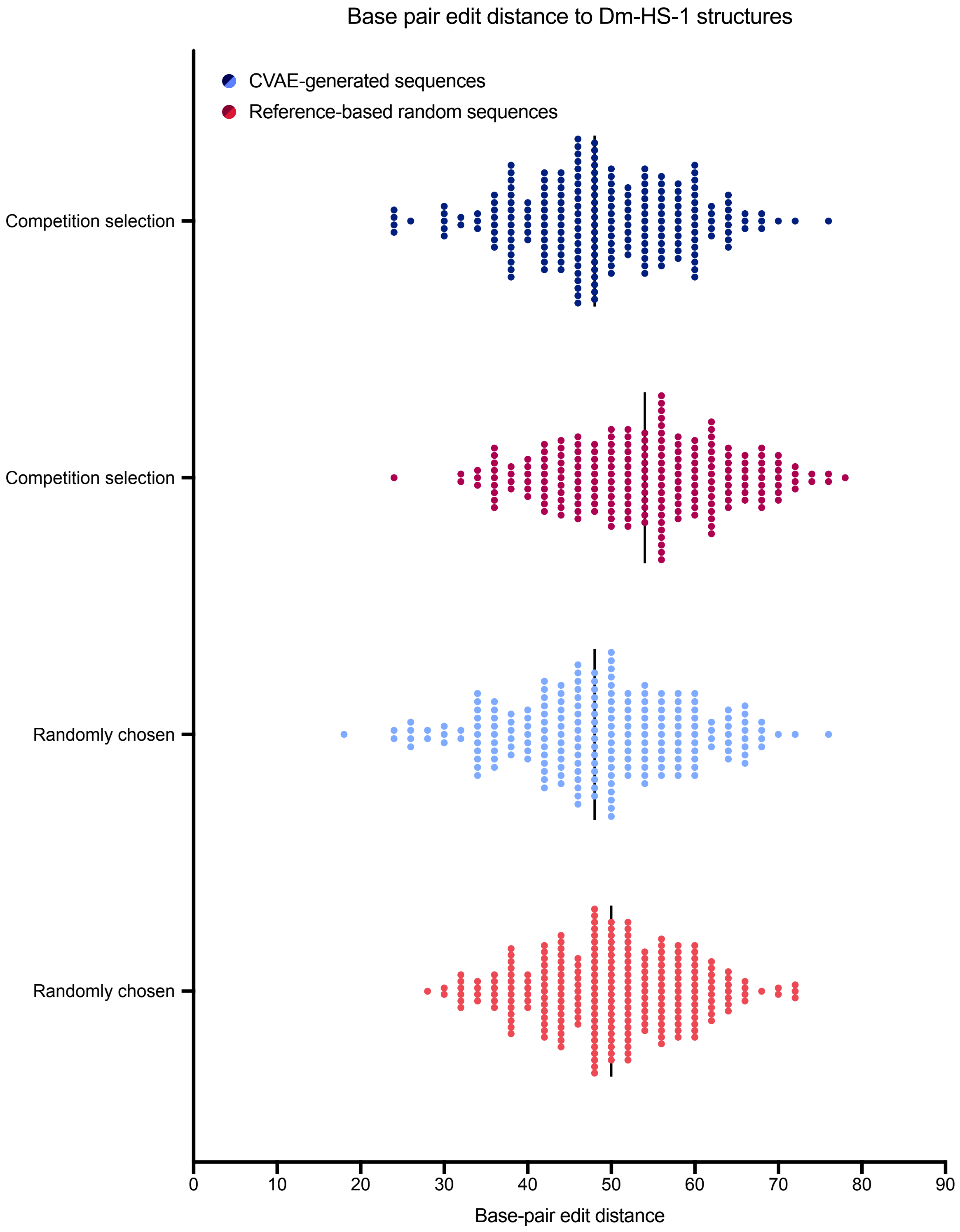

Supplementary Figure 28 | Base-pair-edit distance between Dm-HS-1 structures and predicted secondary structures from CVAE-generated sequences and reference-based random sequences. Distances to Dm-HS-1 structures were calculated from 25 CVAE-generated sequences from the competition selection, 25 reference-based random sequences from the competition selection, 25 randomly chosen CVAE-generated sequences, and 25 randomly chosen reference-based random sequences. Lowest observed distances were 24, 24, 18, and 28, respectively. Median values are indicated using solid black lines.

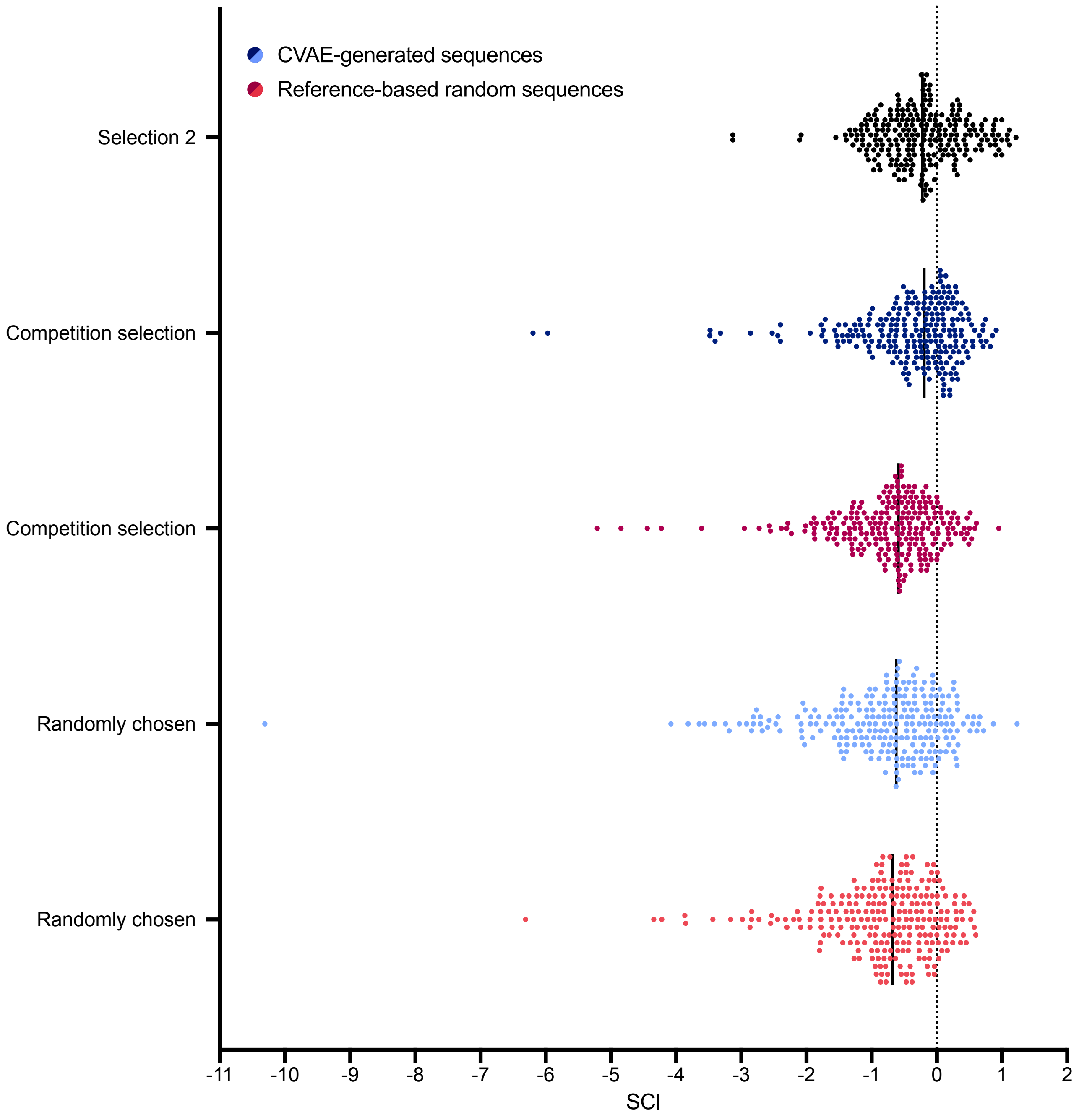

Supplementary Figure 29 | Distribution of SCI scores for CVAE-generated sequences, reference-based random sequences, and selection-2 sequences to Dm-HS-1-10. SCI scores were calculated as described in the methods, and positive SCI scores indicate structural similarity to the Dm-HS sequence being compared. CVAE-generated sequences achieve increased numbers of positive SCI scores and larger SCI scores. An SCI value of -21.6 for a randomly chosen CVAE-generated sequence to Dm-HS-7 was omitted for clarity. Median values are indicated using solid black lines.

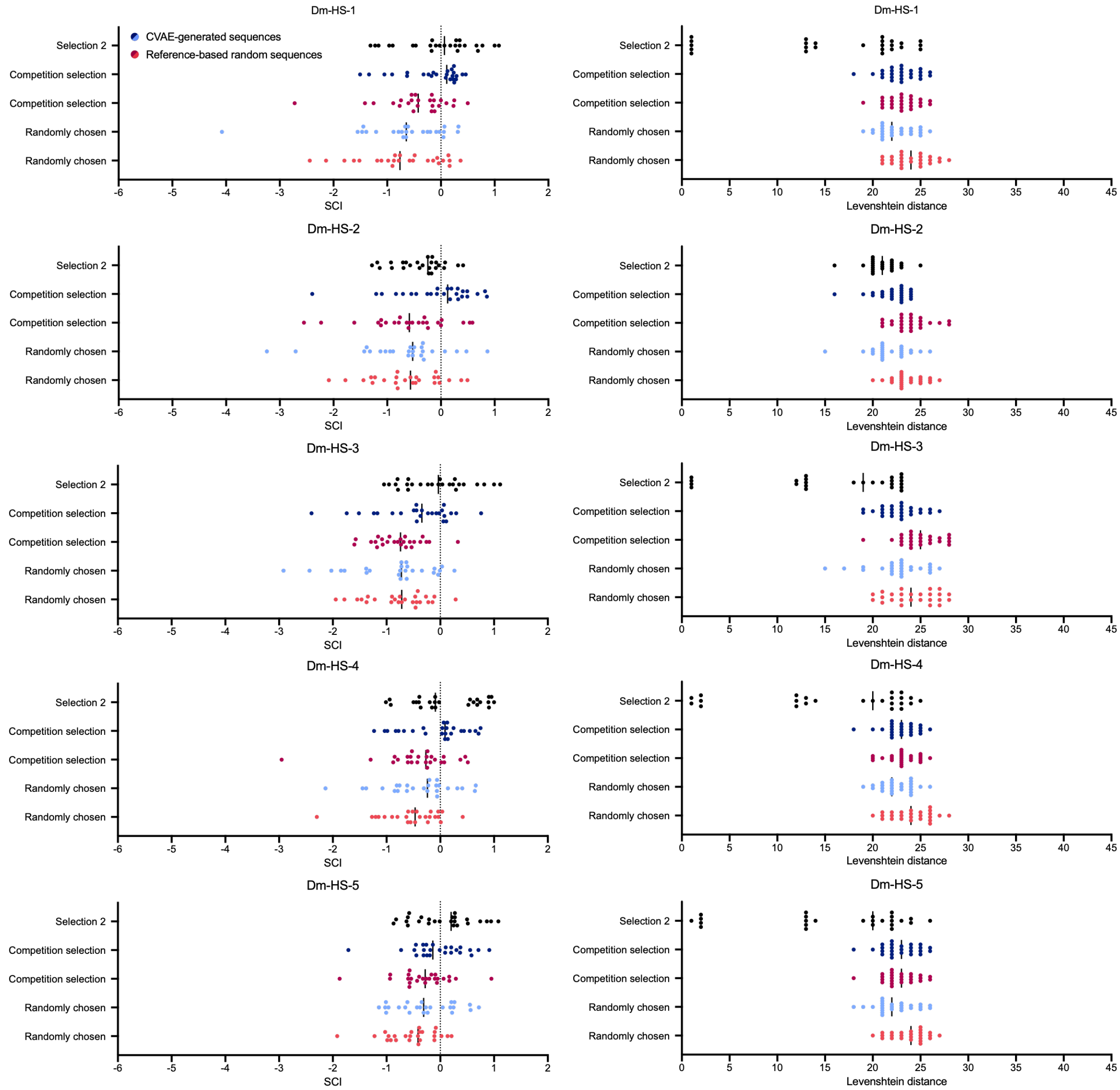

Supplementary Figure 30 | Distribution of SCI scores and Levenshtein distances for CVAE-generated sequences, reference-based random sequences, and selection-2 sequences to Dm-HS-1-5. SCI scores were calculated as described in the methods, and positive SCI scores indicate structural similarity to the Dm-HS sequence being compared. For sequences from the competition selection, CVAE-generated sequences achieve increased numbers of positive SCI scores and larger SCI scores compared to reference-based random sequences. Randomly chosen CVAE-generated sequences demonstrate increased structure conservation to Dm-HS-4-5 compared to the structure conservation between randomly chosen reference-based random sequences and Dm-HS-4-5. Median values are indicated using solid black lines.

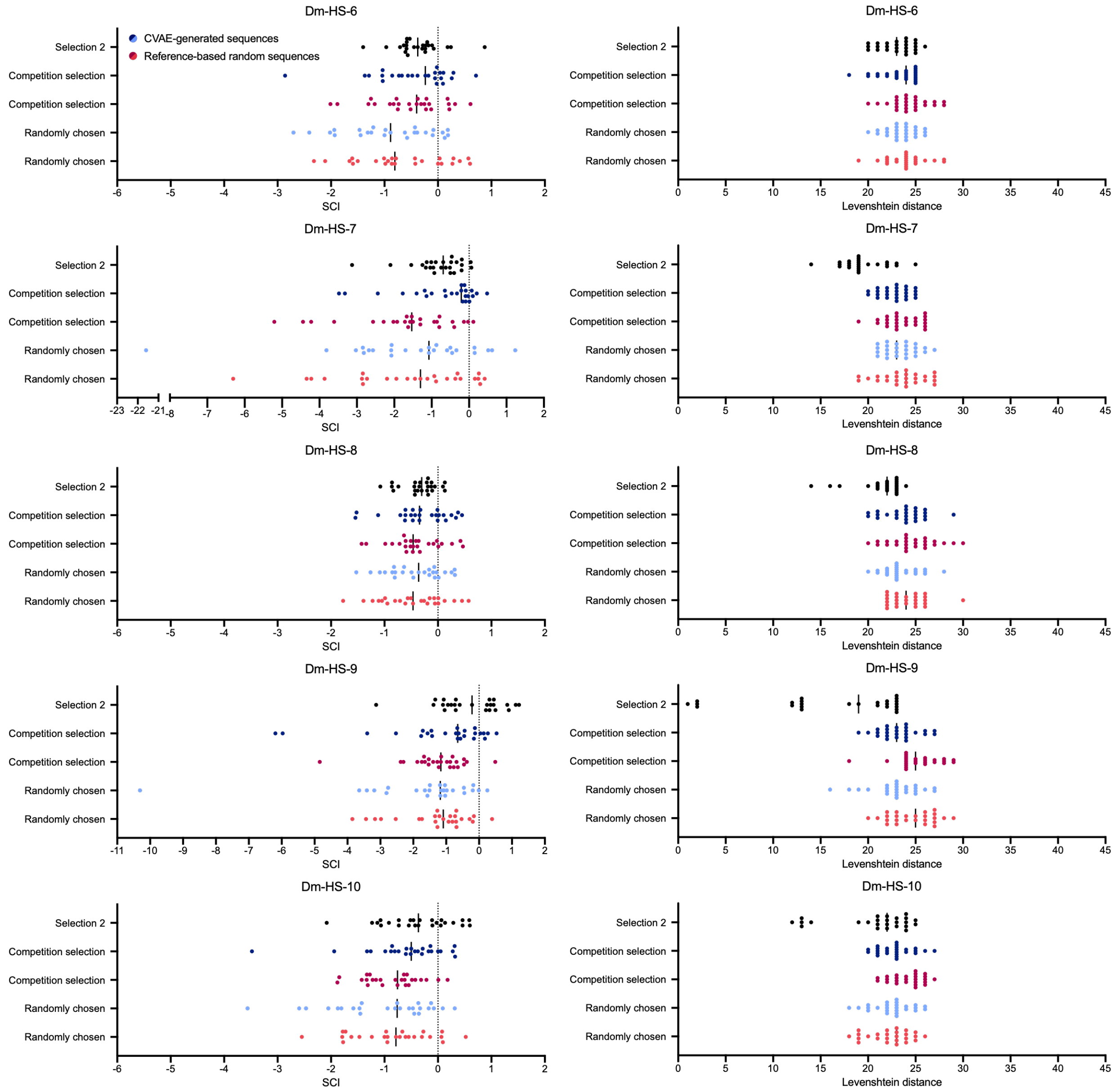

Supplementary Figure 31 | Distribution of SCI scores and Levenshtein distances for CVAE-generated sequences, reference-based random sequences, and selection-2 sequences to Dm-HS-6-10. SCI scores were calculated as described in the methods, and positive SCI scores indicate structural similarity to the Dm-HS sequence being compared. Median values are indicated using solid black lines.

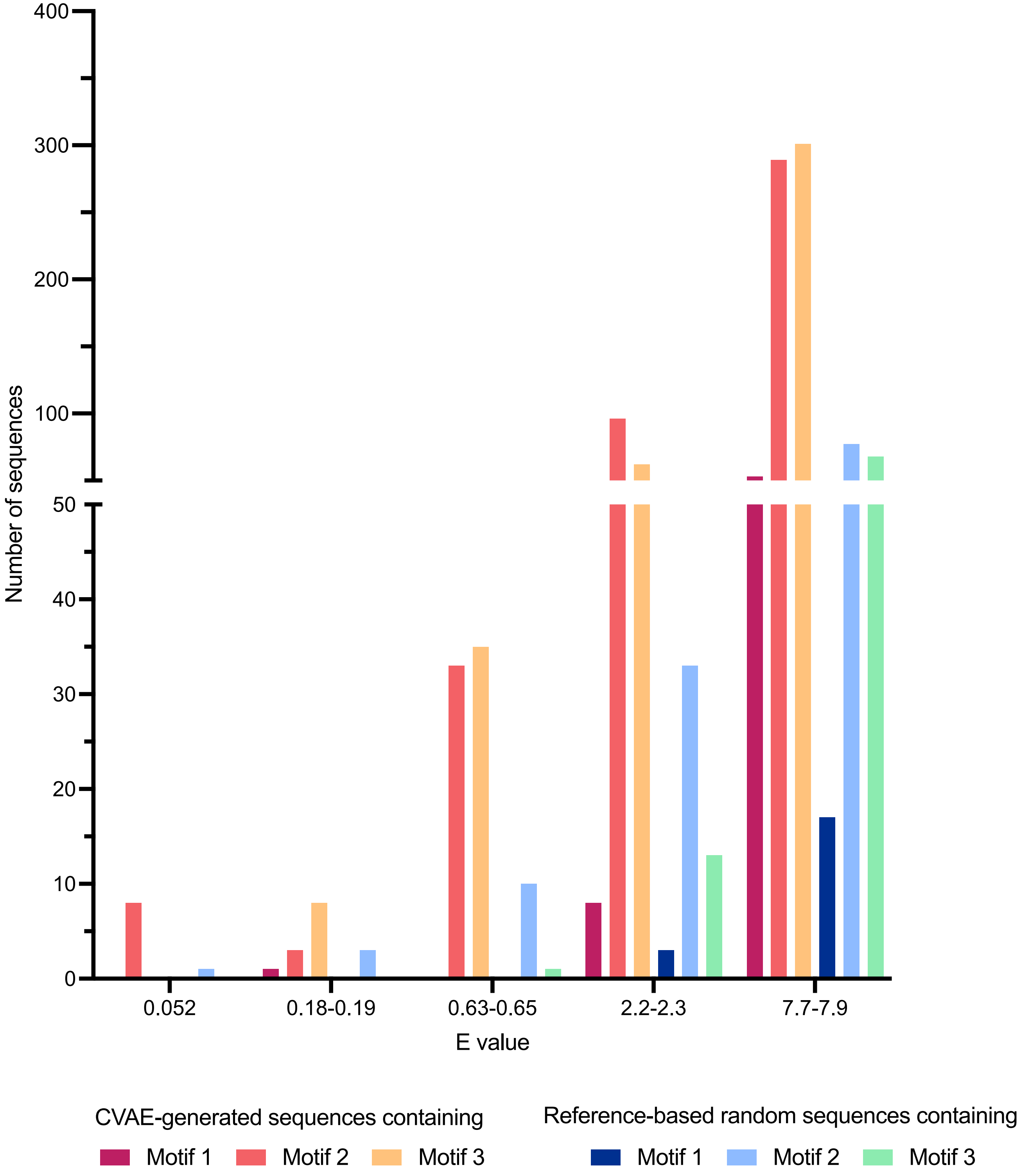

Supplementary Figure 32 | Number of sequences containing motifs 1-3 for a given E value in 10,000 CVAE-generated sequences and 10,000 reference-based random sequences. Motifs are overrepresented in CVAE-generated sequences compared to reference-based random sequences.

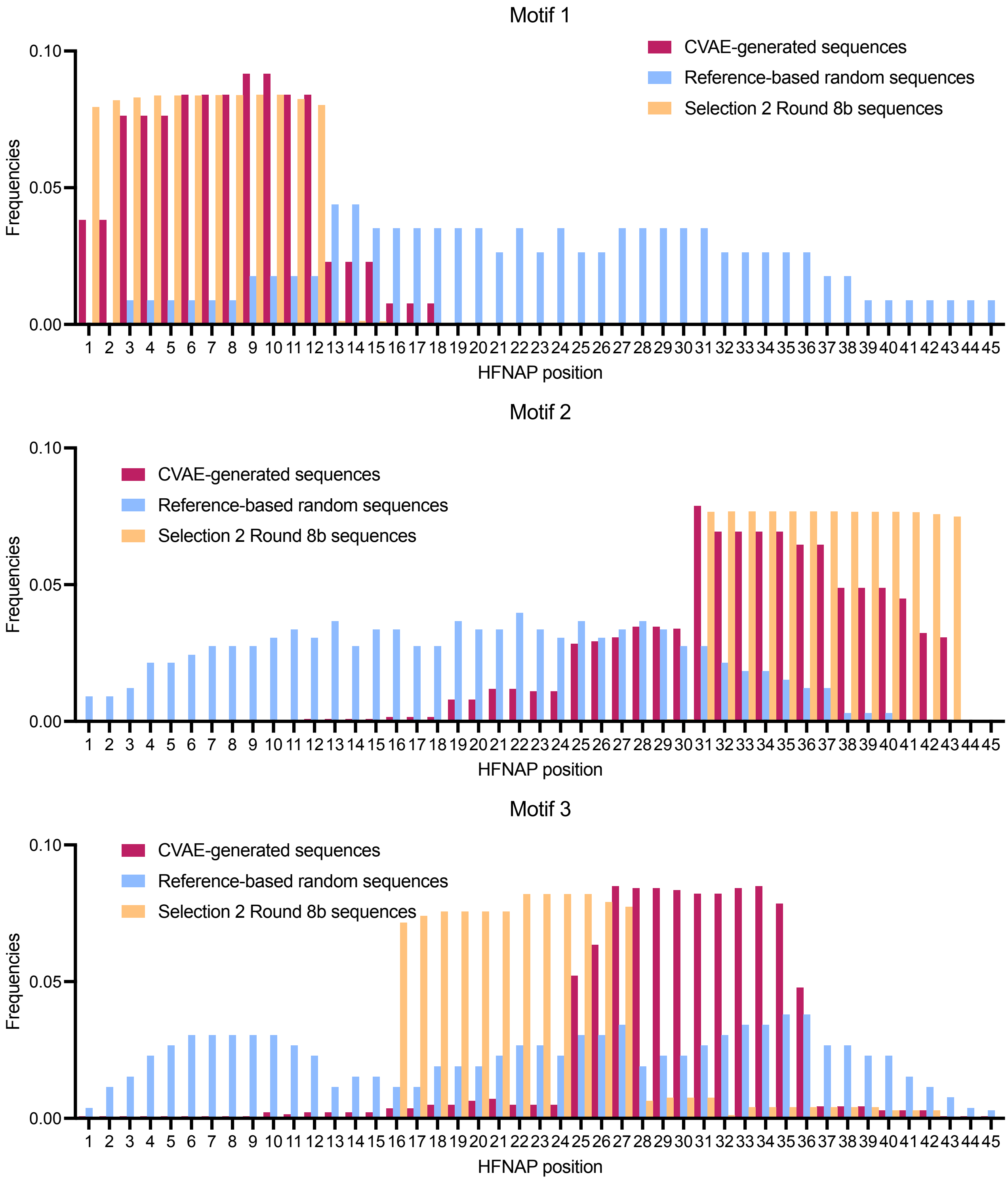

Supplementary Figure 33 | HFNAP positioning of sequence-structural motifs identified by AptaTrace in CVAE-generated sequences, reference-based random sequences, and selection 2 round 8b sequences. Motifs were identified by AptaTrace, followed by BLAST to discover sequences containing the motif (see Methods).

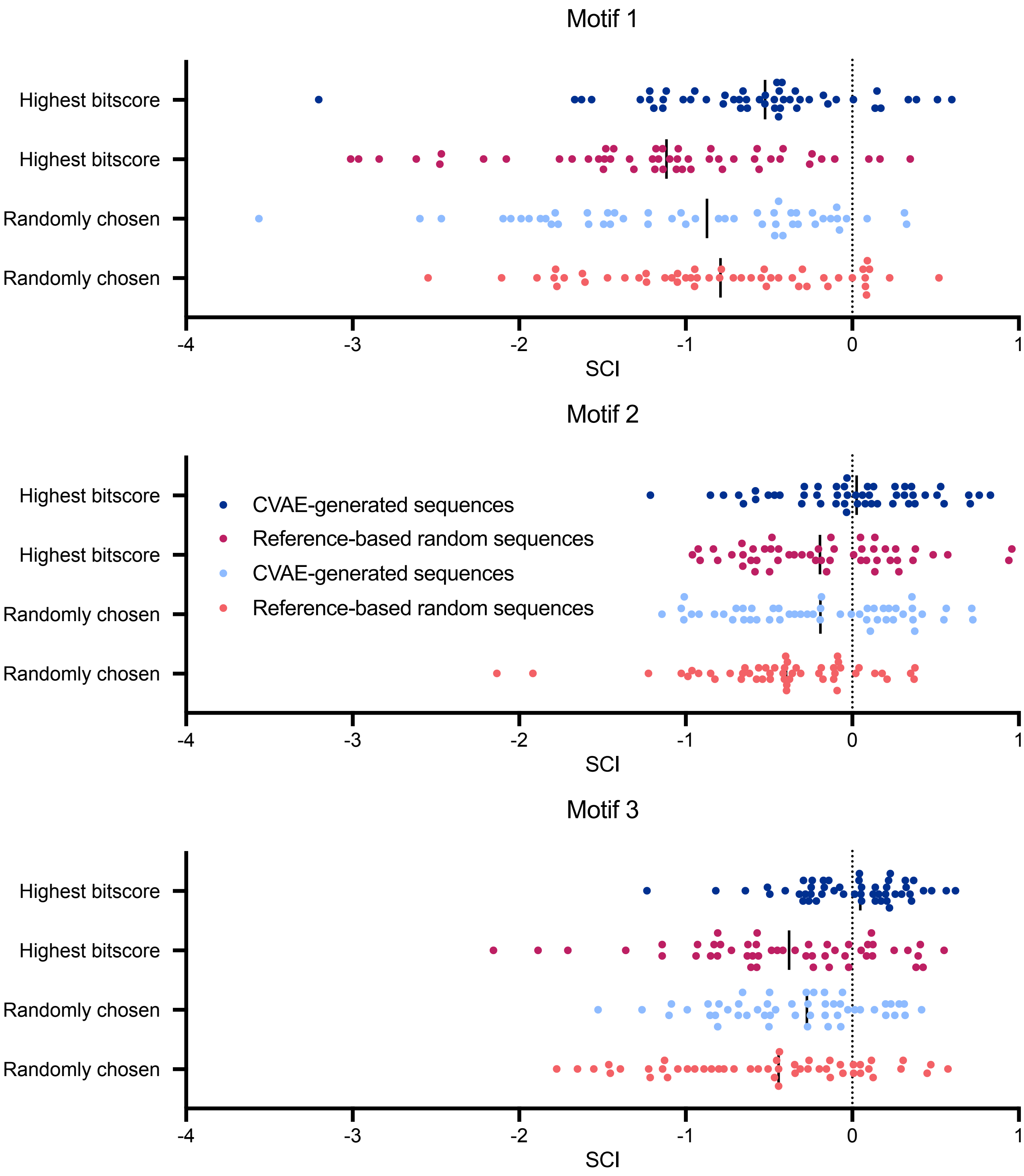

Supplementary Figure 34 | Distribution of SCI scores for CVAE-generated sequences and reference-based random sequences to Dm-HS-10, -5, and -8 for motifs 1-3, respectively. SCI scores were calculated as described in the Methods, and positive SCI scores indicate structural similarity to the Dm-HS sequence being compared. Median values are indicated using solid black lines.

Supplementary Note 1 | Oligonucleotides used in selections, HFNAP preparation, and HTS.

Oligonucleotides for daunomcyin affinity selection

Name	Sequence
Biotinylated DX template library	/52-Bio/iSp18/AAGCTCTAGTTCGGTCTG NNY NNY NNY NNY NNY NNY NNY NNY NNY NNY NNY NNY NNY NNY NNY ATCTCCAGTCCTGCTTTC
BtBt ExtPrimerD	/52-Bio/iSp18/AAGCTCTAGTTCGGTCTG
ExtPrimer D	AAGCTCTAGTTCGGTCTG
pp1D	/5Phos/CAGACCGAACTAGAGCTT
dc pp1D	/5Phos/CAGACCGAACTAGAGCTT/3ddC/
pp2X	GAAAGCAGGACTGGAGAT
Seq Adapt D1	ACACTCTTTCCCTACACGACGCTCTTCCGATCT N AAGCTCTAGTTCGGTCTG
Seq Adapt D2	ACACTCTTTCCCTACACGACGCTCTTCCGATCT NN AAGCTCTAGTTCGGTCTG
Seq Adapt D3	ACACTCTTTCCCTACACGACGCTCTTCCGATCT NNN AAGCTCTAGTTCGGTCTG
Seq Adapt D4	ACACTCTTTCCCTACACGACGCTCTTCCGATCT NNNN AAGCTCTAGTTCGGTCTG
Seq Adapt X1	TGGAGTTCAGACGTGTGCTCTTCCGATCT N GAAAGCAGGACTGGAGAT
Seq Adapt X2	TGGAGTTCAGACGTGTGCTCTTCCGATCT NN GAAAGCAGGACTGGAGAT
Seq Adapt X3	TGGAGTTCAGACGTGTGCTCTTCCGATCT NNN GAAAGCAGGACTGGAGAT
Seq Adapt X4	TGGAGTTCAGACGTGTGCTCTTCCGATCT NNNN GAAAGCAGGACTGGAGAT
IlluminaAdapterFwd	AATGATACGGCGACCACCGAGATCTACAC [8-base barcode] ACACTCTTTCCCTACACGAC
IlluminaAdapterRev	CAAGCAGAAGACGGCATACGAGAT [8-base barcode] GTGACTGGAGTTCAGACGTGTGCT

Oligonucleotides for synthesis of individual hits for characterization

Name	Sequence
pp1D	/5Phos/CAGACCGAACTAGAGCTT
dc pp1D	/5Phos/CAGACCGAACTAGAGCTT/3ddC/
pp2X	GAAAGCAGGACTGGAGAT
pp2x Cy5	/5Cy5//iSp18/GA AAG CAG GAC TGG AGA T
Dm-LS-1 template	/52-Bio/iSp18/AAGCTCTAGTTCGGTCTG TAAGGGGAAGTAGGGTAAAAGGGACTAGGGATAAAAGCGGCGCGG ATCTCCAGTCCTGCTTTC
Dm-LS-2 template	/52-Bio/iSp18/AAGCTCTAGTTCGGTCTG CAAGGAGGGATACGGGTAGCGGGGACAGGGATAAAAGCGGCGCGG ATCTCCAGTCCTGCTTTC

Dm-LS-3 template	/52-Bio/iSp18/AAGCTCTAGTTCGGTCTG<br>AGGTAGCAGTGGGCGGTGGGACGGCAAGGAGGAGTAGCGATGCGA<br>ATCTCCAGTCCTGCTTTC
Dm-LS-4 template	/52-Bio/iSp18/AAGCTCTAGTTCGGTCTG<br>TAAGGGGAAGTAGGGTAAGAGGGACTAGGGATAAAAGCGGCGCGG<br>ATCTCCAGTCCTGCTTTC
Dm-HS-1 template	/52-Bio//iSp18/AAGCTCTAGTTCGGTCTG<br>TGAAGGGTAGGAGCACAATGGCGGAGATCAACAGCACCAGAATCG<br>ATCTCCAGTCCTGCTTTC
Dm-HS-2 template	/52-Bio//iSp18/AAGCTCTAGTTCGGTCTG<br>TTGAGAGAGACAGGGGCAGGACAAGGGTCAACGATGCGACCATAG<br>ATCTCCAGTCCTGCTTTC
Dm-HS-3 template	/52-Bio//iSp18/AAGCTCTAGTTCGGTCTG<br>CGAGTAGGGAAGGGGATATGGAGGAGATCAAAATCACAATAATCG<br>ATCTCCAGTCCTGCTTTC
Dm-HS-4 template	/52-Bio//iSp18/AAGCTCTAGTTCGGTCTG<br>CGAAGGGTAGGAGCACAATGGCGGAGATCAACAGCACCAGAATCG<br>ATCTCCAGTCCTGCTTTC
Dm-HS-5 template	/52-Bio//iSp18/AAGCTCTAGTTCGGTCTG<br>TGAAGGGCAGGAGCACAATGGCGGAGATCAACAGCACCAGAATCG<br>ATCTCCAGTCCTGCTTTC
Dm-HS-6 template	/52-Bio//iSp18/AAGCTCTAGTTCGGTCTG<br>AGGTCAGCGTGAACGGCATGATTAGCAGGACAGCAGTTACTACCA<br>ATCTCCAGTCCTGCTTTC
Dm-HS-7 template	/52-Bio//iSp18/AAGCTCTAGTTCGGTCTG<br>AGGTGACGGAGGTGATCAAGGGGACTAGCAACGTAACTGCAATCG<br>ATCTCCAGTCCTGCTTTC
Dm-HS-8 template	/52-Bio//iSp18/AAGCTCTAGTTCGGTCTG<br>TTATGAATGGCAATAAGGTGGGCAGGGGAGACGTAACTGCAATCG<br>ATCTCCAGTCCTGCTTTC
Dm-HS-9 template	/52-Bio//iSp18/AAGCTCTAGTTCGGTCTG<br>CGAGTAGGGAAGGGGATATGGGGGAGATCAAAATCACAATAATCG<br>ATCTCCAGTCCTGCTTTC
Dm-HS-10 template	/52-Bio//iSp18/AAGCTCTAGTTCGGTCTG<br>CGGTCAAGGGGAACAGGGGTGACAGGGAGAAAGCCGATGCGAAGG<br>ATCTCCAGTCCTGCTTTC
Dm-CVAE-without-selection-1 template	/52-Bio//iSp18/AAGCTCTAGTTCGGTCTG<br>GTAGGAGGAAAGGCAATATGGAAAAGATCAGCATAAGCGATGCGG<br>ATCTCCAGTCCTGCTTTC
Dm-CVAE-without-selection-2 template	/52-Bio//iSp18/AAGCTCTAGTTCGGTCTG<br>GGGGGAAAAGGAGCACAACTGAGAAGAGAGCAACAGCGGTAACGA<br>ATCTCCAGTCCTGCTTTC
Dm-CCS-1 template	/52-Bio//iSp18/AAGCTCTAGTTCGGTCTG<br>ACAGCAGAGGCAGCAGCAGGGTTAGGGCTGGAGCTGCCATTAAGG<br>ATCTCCAGTCCTGCTTTC

Dm-CCS-2 template	/52-Bio//iSp18/AAGCTCTAGTTCGGTCTG GGAGGACTGGAGGCAGGGCAACTGTCAAGGAGGTGGCCATAAACG ATCTCCAGTCCTGCTTTC
Dm-CCS-3 template	/52-Bio//iSp18/AAGCTCTAGTTCGGTCTG CCATGACGAGCAGCAAGGTAGGGGGCACGACTGGAGGGGATGTAA ATCTCCAGTCCTGCTTTC
Dm-CCS-4 template	/52-Bio//iSp18/AAGCTCTAGTTCGGTCTG GCGCGAGAGGCAGCAGCAAGATCACAGTCGGGATCGCTGTCAGCG ATCTCCAGTCCTGCTTTC
Dm-CCS-5 template	/52-Bio//iSp18/AAGCTCTAGTTCGGTCTG AGGATACTAAGGGCATCAGTGGTGGAACGGCTGGAGATAGTAGAA ATCTCCAGTCCTGCTTTC
Dm-reference-based-random-after-selection-1 template	/52-Bio//iSp18/AAGCTCTAGTTCGGTCTG ACAGTACCGAAGCAAATAGTAGCAGTAGCGGTACGGTGGACATGG ATCTCCAGTCCTGCTTTC
Dm-reference-based-random-after-selection-2 template	/52-Bio//iSp18/AAGCTCTAGTTCGGTCTG AGGCAAACGTTAGCAGCGGAGGGACCATGGGCATCAGGGGCACAA ATCTCCAGTCCTGCTTTC
Dm-reference-based-random-after-selection-3 template	/52-Bio//iSp18/AAGCTCTAGTTCGGTCTG GGGGTACTAAGGTGGGGGATGATAGCAACAGGGCGGGCAAGAGCA ATCTCCAGTCCTGCTTTC
Dm-reference-based-random-after-selection-4 template	/52-Bio//iSp18/AAGCTCTAGTTCGGTCTG TGGGGGGGATGGCAACGGAAACGGCCGATGCTATAAGGAAGGATG ATCTCCAGTCCTGCTTTC
Dm-reference-based-random-after-selection-5 template	/52-Bio//iSp18/AAGCTCTAGTTCGGTCTG GAAAAAGCAGTATAAAAAAGGAGATTATCGATAAGGGAGGAAATG ATCTCCAGTCCTGCTTTC

Supplementary Note 2 | Mass spectrum of negative selection target.

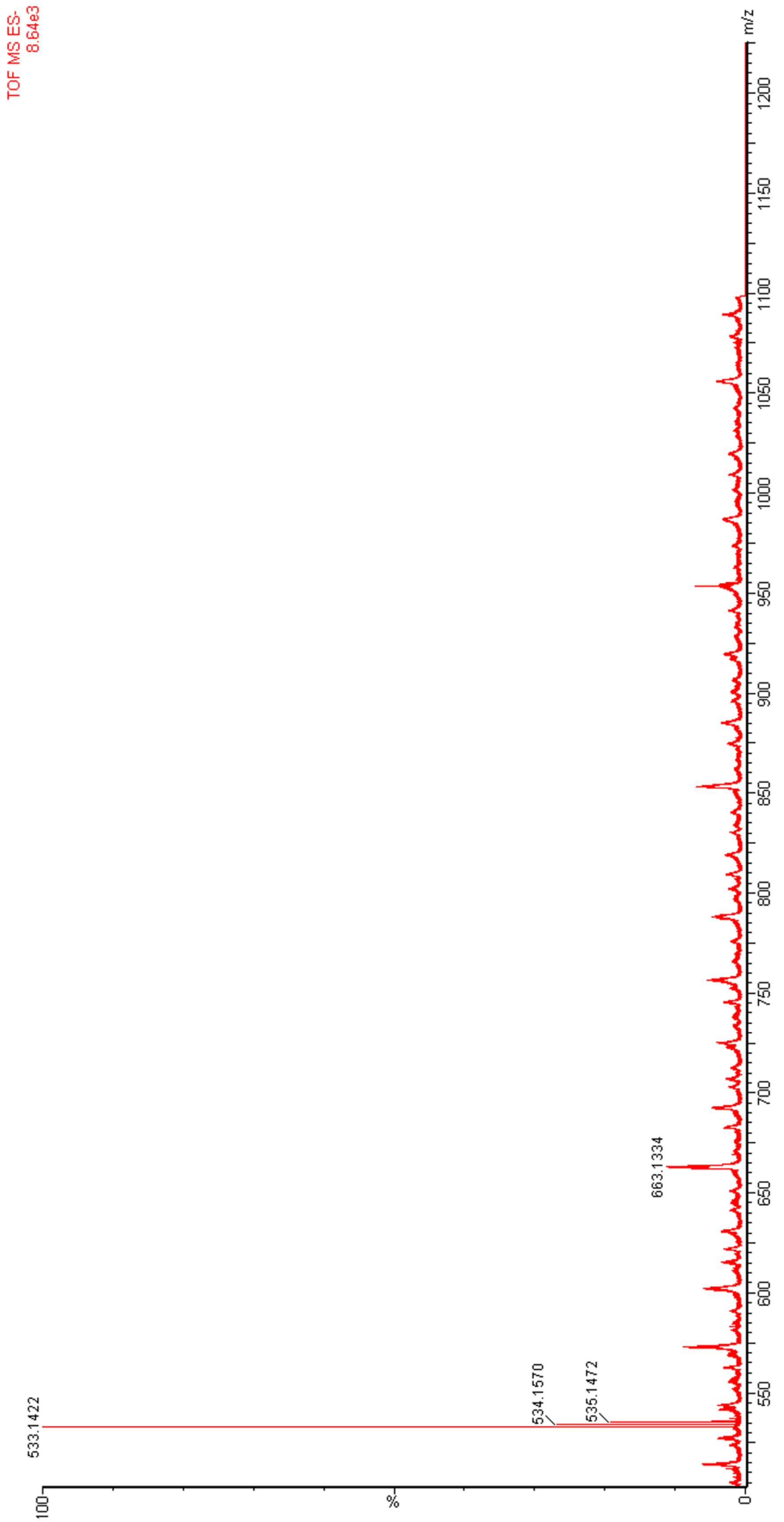

Supplementary Data 1 | Excel file with HFNAP sequences from training data, 10,000-sequence sets, and competition set.

See the separately provided file.
