# Supplementary material for: Generating experimentally unrelated target molecule-binding highly functionalized nucleic-acid polymers using machine learning
Source: Nat Commun. 2022 Aug 4;13:4541. doi: 10.1038/s41467-022-31955-4 (PMC9352670; doi:10.1038/s41467-022-31955-4)
Supplement: Supplementary file 2 — Reporting Summary [file 41467_2022_31955_MOESM2_ESM.pdf]

Corresponding author(s): David R Liu

Last updated by author(s): July 2, 2022

Reporting Summary

Nature Research wishes to improve the reproducibility of the work that we publish. This form provides structure for consistency and transparency in reporting. For further information on Nature Research policies, see our Editorial Policies and the Editorial Policy Checklist.

Statistics

For all statistical analyses, confirm that the following items are present in the figure legend, table legend, main text, or Methods section.

n/a	Confirmed	
☐	☒	The exact sample size (*n*) for each experimental group/condition, given as a discrete number and unit of measurement
☐	☒	A statement on whether measurements were taken from distinct samples or whether the same sample was measured repeatedly
☐	☒	The statistical test(s) used AND whether they are one- or two-sided<br>*Only common tests should be described solely by name; describe more complex techniques in the Methods section.*
☒	☐	A description of all covariates tested
☒	☐	A description of any assumptions or corrections, such as tests of normality and adjustment for multiple comparisons
☐	☒	A full description of the statistical parameters including central tendency (e.g. means) or other basic estimates (e.g. regression coefficient) AND variation (e.g. standard deviation) or associated estimates of uncertainty (e.g. confidence intervals)
☐	☒	For null hypothesis testing, the test statistic (e.g. F, t, r) with confidence intervals, effect sizes, degrees of freedom and P value noted<br>*Give P values as exact values whenever suitable.*
☐	☒	For Bayesian analysis, information on the choice of priors and Markov chain Monte Carlo settings
☒	☐	For hierarchical and complex designs, identification of the appropriate level for tests and full reporting of outcomes
☐	☒	Estimates of effect sizes (e.g. Cohen's *d*, Pearson's *r*), indicating how they were calculated

Our web collection on statistics for biologists contains articles on many of the points above.

Software and code

Policy information about availability of computer code

Data collection	High-throughput sequencing was filtered by AptaSuite v0.9.6. MST was collected using standard software from NanoTemper (M.O Screening v1.9).
Data analysis	As described in the Methods section, high throughput sequencing was analyzed by AptaSuite v0.9.6, which includes the AptaTrace package. Models were built using Python 3.6 and PyTorch 1.3.1. Data was analyzed using custom Python scripts with the following package versions: UMAP v0.4, numpy v1.18, pandas v0.25, seaborn v0.11, matplotlib v3.1, sci-kit-bio v0.5, sci-kit-learn v0.22. Data was analyzed using Graphpad Prism 9. MST was analyzed using MO.Affinity Analysis v2.3. Secondary structures were analyzed using RNAstructure v6.2, which includes the multilign package, and ViennaRNA v2.4, which includes the RNAalifold, RNAeval, and RNAdistance packages. Custom code is available upon request.

For manuscripts utilizing custom algorithms or software that are central to the research but not yet described in published literature, software must be made available to editors and reviewers. We strongly encourage code deposition in a community repository (e.g. GitHub). See the Nature Research guidelines for submitting code & software for further information.

Data

Policy information about availability of data

All manuscripts must include a data availability statement. This statement should provide the following information, where applicable:

- Accession codes, unique identifiers, or web links for publicly available datasets
- A list of figures that have associated raw data
- A description of any restrictions on data availability

The principal data supporting the findings of this work are available in the main text or the supplementary materials. High-throughput sequencing data will be available from the NCBI Sequence Read Archive under accession code PRJNA854957. Data used for training has been included in the Supplementary Information.

Additional data and code that support the findings of this study are available from the authors on request.

Field-specific reporting

Please select the one below that is the best fit for your research. If you are not sure, read the appropriate sections before making your selection.

☒ Life sciences ☐ Behavioural & social sciences ☐ Ecological, evolutionary & environmental sciences

For a reference copy of the document with all sections, see nature.com/documents/nr-reporting-summary-flat.pdf

Life sciences study design

All studies must disclose on these points even when the disclosure is negative.

Sample size	No sample size calculation was performed. Samples sizes were chosen such that sufficient data could be acquired to accurately estimate differences between groups if differences did exist.
Data exclusions	No data were excluded except when experiments failed. The MST value for the 7.81 nM concentration for Dm-HS-8 in Fig. 3c was removed because one of the replicates had a defect in the glass capillary which resulted in an inaccurate reading (outlier). The binding curve for Dm-HS-8 is fit using nine triplicate concentrations and results in similar binding affinities if the single replicate value was removed as an outlier.
Replication	Selection experiments were not replicated, as is standard in the field. Our total sequence space is on the order of 10^22 and our initial libraries sample start with a 10^13 sample of a 10^22 space. Therefore, replicates of the selection are unlikely to sample from the same sequence space. MST binding characterization were replicated using three separate serial dilutions, with each dilution series and sample inclusion individually mixed, resulting in reproducible binding curves (Fig. 2b, 3c, 3d, 4a). All attempts at replication were successful.
Randomization	The selection begins with a random sample of 10^13 sequences from the total sequence space of 10^22. Sampling is fully random from the starting set of possible sequences. Data splitting for model training was done randomly, with sequences randomly assigned to either the training set or test set. Random sequences used to compare against CVAE-generated sequences or reference-set sequences were generated using custom python scripts and built-in functions for random sampling. Randomly chosen CVAE-generated sequences or reference-based random sequences were chosen using built-in functions for random sampling. All other experimental groups were chosen based on empirical evidence, including enrichment values.
Blinding	Blinding was not relevant to our study.

Reporting for specific materials, systems and methods

We require information from authors about some types of materials, experimental systems and methods used in many studies. Here, indicate whether each material, system or method listed is relevant to your study. If you are not sure if a list item applies to your research, read the appropriate section before selecting a response.

Materials & experimental systems

n/a	Involved in the study	
☒	☐	Antibodies
☒	☐	Eukaryotic cell lines
☒	☐	Palaeontology and archaeology
☒	☐	Animals and other organisms
☒	☐	Human research participants
☒	☐	Clinical data
☒	☐	Dual use research of concern

Methods

n/a	Involved in the study	
☒	☐	ChIP-seq
☒	☐	Flow cytometry
☒	☐	MRI-based neuroimaging
